# Supplementary material for: Management of possible serious bacterial infection in young infants closer to home when referral is not feasible: Lessons from implementation research in Himachal Pradesh, India
Source: PLoS One. 2020 Dec 22;15(12):e0243724. doi: 10.1371/journal.pone.0243724 (PMC7755274; doi:10.1371/journal.pone.0243724)
Supplement: S1 Form — (PDF) [file pone.0243724.s001.pdf]

Country ID:	Site ID	Line-list ID:

List of pregnant women and home visit record **1**

Implementation Research on Management of Possible Serious Bacterial Infection (PSBI) in Young Infants (0-59 Days) Where Referral is Not Feasible

1.CHC	2. PHC	3. SC	4. ASHA's Name	5. ANM's Name

6. No	7. Name of the pregnant woman	8. Age (Yrs)	9. Husband's Name	10. Village/Address	11. LMP Date 99/99/999 = not captured	12. Expected date of birth (EDD) 99/99/9999 = not captured	13 Date of pregnancy registration 99/99/9999 = not captured	14. ANC Visits 99/99/9999 = not captured				15 Pregnancy outcome	16 Date of pregnancy outcome 99/99/9999 = not captured	17 Place of birth/ miscarriage	18 Birth attendant	19 Status of mother (On the day of data collection by Project Staff)	20 Status of newborn (On the day of data collection by Project Staff)	21 Date of outcome documentation (For project staff)	22. Date of outcome verification (For Project Staff)
								1st	2nd	3rd	4th								

	Collected By	☐☐ (Initial of name only)	Date *(dd/mm/yyyy)*	☐☐/☐☐/☐☐☐☐
	Checked by	☐☐ (Initial of name only)	Date *(dd/mm/yyyy)*	☐☐/☐☐/☐☐☐☐
	Entered by	☐☐ (Initial of name only)	Date *(dd/mm/yyyy)*	☐☐/☐☐/☐☐☐☐

CODES:

15 **Pregnancy outcome**	17 **Place of birth/ miscarriage**	18 **Birth attendant**	19 **Status of mother**	20 **Status of newborn**
01 = Miscarriage<br>02= Stillbirth<br>03= Live birth<br>99= not captured	01= Home,<br>02= Health facility (Govt)<br>03= Private<br>88= Other,<br>99= Not captured	01= Doctor/nurse / ANM,<br>02= TBA,<br>03.= Unattended<br>88=Other-(specify,)<br>99= Not captured	01 = Alive<br>02= Dead,<br>03=Hospitalized<br>99= Not captured	01 = Alive<br>02= Dead,<br>03=Hospitalized<br>99 = Not captured
