# Supplementary material for: Management of possible serious bacterial infection in young infants closer to home when referral is not feasible: Lessons from implementation research in Himachal Pradesh, India
Source: PLoS One. 2020 Dec 22;15(12):e0243724. doi: 10.1371/journal.pone.0243724 (PMC7755274; doi:10.1371/journal.pone.0243724)
Supplement: S2 Form — (PDF) [file pone.0243724.s002.pdf]

Country ID:	Site ID:	FORM ID:

POSTNATAL (HBPNC) HOME VISIT RECORD 2

Implementation Research On Management Of Possible Serious Bacterial Infection (PSBI) In Young Infants (0-59 Days) Where Referral Is Not Feasible

1. PHC -	2. Sub-centre-	3. Village-	4. ASHA -
5. Mother-	6. Father-	7. Religion- Hindu / Muslim / Sikh / Other	8. Caste- SC / Other

9. Visit for Pregnancy preparation- / /	10. Ph. No of preg. lady - / / / / / / / / /	11.Date of admission for delivery - / / /
12.Date of Delivery -	13. Place of delivery – Dist. Hospital. / CHC / PHC / Sub-centre / Pvt. / home / road / other /NC	14.Type of delivery- Normal / Caesarean / assisted / NC
15.Time of Delivery / AM/PM	16. Outcome - live birth / Still birth / died after birth / NC / NAS	17. Birth weight (in grms)-
18.Sex of Child - Male / Female/ Other / NC	19. When infant breastfed for first time- First hour / first day / after 1st day / did not / NC	20.Hospital discharge date (Mother)- / /
21.Hospital discharge date (Child) - / /	22.Child's MCTS No; / / / / / / / / / / /	23. Mother's MCTS No- / / / / / / / / / / /
24.Infants Name(Ask):		

(Q 25 to Q 27: TO be filled if any supervision visits were made for this infant)

25. Supervisory visit – Yes / No	26.Designation of Supervisor–	27. Date of supervision- / /

Description of assessment by ASHAs during HBPNC visit

(Code Abbreviation= NC = Not captured; R= Right ; W = Wrong)

Visit number	28.Visit 1	29.Visit 2	30.Visit 3	31.Visit 4	32.Visit 5	33.Visit 6	34.Visit 7	35.Visit 8	36.Sup. Visit (fill up, if visit is made)
Day after delivery	1day	3rd day	7th day	14th Day	21st day	28th day	42nd day	59th Day	
I. Date of visit	/ /	/ /	/ /	/ /	/ /	/ /	/ /	/ /	
II. Baby alive (If response yes , skip to V)	Yes / No / NC	Yes / No / NC	Yes / No / NC	Yes / No / NC	Yes / No / NC	Yes / No / NC	Yes / No / NC	Yes / No / NC	R / W / NC
III. If Died, date of death	/ /	/ /	/ /	/ /	/ /	/ /	/ /	/ /	R / W / NC
IV. Place of Death (Institutional / Private/ other - elaborate)									R / W / NC
V. Infant was clothed according to weather?	Yes / No / NC	Yes / No / NC	Yes / No / NC	Yes / No / NC	Yes / No / NC	Yes / No / NC	Yes / No / NC	Yes / No / NC	R / W / NC
VI. Bathed?	Yes / No / NC								R / W / NC
VII. Supplementary feeding?	Yes / No / NC	Yes / No / NC	Yes / No / NC	Yes / No / NC	Yes / No / NC	Yes / No / NC	Yes / No / NC	Yes / No / NC	R / W / NC

Country ID:	Site ID:	FORM ID:

POSTNATAL (HBPNC) HOME VISIT RECORD **2**

Implementation Research On Management Of Possible Serious Bacterial Infection (PSBI) In Young Infants (0-59 Days) Where Referral Is Not Feasible

Visit number	28.Visit 1	29.Visit 2	30.Visit 3	31.Visit 4	32.Visit 5	33.Visit 6	34.Visit 7	35.Visit 8	36.Sup. Visit
VIII. Complete breast feeding (5-6 times in day and 3/4 times in night)	Yes / No / NC	Yes / No / NC	Yes / No / NC	Yes / No / NC	Yes / No / NC	Yes / No / NC	Yes / No / NC	Yes / No / NC	R / W / NC
IX. Weight of the infant, in grams (write 9999 if not documented)									R / W / NC
X. Temperature of the infant, in° F (write 999.9 if not documented)									R / W / NC
XI. Counted respiratory rate/min (write 99 if not documented)									R / W / NC
XII. Was umbilicus draining pus?	Yes / No / NC	Yes / No / NC	Yes / No / NC	Yes / No / NC	Yes / No / NC	Yes / No / NC	Yes / No / NC	Yes / No / NC	R / W / NC
XIII. Was Eyes draining pus?	Yes / No / NC	Yes / No / NC	Yes / No / NC	Yes / No / NC	Yes / No / NC	Yes / No / NC	Yes / No / NC	Yes / No / NC	R / W / NC
XIV. < 10 skin pustules?	Yes / No / NC	Yes / No / NC	Yes / No / NC	Yes / No / NC	Yes / No / NC	Yes / No / NC	Yes / No / NC	Yes / No / NC	R / W / NC
	(If following signs were present ASHAs are mandated to inform ANM /MOs OR immediately call 102 ambulance and refer the infant to nearest health facility)								
XV. Convulsions?	Yes / No / NC	Yes / No / NC	Yes / No / NC	Yes / No / NC	Yes / No / NC	Yes / No / NC	Yes / No / NC	Yes / No / NC	R / W / NC
XVI. Respiratory rate $\geq$ 60 (Fast breathing)	Yes / No / NC	Yes / No / NC	Yes / No / NC	Yes / No / NC	Yes / No / NC	Yes / No / NC	Yes / No / NC	Yes / No / NC	R / W / NC
XVII. Chest indrawing?	Yes / No / NC	Yes / No / NC	Yes / No / NC	Yes / No / NC	Yes / No / NC	Yes / No / NC	Yes / No / NC	Yes / No / NC	R / W / NC
XVIII. Stopped feeding/ unable to feed well?	Yes / No / NC	Yes / No / NC	Yes / No / NC	Yes / No / NC	Yes / No / NC	Yes / No / NC	Yes / No / NC	Yes / No / NC	R / W / NC
XIX. Body temp. $\geq$ 99^0 F OR < 95^0 F	Yes / No / NC	Yes / No / NC	Yes / No / NC	Yes / No / NC	Yes / No / NC	Yes / No / NC	Yes / No / NC	Yes / No / NC	R / W / NC
XX. Movement on simulation/ No movement / less movement	Yes / No / NC	Yes / No / NC	Yes / No / NC	Yes / No / NC	Yes / No / NC	Yes / No / NC	Yes / No / NC	Yes / No / NC	R / W / NC
XXI. Low body weight	< 2000gms		< 2100gms	< 2100gms	< 2200gms	<2300gms	<2500gms	<2500gms	<2500gms
	Yes / No / NC	Yes / No / NC	Yes / No / NC	Yes / No / NC	Yes / No / NC	Yes / No / NC	Yes / No / NC	Yes / No / NC	R / W / NC
XXII. $\geq$ 10 Skin pustules Or a big Boil	Yes / No / NC	Yes / No / NC	Yes / No / NC	Yes / No / NC	Yes / No / NC	Yes / No / NC	Yes / No / NC	Yes / No / NC	R / W / NC
XXIII. Palms/soles/ body yellow?	Yes / No / NC	Yes / No / NC	Yes / No / NC	Yes / No / NC	Yes / No / NC	Yes / No / NC	Yes / No / NC	Yes / No / NC	R / W / NC
XXIV. Lips/ face/ Body became blue	Yes / No / NC	Yes / No / NC	Yes / No / NC	Yes / No / NC	Yes / No / NC	Yes / No / NC	Yes / No / NC	Yes / No / NC	R / W / NC
XXV. Umbilicus bleeding	Yes / No / NC	Yes / No / NC	Yes / No / NC	Yes / No / NC	Yes / No / NC	Yes / No / NC	Yes / No / NC	Yes / No / NC	R / W / NC
XXVI. Did not urinate within 24 hrs of birth	Yes / No / NC								R / W / NC
XXVII. Did not passed stool within 48 hrs of birth	Yes / No / NC	Yes / No / NC							R / W / NC
XXVIII. Blood in stool	Yes / No / NC	Yes / No / NC	Yes / No / NC	Yes / No / NC	Yes / No / NC	Yes / No / NC	Yes / No / NC	Yes / No / NC	R / W / NC
XXIX. Watery diaherroea more than 3 times in a day with symptoms of dehydration	Yes / No / NC	Yes / No / NC	Yes / No / NC	Yes / No / NC	Yes / No / NC	Yes / No / NC	Yes / No / NC	Yes / No / NC	R / W / NC

Country ID:	Site ID:	FORM ID:

POSTNATAL (HBPNC) HOME VISIT RECORD 2

Implementation Research On Management Of Possible Serious Bacterial Infection (PSBI) In Young Infants (0-59 Days) Where Referral Is Not Feasible

Fill this section if the infant is referred By ASHA and the information is documented in HBPNC form

37.Did ASHA refer the infant?	38. If yes, date of referral	39. Place of referral	40.Was the infant taken for treatment	41. If yes, did the family use 102 service?	42. If yes, where was he infant taken for treatment?	43. If not, what was the reason	44. Outcome	45.Supervisor's check
Yes / No / NC	/ /		Yes / No (if no, skip to 45)	Yes/ No	SNCU / Other Govt. Institution/ Private/ other	Ambulance not available/ can not decide / lack of cash/ home remedy/ other	Healthy/ Sick/ died/ NC	R/ W / NC

Verification of ASHA's HBNC Visits

	Verification	46.Pre delivery visit Verification	47.Day 1 Verification	48.Day 3 Verification	49.Day 7 Verification	50.Day 14 Verification	51.Day 21 Verification	52.Day 28 Verification	53.Day 42 Verification	54.Day 59 Verification	55.Supervisor's check
I.	Signed by Mother	Yes / No	Yes / No	Yes / No	Yes / No	Yes / No	Yes / No	Yes / No	Yes / No	Yes / No	R/ W / NC
II.	Date of verification by mother (write 99/99/99 if no date)	/ /	/ /	/ /	/ /	/ /	/ /	/ /	/ /	/ /	
III.	Signed by ASHA supervisor	Yes / No	Yes / No	Yes / No	Yes / No	Yes / No	Yes / No	Yes / No	Yes / No	Yes / No	
IV.	Date of verification by ASHA supervisor (write 99/99/99 if no date)	/ /	/ /	/ /	/ /	/ /	/ /	/ /	/ /	/ /	

Fill this section if the Family moved away

56.Migrated – Yes / No	57.Date of Migration-	58.Place of Migration (ADDRESS)-	59.Name of the HH head (where migrated)-

60.	Completed by	☐☐ (Initial of name only)	Date *(dd/mm/yyyy)*	☐☐/☐☐/☐☐☐☐	61.	Entered by	☐☐ (Initial of name only)	Date *(dd/mm/yyyy)*	☐☐/☐☐/☐☐☐☐
62.	Checked by	☐☐ (Initial of name only)	Date *(dd/mm/yyyy)*	☐☐/☐☐/☐☐☐☐	63.	Entered by	☐☐ (Initial of name only)	Date *(dd/mm/yyyy)*	☐☐/☐☐/☐☐☐☐
