# Supplementary material for: Management of possible serious bacterial infection in young infants closer to home when referral is not feasible: Lessons from implementation research in Himachal Pradesh, India
Source: PLoS One. 2020 Dec 22;15(12):e0243724. doi: 10.1371/journal.pone.0243724 (PMC7755274; doi:10.1371/journal.pone.0243724)
Supplement: S3 Form — (PDF) [file pone.0243724.s003.pdf]

Site ID:	Country ID:	Form ID:	Sick Young Infant Assessment Form	3

Implementation Research on Management of Possible Serious Bacterial Infection (PSBI) in Young Infants (0-59 Days) where Referral is Not Feasible

A. Infants Name .... B. Mother's Name .... C. Father's Name............................

D. Village.. E. Family Phone No ☐☐☐☐☐☐☐☐☐☐ F. ASHAs Name:………………..

F. Assessor's Name .... G. Infant's problems (as mentioned by mother/caregiver)..

....

(This section NOT to be entered in database)

1. Infant's ID Number:	2. Age: ☐☐ days	3. Weight: ☐.☐ kg	4. Temperature: ☐☐.☐°C
5. First visit for this illness - Yes / No	6.Assesment by - MO / Nurse / ANM / Others	Others-	
7. Place of Assessment - Home / PHC / CHC / DH	8. PHC -	9. SC -	
10. Assessment date - / /	11. Assessment time - ☐☐.☐☐ AM/ PM		

SECTION A

This section will capture information on the signs which health worker documented to assess and classify sick newborn. The signs which are mentioned below, are also mentioned in " Treatment card: Management of Sepsis in Young Infant

A 1. Checked For Possible Serious Bacterial Infection

	Assess	Encircle the response
12.	Not able to feed or No breast attachment or not suckling at all	Yes / No
13.	Less than normal movement	Yes / No
14.	Lethargic or unconscious	Yes / No
15.	Convulsions (fits)	Yes / No
16.	Fast breathing ($\geq$ 60/mins)	Yes / No
17.	Severe chest indrawing	Yes / No
18.	Nasal flaring?	Yes / No
19.	Grunting?	Yes / No
20.	More than 10 skin postules or a big boil	Yes / No
21.	Axillary temperature $\geq$ 37.5 °C OR temperature < 35.5°C	Yes / No
22.	Blood in stool	Yes / No
23.	Infant classified as<br>(As mentioned in Annexure section of Treatment card)	Critically ill....................................01<br>Severe Clinical Infection................02<br>Severe Pneumonia........................03<br>Pneumonia....................................04<br>Local Infection................................05<br>None of the above..........................06<br>Not captured..................................99

SECTION B

*The section gets populated, if Health workers /medical officer are using IMNCI assessment form to classify cases as per the state mandate***.**

**24.**	.Did the assessor use IMNCI recording form?	Yes.......................................01<br>No.......................................02	 **Skip to Sec. C**

B- 1. Checked For PSBI OR Very Severe Disease and Local Infection

	Assess	Encircle the response	Skips
**25.**	Is the infant having difficulty in feeding	Yes / No	
**26.**	Convulsions	Yes / No	
**27.**	Breath count (RR) in one minute	☐☐ (write 99 if not captured)	
**28.**	Repeat breath count	☐☐ (write 99 if not captured)	
**29.**	Severe chest indrawing	Yes / No	
**30.**	Nasal Flaring	Yes / No	
**31.**	Grunting	Yes / No	
**32.**	Body temperature	Fever ($\geq$ 37.5°C)$_1$/ low temp. (< 35.5°C)$_2$	
**33.**	Look at Infant's movement	Moves on own / When shimulated / No movement /less than normal / Unconcious	

Version 4

**34.**	Umbilicus draining pus	Yes / No	
**35.**	Umbilicus is red	Yes / No	
**36.**	Skin pustules	_Yes / No	If no, skip to 38
**37.**	Skin pustules	>10 / One Big pustule / < 10	
**38.**	Was infant also classified for local infection	Only PSBI Or VSD ....01<br>Only Local Infection.......02<br>Both PSBI and LI...........03<br>Fast Breathing…………04<br>PSBI OR LI unlikely…....05<br>Not captured 99	
	**B-2. Checked For Jaundice**		
	**Assess**	**Encircle the response**	**Skips**
**39.**	Did the infant assessed for jaundice?	Yes / No	**If No, Skip to 44**
**40.**	When did the jaundice appear first?	Within 24 hrs of birth / Affer 24 hrs of birth / Not captured	
**41.**	Yellow skin?	Yes / No	
**42.**	Were palms or soles yellow?	Yes / No	
**43.**	Was the infant classified for jaundice?	Severe Jaundice...........01<br>Jaundice.......................02<br>No Jaundice..................03<br>Not captured.................99	
	**B-3. Asked For Diarrhoea and Looked For Dehydration (In Case Of Diarrhoea)**		
	**Assess**	**Encircle the response**	**Skips**
**44.**	Did the infant assessed for diarrhoea?	Yes / No	**If No, skip to 52**
**45.**	Did the infant have diarrhoea?	Yes / No / Not captured	**If No, skip to 52**
**46.**	Did the infant has blood in stool	Yes / No / Not captured	
**47.**	For how long ?	☐☐ days (99 if not captured)	
**48.**	How was the infant's movement?	Restless or irritable / No movement / Only when stimulated / unconscious	
**49.**	Were eyes sunken?	Yes / No	
**50.**	Pinch the skin of the abdomen. Does it go back	Very slowly / Slowly	
**51.**	Was the infant classified for Dehydration?	Severe dehydration........01<br>Some dehydration...........02<br>No dehydration...............03<br>Not captured...................99	
	**B-4. Checked For Feeding Problem Or Low Weight For Age**		
	**Assess**	**Encircle the response**	**Skips**
**52.**	Did the infant assessed for feeding Prob. Or low weight?	Yes / No	**If No, skip to 61**
**53.**	Is the infant breastfed?	Yes / No / Not captured	
**54.**	How many times in 24 hours the infant was breastfed?	☐☐ (99 if not captured)	
**55.**	Does the infant usually receive any other foods/drinks?	Yes / No / Not captured	**If No, skip to 58**
**56.**	If other food/drinks, how often (in 24 hrs)?	☐☐(99 if not captured	
**57.**	If yes, what do you use to feed the infant?	Cup / Bottle / spoon / other / Not captured	If other, write-
**58.**	Determine weight for age. Is the weight low?	Very low / Low / Not low weight / NC	
**59.**	Ulcers or white patches in mouth (thrush)?	Yes / No	
**60.**	Was the infant classified for having feeding problem or Low weight for age?	Very Low weight …………………………01<br>Feeding problem OR/and Low weight…..02<br>No feeding problem………………………03<br>Not captured……………………………99	
	**B-5. Assessed Breastfeeding**<br>*(Health workers should feel this section only if there is a feeding difficulty / taking other food or drinks /low weight for age / **has no indications to***		

refer urgently to hospital)			
	**Assess**	**Encircle the correct response**	**Skips**
**61.**	Was the infant assess for breastfeeding?	Yes / No	**If no, Skip to 70**
**62.**	Has the infant breastfed in the previous hour?	Yes / No / Not captured	**If No, Skip to 64**
**63.**	If breastfed in previous hour, was feeding assessed further?	Yes / No	**If no, Skip to 70**
64.	More areola seen above top lip than below bottom lip?	Yes / No / Not captured	
65.	Mouth wide open	Yes / No / Not captured	
66.	Lower lip turned outwards	Yes / No / Not captured	
67.	Chin touching breast	Yes / No / Not captured	
68.	How the infant was attached to breast?	Good / Poor / No attachment / Not captured	
69.	How the infant was suckling	Effectively / Not effectively / Not suckling atall / Not captured	
**B- 6. Check Infant's Immunization Status**			
	**Assess**	**Encircle the correct response**	**Skips**
70.	BCG 0	Yes / No / Not captured	
71.	Hep B0	Yes / No / Not captured	
72.	OPV 0	Yes / No / Not captured	
73.	Pentavalent 1	Yes / No / Not captured	
74.	Hep B1		
75.	OPV 1	Yes / No / Not captured	
76.	Rotavirus 1	Yes / No / Not captured	
77.	PCV 1	Yes / No / Not captured	
78.	What was next date of immunization	☐☐/☐☐/☐☐	

**Section C: (Mandatory)** The information for this section shall be sourced from "Treatment card: Management of Sepsis in Young Infant". ANMS are mandated to refer all PSBI cases to hospitals and if they refuse referral then ANM /MO should offer an outpatient 7 days PSBI treatment regimen which should be documented in PSBI treatment card.			
	**Assess**	**Response**	**Skips**
79.	Advised on referral to nearest HF and care during referral?	Yes / No / Not captured	If no, STOP Here
80.	Write the name of the facility to which referral was made (Ask health worker)		
81.	Pre-referral dose of antibiotics given	Yes / No / Not captured	If Yes , Skip to 84
82.	If no pre-referral dose given, ask for the reason<br><br>(ASK health worker)	Dose not available........................01<br>Syringe not available....................02<br>Not confident to give the dose......03<br>Refused ....04<br>Other 88	
83.	If pre-referral dose refused / other, what was the reason? (ASK health worker)		
84.	Informed young infant's condition to MO/SN	Yes / No / Not captured / NA	
85.	Patient refused referral	Yes / No / Not captured	If NO, STOP Here<br>If Yes, Skip to 88
86.	If refused, what was the reason<br><br>(ASK health worker)	Hospital is too far...............................01<br>Lack of time if hospitalized.................02<br>Cannot afford transport.......................03<br>Cannot afford hospitalization..............04<br>Undecided ....05<br>Other..88	
87.	If other , please elaborate the reason (ASK health worker)		
88.	Did health-worker offered the infant 7 days Outpatient simple treatment? (ASK health worker)	Yes / No	If yes skip to 90

89.	If no, what was the reason (ASK health worker)		
90.	Did the family accept the treatment (ASK health worker)	Yes / No	If Yes STOP HERE
91.	If no, what was the reason?<br>(ASK health worker)		

92.	Form completed by	☐☐ (Initial of name only)	Date *(dd/mm/yyyy)*	☐☐/☐☐/☐☐☐☐
93.	Form checked by	☐☐ (Initial of name only)	Date *(dd/mm/yyyy)*	☐☐/☐☐/☐☐☐☐
94.	Form entered by	☐☐ (Initial of name only)	Date *(dd/mm/yyyy)*	☐☐/☐☐/☐☐☐☐
95.	Form entered by	☐☐ (Initial of name only)	Date *(dd/mm/yyyy)*	☐☐/☐☐/☐☐☐☐

END of THH FORM
