# Supplementary material for: Management of possible serious bacterial infection in young infants closer to home when referral is not feasible: Lessons from implementation research in Himachal Pradesh, India
Source: PLoS One. 2020 Dec 22;15(12):e0243724. doi: 10.1371/journal.pone.0243724 (PMC7755274; doi:10.1371/journal.pone.0243724)
Supplement: S4 Form — (PDF) [file pone.0243724.s004.pdf]

Country ID	Site ID	Form ID

FACILITY FOLLOW UP FORM 4

Implementation Research on Management of Possible Serious Bacterial Infection (PSBI) In Young Infants (0-59 Days) Where Referral Is Not Feasible

[To be filled by the research team from the infant treatment card and register maintained at the health centre]

INFANT AND HEALTH WORKER IDENTIFYING INFORMATION				
1. CHC	2. PHC	3. Sub-Centre	4. Village	5. Infant ID:

GENERAL												
Day number				Day 1	Day 2	Day 3	Day 4	Day 5	Day 6	Day 7		
6.	Date of follow-up visit *(dd/mm/yyyy)*			/ /	/ /	/ /	/ /	/ /	/ /	/ /		
7.	Time of follow-up visit *(hh:mm,)*			AM/PM	AM/PM	AM/PM	AM/PM	AM/PM	AM/PM	AM/PM	AM/PM	AM/PM
8.	Follow-up carried out at	1	Health facility	SC / PHC / CHC / DH	SC / PHC / CHC / DH	SC / PHC / CHC / DH	SC / PHC / CHC / DH	SC / PHC / CHC / DH	SC / PHC / CHC / DH	SC / PHC / CHC / DH	SC / PHC / CHC / DH	SC / PHC / CHC / DH
		2	Home	Yes / No	Yes / No	Yes / No	Yes / No	Yes / No	Yes / No	Yes / No	Yes / No	Yes / No
9.	Visit status (home visit) (one answer only)	1.	Infant assessed during visit	Yes / No	Yes / No	Yes / No	Yes / No	Yes / No	Yes / No	Yes / No	Yes / No	Yes / No
		2	Infant temporarily away from home	Yes / No	Yes / No	Yes / No	Yes / No	Yes / No	Yes / No	Yes / No	Yes / No	Yes / No
		3	Nobody at home	Yes / No	Yes / No	Yes / No	Yes / No	Yes / No	Yes / No	Yes / No	Yes / No	Yes / No
		4	Refused to be assessed	Yes / No	Yes / No	Yes / No	Yes / No	Yes / No	Yes / No	Yes / No	Yes / No	Yes / No
		5	Infant died	Yes / No	Yes / No	Yes / No	Yes / No	Yes / No	Yes / No	Yes / No	Yes / No	Yes / No
		6	Infant is hospitalized	Yes / No	Yes / No	Yes / No	Yes / No	Yes / No	Yes / No	Yes / No	Yes / No	Yes / No
		7	Other (specify)	Yes / No	Yes / No	Yes / No	Yes / No	Yes / No	Yes / No	Yes / No	Yes / No	Yes / No
10.	Adult primary respondent			Father/ Mother /Other	Father/ Mother /Other	Father/ Mother /Other	Father/ Mother /Other	Father/ Mother /Other	Father/ Mother /Other	Father/ Mother /Other	Father/ Mother /Other	Father/ Mother /Other
	10.1.	If other specify:										
11.	How is the infant today? (Respondent's perception of infant's condition)			Same / Better /Worsened/ Cured / Died/ Don't know	Same / Better /Worsened / Cured / Died/ Don't know	Same / Better /Worsened / Cured / Died/ Don't know	Same / Better /Worsened / Cured / Died/ Don't know	Same / Better /Worsened / Cured / Died/ Don't know	Same / Better /Worsened / Cured / Died/ Don't know	Same / Better /Worsened / Cured / Died/ Don't know	Same / Better /Worsened / Cured / Died/ Don't know	Same / Better /Worsened / Cured / Died/ Don't know

Country ID	Site ID	Form ID

FACILITY FOLLOW UP FORM **4**

	Day number	Day 1	Day 2	Day 3	Day 4	Day 5	Day 6	Day 7		
**SIGNS OF PSBI (Y = Yes , N= No , NC = Not captured)**										
12.	Not able to feed or No breast attachment or not suckling at all	Y / N / NC	Y / N / NC	Y / N / NC	Y / N / NC	Y / N / NC	Y / N / NC	Y / N / NC	Y / N / NC	Y / N / NC
13.	Less than normal movement	Y / N / NC	Y / N / NC	Y / N / NC	Y / N / NC	Y / N / NC	Y / N / NC	Y / N / NC	Y / N / NC	Y / N / NC
14.	Lethargic or unconscious	Y / N / NC	Y / N / NC	Y / N / NC	Y / N / NC	Y / N / NC	Y / N / NC	Y / N / NC	Y / N / NC	Y / N / NC
15.	Convulsions (fits)	Y / N / NC	Y / N / NC	Y / N / NC	Y / N / NC	Y / N / NC	Y / N / NC	Y / N / NC	Y / N / NC	Y / N / NC
16.	Fast breathing (≥ 60/mins)	Y / N / NC	Y / N / NC	Y / N / NC	Y / N / NC	Y / N / NC	Y / N / NC	Y / N / NC	Y / N / NC	Y / N / NC
17.	Severe chest indrawing	Y / N / NC	Y / N / NC	Y / N / NC	Y / N / NC	Y / N / NC	Y / N / NC	Y / N / NC	Y / N / NC	Y / N / NC
18.	Nasal Flaring	Y / N / NC	Y / N / NC	Y / N / NC	Y / N / NC	Y / N / NC	Y / N / NC	Y / N / NC	Y / N / NC	Y / N / NC
19.	Grunting	Y / N / NC	Y / N / NC	Y / N / NC	Y / N / NC	Y / N / NC	Y / N / NC	Y / N / NC	Y / N / NC	Y / N / NC
20.	More than 10 skin pustules or a big boil	Y / N / NC	Y / N / NC	Y / N / NC	Y / N / NC	Y / N / NC	Y / N / NC	Y / N / NC	Y / N / NC	Y / N / NC
21.	Axillary temperature ≥ 37.5 °C OR temperature < 35.5°C	Y / N / NC	Y / N / NC	Y / N / NC	Y / N / NC	Y / N / NC	Y / N / NC	Y / N / NC	Y / N / NC	Y / N / NC
22.	Blood in stool	Y / N / NC	Y / N / NC	Y / N / NC	Y / N / NC	Y / N / NC	Y / N / NC	Y / N / NC	Y / N / NC	Y / N / NC

SIMPLIFIED ANTIBIOTIC TREATMENT GIVEN
IF TREATMENT FAILURE OR SEVERE ADVERSE EVENT NEEDING REFERRAL/CHANGE OF THERAPY PUT DOUBLE LINE THROUGH REMAINING DAYS

23.	Gentamicin	Dose	[ ][ ].[ ][ ] mL	Y / N / Partially / DNK	Y / N / Partially / DNK	Y / N / Partially / DNK	Y / N / Partially / DNK	Y / N / Partially / DNK	Y / N / Partially / DNK	Y / N / Partially / DNK	Y / N / Partially / DNK	Y / N / Partially/ DNK
24.	Ampicillin<br>Morning dose	Dose	[ ][ ].[ ][ ] mL	Y / N / Partially / DNK	Y / N / Partially / DNK	Y / N / Partially / DNK	Y / N / Partially / DNK	Y / N / Partially / DNK	Y / N / Partially / DNK	Y / N / Partially / DNK	Y / N / Partially / DNK	Y / N / Partially / DNK
25.	Ampicillin<br>Evening dose	Dose	[ ][ ].[ ][ ] mL	Y / N / Partially / DNK	Y / N / Partially / DNK	Y / N / Partially / DNK	Y / N / Partially / DNK	Y / N / Partially / DNK	Y / N / Partially / DNK	Y / N / Partially / DNK	Y / N / Partially / DNK	Y / N / Partially / DNK
26.	Amoxicillin<br>Morning dose	Dose	Syrup [ ][ ].[ ][ ] mL<br>Distab [ ][ ][ ] mg	Y / N / Partially / DNK	Y / N / Partially / DNK	Y / N / Partially / DNK	Y / N / Partially / DNK	Y / N / Partially / DNK	Y / N / Partially / DNK	Y / N / Partially / DNK	Y / N / Partially / DNK	Y / N / Partially / DNK
27.	Amoxicillin<br>Evening dose	Dose	Syrup [ ][ ].[ ][ ] mL<br>Distab [ ][ ][ ] mg	Given/ Partially Given/ Not given/ do not kno	Given/ Partially Given/ Not given/ do not know	Given/ Partially Given/ Not given/ do not know	Given/ Partially Given/ Not given/ do not know	Given/ Partially Given/ Not given/ do not know	Given/ Partially Given/ Not given/ do not know	Given/ Partially Given/ Not given/ do not know	Given/ Partially Given/ Not given/ do not know	Given/ Partially Given/ Not given/ do not know
28.	For how many days did the infant receive A. Inj. Gentamicin [ ][ ] B. Inj. Ampicillin [ ][ ] C. Syrup Amoxicillin [ ][ ] (write N/A if not applicable)											

29.	How many complete doses did the infant receive	A. Inj. Gentamicin ☐☐	B. Inj. Ampicillin ☐☐	C. Syrup Amoxicillin ☐☐	(write N/A if not applicable)

	Day number	1	2	3	4	5	6	7		
**ADVERSE EVENTS (Y = Yes ; N= No; DNK= Do not Know)**										
30.	Diarrhea	Y/ N/ DNK	Y/ N/ DNK	Y/ N/ DNK	Y/ N/ DNK	Y/ N/ DNK	Y/ N/ DNK	Y/ N/ DNK	Y/ N/ DNK	Y/ N/ DNK
31.	Reduced urine output	Y/ N/ DNK	Y/ N/ DNK	Y/ N/ DNK	Y/ N/ DNK	Y/ N/ DNK	Y/ N/ DNK	Y/ N/ DNK	Y/ N/ DNK	Y/ N/ DNK
32.	Injection site infection	Y/ N/ DNK	Y/ N/ DNK	Y/ N/ DNK	Y/ N/ DNK	Y/ N/ DNK	Y/ N/ DNK	Y/ N/ DNK	Y/ N/ DNK	Y/ N/ DNK
33.	Injection site abscess	Y/ N/ DNK	Y/ N/ DNK	Y/ N/ DNK	Y/ N/ DNK	Y/ N/ DNK	Y/ N/ DNK	Y/ N/ DNK	Y/ N/ DNK	Y/ N/ DNK
34.	Any other	Y/ N/ DNK	Y/ N/ DNK	Y/ N/ DNK	Y/ N/ DNK	Y/ N/ DNK	Y/ N/ DNK	Y/ N/ DNK	Y/ N/ DNK	Y/ N/ DNK
35.	(If Other, specify)									

**OUTCOME OF THIS VISIT**										
36.	What is infants' condition since previous assessment? (Health workers assessment)	Same / Better / Worsened / Cured / Died / Don't know	Same / Better / Worsened / Cured / Died / Don't know	Same / Better / Worsened / Cured / Died / Don't know	Same / Better / Worsened / Cured / Died / Don't know	Same / Better / Worsened / Cured / Died / Don't know	Same / Better / Worsened / Cured / Died / Don't know	Same / Better / Worsened / Cured / Died / Don't know	Same / Better / Worsened / Cured / Died / Don't know	Same / Better / Worsened / Cured / Died / Don't know
37.	**ACTION TAKEN BY HEALTH WORKER**	Treatment continuing; / Referred /patient left / Cured/ Died	Treatment continuing / Referred /patient left / Cured/ Died	Treatment continuing; / Referred /patient left / Cured/ Died	Treatment continuing; / Referred /patient left / Cured/ Died	Treatment continuing; / Referred /patient left / Cured/ Died	Treatment continuing; / Referred /patient left / Cured/ Died	Treatment continuing; / Referred /patient left / Cured/ Died	Treatment continuing; / Referred /patient left / Cured/ Died	Treatment continuing; / Referred /patient left / Cured/ Died
**38.**	**If referred to higher facility, Name and address of the facility**									

39.	Form completed by	☐☐ (Initial of name only)	Date *(dd/mm/yyyy)*	☐☐/☐☐/☐☐☐☐
40.	Form checked by	☐☐ (Initial of name only)	Date *(dd/mm/yyyy)*	☐☐/☐☐/☐☐☐☐
41.	Form entered by	☐☐ (Initial of name only)	Date *(dd/mm/yyyy)*	☐☐/☐☐/☐☐☐☐
42.	Form entered by	☐☐ (Initial of name only)	Date *(dd/mm/yyyy)*	☐☐/☐☐/☐☐☐☐
