# Supplementary material for: Management of possible serious bacterial infection in young infants closer to home when referral is not feasible: Lessons from implementation research in Himachal Pradesh, India
Source: PLoS One. 2020 Dec 22;15(12):e0243724. doi: 10.1371/journal.pone.0243724 (PMC7755274; doi:10.1371/journal.pone.0243724)
Supplement: S5 Form — (PDF) [file pone.0243724.s005.pdf]

Country ID:	State ID:	Form ID:

Sick Newborn Home Visit **5**

Implementation Research on Management of Possible Serious Bacterial Infection (PSBI) In Young Infants (0-59 Days) Where Referral Is Not Feasible

A. Infant Identification

1. CHC	2. PHC	3. SC
4. Village	5. Infant ID	6. ASHA

B. First contact (Fill Q. 7 to Q. 18)

Sl. no.	Questions	Response	Skip / Remark
7.	First contact Date *(dd/mm/yyyy)*	☐☐/☐☐/☐☐☐☐	
8.	Status of the infant at the time of first contact (one answer only)	Infant is available……………………01	
		Infant died …………………………..02	
		Infant is hospitalized……………....03	
		Refused to give information…… 04	
		Other ……………………………….88<br><br>(Specify)	
9.	Adult primary respondent (First contact)	Father………………………………01	
		Mother……………………………..02	
		Other ……………………………….88<br><br>(Specify)	
10.	When did the child fall sick? (Date of onset – current illness episode)	☐☐/☐☐/☐☐☐☐	
11.	Who noticed/ told you that the child was sick?	Mother noticed……………………..01	
		Other family member……………..02	
		ASHA……………………………….03	
		ANM………………………………..04	
		Other……………………………….88<br><br>(Specify)	

Sl. no.	Questions	Response	Skip / Remark
12.	Who suggested you the place for treatment first?	Self/Family decision ……………..01	
		ANM……………………………….02	
		ASHA………………………………03	
		RMP……………………………….04	
		Other ………………………………88<br><br>(Specify)	
13.	Initially, where did you take your child for treatment?	Government Hospital…………….01	
		CHC……………………………….02	
		PHC………………………………..03	
		Sub-centre /ANM…………………04	
		Private Hospital/Clinic……………05	Name
		RMP………………………………..06	Name
		Other ……………………………88<br>(specify)	
14.	When did the treatment start?	☐☐/☐☐/☐☐☐☐	(except home remedies)
15.	Is your child under treatment today?	Yes …………………………………01	**Skip to Section D**
		No…………………………………..02	
16.	If no, why you stopped treatment?	Child died…………………………..01	
		No improvement…………………..02	
		Distance to facility was issue……03	
		No one to accompany…………….04	
		Child is fine now…………………..05	
		Treatment was costly…………….06	
		Other………………………………88<br><br>(Specify)	

B. First contact (Contd…..)

Sl. no.	Questions	Response	Skip / Remark
17.	Is the current place of treatment same where treatment started first?	Yes ………………………..01	Skip to Section D
		No…………………………..02	
		Not applicable ……………66 (if infant died)	Skip to section D
18.	If no, why have you changed the place?	Referred by provider…….01	
		No improvement …………02	
		Distance was issue ……...03	
		No one to accompany…..04	
		Treatment was costly…….06	
		Other……………………….88 (Specify)	

(In case of first contact with family, field worker will go to section D directly after filling question no 18)

C. Second Contact with Family *(Please fill this section during second contact)*

Sl. no.	Questions	Response	Skips
19.	Second contact Date *(dd/mm/yyyy)*	☐☐/☐☐/☐☐☐☐	
20.	Status of the infant on first contact (one answer only)	Infant is available………………….01	
		Infant died ………………………..02	
		Infant is hospitalized……………..03	
		Refused to give information…… 04	
		Other ………………………………88 (Specify)	
21.	Adult primary respondent (second contact)	Father……………………………..01	
		Mother……………………………..02	
		Other ………………………………88 (Specify)	

Country ID:	State ID:	Form ID:

Sick Newborn Home Visit **5**

D. Details of Care Seeking and Follow up by Health Staff at Home *(Add additional sheet in case of treatment more than 8 days)*

		Day 1	Day 2	Day3	Day 4	Day 5	Day 6	Day 7	Day 8
22.	Date DD/MM/YY	☐☐☐☐☐☐	☐☐☐☐☐☐	☐☐☐☐☐☐	☐☐☐☐☐☐	☐☐☐☐☐☐	☐☐☐☐☐☐	☐☐☐☐☐☐	☐☐☐☐☐☐
23.	How your child was treated?<br>(Tick one)	Inpatient ☐<br>Out patient ☐<br>At Home ☐	Inpatient ☐<br>Out patient ☐<br>At home ☐	Inpatient ☐<br>Out patient ☐<br>At home ☐	Inpatient ☐<br>Out patient ☐<br>At home ☐	Inpatient ☐<br>Out patient ☐<br>At home ☐	Inpatient ☐<br>Out patient ☐<br>At home ☐	Inpatient ☐<br>Out patient ☐<br>At home ☐	Inpatient ☐<br>Out patient ☐<br>At home ☐
24.	Who assessed your child health on that day?<br>(Tick)	Doctor ☐<br>Nurse ☐<br>ANM ☐<br>RMP ☐<br>Other ☐	Doctor ☐<br>Nurse ☐<br>ANM ☐<br>RMP ☐<br>No One ☐<br>Other ☐	Doctor ☐<br>Nurse ☐<br>ANM ☐<br>RMP ☐<br>No One ☐<br>Other ☐	Doctor ☐<br>Nurse ☐<br>ANM ☐<br>RMP ☐<br>No One ☐<br>Other ☐	Doctor ☐<br>Nurse ☐<br>ANM ☐<br>RMP ☐<br>No One ☐<br>Other ☐	Doctor ☐<br>Nurse ☐<br>ANM ☐<br>RMP ☐<br>No One ☐<br>Other ☐	Doctor ☐<br>Nurse ☐<br>ANM ☐<br>RMP ☐<br>No One ☐<br>Other ☐	Doctor ☐<br>Nurse ☐<br>ANM ☐<br>RMP ☐<br>No One ☐<br>Other ☐
		If other , Specify	If other , Specify	If other , Specify	If other , Specify	If other , Specify	If other , Specify	If other , Specify	If other , Specify
25.	Did your infant given injection?<br>(Tick one)	Yes ☐<br>No ☐<br>Do not know ☐	Yes ☐<br>No ☐<br>Do not know ☐	Yes ☐<br>No ☐<br>Do not know ☐	Yes ☐<br>No ☐<br>Do not know ☐	Yes ☐<br>No ☐<br>Do not know ☐	Yes ☐<br>No ☐<br>Do not know ☐	Yes ☐<br>No ☐<br>Do not know ☐	Yes ☐<br>No ☐<br>Do not know ☐
26.	Where did child receive treatment?<br>(INJECTION)<br>(Tick one)	DH ☐<br>CHC ☐<br>PHC ☐<br>Sub Centre ☐<br>Pvt Hospital ☐<br>RMP Clinic ☐<br>Don't Know ☐<br>Other ☐	DH ☐<br>CHC ☐<br>PHC ☐<br>Sub Centre ☐<br>Pvt Hospital ☐<br>RMP Clinic ☐<br>Don't Know ☐<br>Other ☐	DH ☐<br>CHC ☐<br>PHC ☐<br>Sub Centre ☐<br>Pvt Hospital ☐<br>RMP Clinic ☐<br>Don't Know ☐<br>Other ☐	DH ☐<br>CHC ☐<br>PHC ☐<br>Sub Centre ☐<br>Pvt Hospital ☐<br>RMP Clinic ☐<br>Don't Know ☐<br>Other ☐	DH ☐<br>CHC ☐<br>PHC ☐<br>Sub Centre ☐<br>Pvt Hospital ☐<br>RMP Clinic ☐<br>Don't Know ☐<br>Other ☐	DH ☐<br>CHC ☐<br>PHC ☐<br>Sub Centre ☐<br>Pvt Hospital ☐<br>RMP Clinic ☐<br>Don't Know ☐<br>Other ☐	DH ☐<br>CHC ☐<br>PHC ☐<br>Sub Centre ☐<br>Pvt Hospital ☐<br>RMP Clinic ☐<br>Don't Know ☐<br>Other ☐	DH ☐<br>CHC ☐<br>PH ☐<br>Sub Centre ☐<br>Pvt Hospital ☐<br>RMP Clinic ☐<br>Don't Know ☐<br>Other ☐
		If other , Specify	If other , Specify	If other , Specify	If other , Specify	If other , Specify	If other , Specify	If other , Specify	If other , Specify

Country ID:	State ID:	Form ID:	Sick Newborn Home Visit	5

		Day 1	Day 2	Day3	Day 4	Day 5	Day 6	Day 7	Day 8
27.	Who administered the injection? (Tick one)	Doctor ☐<br>Nurse / ANM ☐<br>RMP ☐<br>Don't Know ☐<br>Other ☐	Doctor ☐<br>Nurse / ANM ☐<br>RMP ☐<br>Don't know ☐<br>Other ☐	Doctor ☐<br>Nurse / ANM ☐<br>RMP ☐<br>Don't know ☐<br>Other ☐	Doctor ☐<br>Nurse / ANM ☐<br>RMP ☐<br>Don't know ☐<br>Other ☐	Doctor ☐<br>Nurse / ANM ☐<br>RMP ☐<br>Don't know ☐<br>Other ☐	Doctor ☐<br>Nurse / ANM ☐<br>RMP ☐<br>Don't Know ☐<br>Other ☐	Doctor ☐<br>Nurse / ANM ☐<br>RMP ☐<br>Don't Know ☐<br>Other ☐	Doctor ☐<br>Nurse / ANM ☐<br>RMP ☐<br>Don't know ☐<br>Other ☐
		If other , Specify	If other , Specify	If other , Specify	If other , Specify	If other , Specify	If other , Specify	If other , Specify	If other , Specify
28.	Was your child given any syrup (Tick one)	Yes ☐<br>No ☐<br>Do not know ☐	Yes ☐<br>No ☐<br>Do not know ☐	Yes ☐<br>No ☐<br>Do not know ☐	Yes ☐<br>No ☐<br>Do not know ☐	Yes ☐<br>No ☐<br>Do not know ☐	Yes ☐<br>No ☐<br>Do not know ☐	Yes ☐<br>No ☐<br>Do not know ☐	Yes ☐<br>No ☐<br>Do not know ☐
29.	Where was your child given syrup? (Tick)	At Facility ☐<br>At home ☐	At Facility ☐<br>At home ☐	At Facility ☐<br>At home ☐	At Facility ☐<br>At home ☐	At Facility ☐<br>At home ☐	At Facility ☐<br>At home ☐	At Facility ☐<br>At home ☐	At Facility ☐<br>At home ☐
30.	Did any health worker visit home to observe condition of child (Tick)	ANM ☐<br>ASHA ☐<br>No one ☐<br>Other ☐	ANM ☐<br>ASHA ☐<br>No one ☐<br>Other ☐	ANM ☐<br>ASHA ☐<br>No one ☐<br>Other ☐	ANM ☐<br>ASHA ☐<br>No one ☐<br>Other ☐	ANM ☐<br>ASHA ☐<br>No one ☐<br>Other ☐	ANM ☐<br>ASHA ☐<br>No one ☐<br>Other ☐	ANM ☐<br>ASHA ☐<br>No one ☐<br>Other ☐	ANM ☐<br>ASHA ☐<br>No one ☐<br>Other ☐
		If other , Specify	If other , Specify	If other , Specify	If other , Specify	If other , Specify	If other , Specify	If other , Specify	If other , Specify
31.	What was the condition of your child compared to last day? (Perception) (Tick one)		Same ☐<br>Better ☐<br>Worsen ☐<br>Cured ☐<br>Died ☐<br>Don't Know ☐	Same ☐<br>Better ☐<br>Worsen ☐<br>Cured ☐<br>Died ☐<br>Don't Know ☐	Same ☐<br>Better ☐<br>Worsen ☐<br>Cured ☐<br>Died ☐<br>Don't Know ☐	Same ☐<br>Better ☐<br>Worsen ☐<br>Cured ☐<br>Died ☐<br>Don't Know ☐	Same ☐<br>Better ☐<br>Worsen ☐<br>Cured ☐<br>Died ☐<br>Don't Know ☐	Same ☐<br>Better ☐<br>Worsen ☐<br>Cured ☐<br>Died ☐<br>Don't Know ☐	Same ☐<br>Better ☐<br>Worsen ☐<br>Cured ☐<br>Died ☐<br>Don't Know ☐

E. Serious Adverse Event (As reported by mother/caregiver)

		Day 1	Day 2	Day3	Day 4	Day 5	Day 6	Day 7	Day 8
32.	Did the infant has diarrhoea? (passed ≥3 stools which is looser than normal or watery stools)	Yes ☐<br>NO ☐	Yes ☐<br>NO ☐	Yes ☐<br>NO ☐	Yes ☐<br>NO ☐	Yes ☐<br>NO ☐	Yes ☐<br>NO ☐	Yes ☐<br>NO ☐	Yes ☐<br>NO ☐
33.	Did the infant has any skin rash (SKIN RASH)	Yes ☐<br>NO ☐	Yes ☐<br>NO ☐	Yes ☐<br>NO ☐	Yes ☐<br>NO ☐	Yes ☐<br>NO ☐	Yes ☐<br>NO ☐	Yes ☐<br>NO ☐	Yes ☐<br>NO ☐
34.	Did the infant not pass urine for 24 hours?	Yes ☐<br>NO ☐	Yes ☐<br>NO ☐	Yes ☐<br>NO ☐	Yes ☐<br>NO ☐	Yes ☐<br>NO ☐	Yes ☐<br>NO ☐	Yes ☐<br>NO ☐	Yes ☐<br>NO ☐
35.	Did the infant pass urine lesser than normal? (Freq./ quantity)	Yes ☐<br>NO ☐<br>Not sure ☐	Yes ☐<br>NO ☐<br>Not sure ☐	Yes ☐<br>NO ☐<br>Not sure ☐	Yes ☐<br>NO ☐<br>Not sure ☐	Yes ☐<br>NO ☐<br>Not sure ☐	Yes ☐<br>NO ☐<br>Not sure ☐	Yes ☐<br>NO ☐<br>Not sure ☐	Yes ☐<br>NO ☐<br>Not sure ☐
36.	Did the infant has swellings with redness /puss at injection site?	Yes ☐<br>NO ☐	Yes ☐<br>NO ☐	Yes ☐<br>NO ☐	Yes ☐<br>NO ☐	Yes ☐<br>NO ☐	Yes ☐<br>NO ☐	Yes ☐<br>NO ☐	Yes ☐<br>NO ☐
37.	In your knowledge, was any change introduced in treatment by the treatment provider?	Stopped Med ☐<br>Changed Med ☐<br>Reduced dose ☐<br>Change Freq. ☐<br>No change ☐<br>Not Sure ☐	Stopped Med ☐<br>Changed Med ☐<br>Reduced dose ☐<br>Change Freq. ☐<br>No change ☐<br>Not Sure ☐	Stopped Med ☐<br>Changed Med ☐<br>Reduced dose ☐<br>Change Freq. ☐<br>No change ☐<br>Not Sure ☐	Stopped Med ☐<br>Changed Med ☐<br>Reduced dose ☐<br>Change Freq. ☐<br>No change ☐<br>Not Sure ☐	Stopped Med ☐<br>Changed Med ☐<br>Reduced dose ☐<br>Change Freq. ☐<br>No change ☐<br>Not Sure ☐	Stopped Med ☐<br>Changed Med ☐<br>Reduced dose ☐<br>Change Freq. ☐<br>No change ☐<br>Not Sure ☐	Stopped Med ☐<br>Changed Med ☐<br>Reduced dose ☐<br>Change Freq. ☐<br>No change ☐<br>Not Sure ☐	Stopped Med ☐<br>Changed Med ☐<br>Reduced dose ☐<br>Change Freq. ☐<br>No change ☐<br>Not Sure ☐

Country ID:	State ID:	Form ID:

F. Illness Narrative

38.	Give a brief narrative account of the onset of illness, care seeking, issues faced, treatment received (Please put dates and experience as described by mother)

39.	Form completed by	☐☐ (Initial of name only)	Date *(dd/mm/yyyy)*	☐☐/☐☐/☐☐☐☐
40.	Form checked by	☐☐ (Initial of name only)	Date *(dd/mm/yyyy)*	☐☐/☐☐/☐☐☐☐
41.	Form entered by	☐☐ (Initial of name only)	Date *(dd/mm/yyyy)*	☐☐/☐☐/☐☐☐☐
42.	Form entered by	☐☐ (Initial of name only)	Date *(dd/mm/yyyy)*	☐☐/☐☐/☐☐☐☐
