# Supplementary material for: Management of possible serious bacterial infection in young infants closer to home when referral is not feasible: Lessons from implementation research in Himachal Pradesh, India
Source: PLoS One. 2020 Dec 22;15(12):e0243724. doi: 10.1371/journal.pone.0243724 (PMC7755274; doi:10.1371/journal.pone.0243724)
Supplement: S6 Form — (PDF) [file pone.0243724.s006.pdf]

**Implementation Research on management of Possible Serious Bacterial Infection in young infants where referral is not feasible through strengthening of existing Home Based new born care program of the Government of India.**

**Health Facility Form - Community Health Centre (CHC)**

| | |
|---|---|
| 1. Block Name: | 2. District Name: |
| 3. Health Facility Name: | 4. Time (in hours): [ ] [ ] |
| 5. Name of Medical Officer in Charge | 6. Signature of Medical Officer in Charge/doctor |
| 7. Name of the Person taking Audit information: | 8. Date of Audit (dd/mm/yy): [ ] [ ]/ [ ] [ ]/ [ ] [ ] |

| **Summary Score** | **Good** | **To be improved** | | | |
|---|---|---|---|---|---|
| **(To be circled)** | **5** | **4** | **3** | **2** | **1** |
| **Please indicate the quality of support by marking one of the five numbers; 5 indicates good quality of care, 4 to 1 indicating levels of necessary improvement (4= small need for improvement, 1= urgent need for improvement.)** | | | | | |

**1. Basic Information and Training of Healthcare Providers**

| | | |
|---|---|---|
| 1 | Number of PHCs & additional PHCs under this CHC | [ ] [ ] |
| 2 | Number of doctors in this CHC | [ ] [ ] |
| 3 | Total number of doctors who attend children (age 1-59 days) | [ ] [ ] |
| 4 | How many of these doctors have been given training on F-IMNCI/IMNCI guidelines **within last 36 months**? | [ ] [ ] |
| 5 | How many of these doctors have been trained on F- IMNCI/IMNCI guidelines **before 36 months**? | [ ] [ ] |
| 6 | How many of these doctors have **never** been trained on F-IMNCI/IMNCI guidelines? | [ ] [ ] |
| 7 | Number of Staff Nurses posted | [ ] [ ] |
| 8 | Number of ASHA posted | [ ] [ ] |
| 9 | Number of ASHA that report | [ ] [ ] |
| 10 | Have ASHAs of your area been trained on HBNC Module 6? | [ ] Yes , all [ ] None [ ] training ongoing |
| 11 | Have ASHAs of your area been trained on HBNC Module 7? | [ ] Yes , all [ ] None [ ] training ongoing |
| 12 | Number of ANMs posted | [ ] [ ] |

| | | |
|---|---|---|
| 13 | How many of these ANMs have been trained for administration of Inj. Ampicillin /Inj. Gentamycin in young infants | [ ] [ ] |

| **Summary Score – Training of Healthcare Providers** | | | | | |
|---|---|---|---|---|---|
| **(To be circled)** | **5** | **4** | **3** | **2** | **1** |

## 2. Information of patients attended, admitted and referred in health facility

| | | |
|---|---|---|
| **Data for Today** | | |
| 1. | No. of young infants (aged 1 – 59 days) attended today at this facility (8 a.m. to 2 p.m.) | [ ][ ][ ] |
| 2. | No. of young infants (aged 1 – 59 days) admitted today, at the centre (8 a.m. to 2 p.m.) | [ ][ ][ ] |
| 3. | No. of young infants (aged 1 – 59 days) referred today, to a higher centre (8 a.m. to 2 p.m.) | [ ][ ][ ] |
| **Data for Last 3 months** | | |
| 4. | No. of young infants (aged 1 – 59 days) attended at the centre in last 3 months | [ ][ ][ ] |
| 5. | No. of young infants (aged 1 – 59 days) admitted in last 3 months | [ ][ ][ ] |
| 6. | No. of young infants (aged 1 – 59 days) referred at higher centre in last 3 months | [ ][ ][ ] |
| 7. | How many of these accepted referrals? | [ ][ ][ ] |
| **Data for PSBI Last 3 months** | | |
| 8. | No. of young infants (aged 1 – 59 days) with **PSBI**attended at the centre in last 3 months | [ ][ ][ ] |
| 9. | No. of young infants (aged 1 – 59 days) with **PSBI**admitted in last 3 months. | [ ][ ][ ] |
| 10. | No. of young infants (aged 1 – 59 days) with **PSBI**referred at higher centre in last 3 months | [ ][ ][ ] |
| 11. | How many of these accepted referrals? | [ ][ ][ ] |
| 12. | How many of these refused referrals? | [ ][ ][ ] |

## 3. Observation of ANC related activities at the Health Facility

| | | |
|---|---|---|
| 1. | Is an ANC counselling going on in this CHC/PHC today? | 1. Yes<br>2. No (go to next section) [ ] |
| 2. | Who is providing counselling | Doctor [ ]<br>Staff Nurse [ ]<br>ANM [ ]<br>LHV/BSW [ ] |
| 3. | How many mothers are attending ANC session today? | |
| 4. | Which of these mothers have 2nd visit of ANC today? | |
| 5. | How many of these mothers are primiparous? | |
| 6. | How many of these mothers are not critically ill? | |

<table>
<tr><td colspan="4">Exit interview of ANC mother:<br>This interview has to be conducted with a mother who is having 2nd ANC visit today at this CHC/PHC. She should not be suffering from any medical illness as it will jeopardise the interview. Preference should be given to a mother who is primiparous.</td></tr>
<tr><td>7.</td><td>Did you get counselling on new born care during this ANC Visit?</td><td colspan="2">1. Yes<br>2. No (go to Q.9) [ ]</td></tr>
<tr><td>8.</td><td>What information do you know about/was given to you on new born care? (Tick all responses that are applicable)<br>1. Cord Care<br>2. Eye Care<br>3. Breast Feeding<br>4. Wrapping<br>5. Skin to skin contact<br>6. Bathing<br>7. Routine Immunization<br>8. 102/108 Ambulance Service</td><td>Spontaneous Answer<br>[ ]<br>[ ]<br>[ ]<br>[ ]<br>[ ]<br>[ ]<br>[ ]<br>[ ]</td><td>Prompted Answer<br>[ ]<br>[ ]<br>[ ]<br>[ ]<br>[ ]<br>[ ]<br>[ ]<br>[ ]</td></tr>
<tr><td>9.</td><td>Were you informed about any of the danger signs in new born during the Antenatal Visits?</td><td colspan="2">1. Yes<br>2. No [ ]</td></tr>
<tr><td>10.</td><td>Which danger signs in new born are you aware of/you were told about?<br>(Tick all responses that are applicable)<br>a. Not able to feed since birth/stopped feeding well or not feeding at all<br>b. Convulsions/ Seizures<br>c. Lower Chest Movements (Severe Chest Indrawing)<br>d. Hot to touch<br>e. Feels cold to touch<br>f. Movement only when stimulated<br>g. Fast Breathing (breaths 60/minute of more)<br>h. Jaundice<br>i. Diarrhoea<br>j. Pustules (10 or more) or one large focus of infection<br>k. Grunting</td><td>Spontaneous Answer<br>[ ]<br>[ ]<br>[ ]<br>[ ]<br>[ ]<br>[ ]<br>[ ]<br>[ ]<br>[ ]<br>[ ]<br>[ ]</td><td>Prompted Answer<br>[ ]<br>[ ]<br>[ ]<br>[ ]<br>[ ]<br>[ ]<br>[ ]<br>[ ]<br>[ ]<br>[ ]<br>[ ]</td></tr>
</table>

## 4. Observation of PNC related activities at the Health Facility

| | | |
|---|---|---|
| **1.** | Is there a mother currently staying in health facility who has delivered within last 24 hours?<br>(Note: If there are more than one deliveries in last 24 hours select mother who is younger in age.) | 1. Yes<br>2. No (go to next section) [ ] |
| **2.** | Did you get counselling on new born care during PNC? | 1. Yes [ ]<br>2. No |
| **3.** | What information was given to you on new born care? *(Tick all responses that are applicable)*<br>1. Cord Care<br>2. Eye Care<br>3. Breast Feeding<br>4. Wrapping | [ ]<br>[ ]<br>[ ]<br>[ ] |

| | | |
|---|---|---|
| | 5. Skin to skin contact<br>6. Bathing<br>7. Routine Immunization<br>8. 102/108 Ambulance Service | [ ]<br>[ ]<br>[ ] |
| **4.** | Were you informed about any of the danger signs in new born during the Antenatal Visits? | 1. Yes [ ]<br>2. No |
| **5.** | Which danger signs in new born were you told about?<br>*(Tick all responses that are applicable)*<br>a. Not able to feed since birth/stopped feeding well or not feeding at all<br>b. Convulsions/ Seizures<br>c. Lower Chest Movements (Severe Chest Indrawing)<br>d. Hot to touch<br>e. Feels cold to touch<br>f. Movement only when stimulated<br>g. Fast Breathing (breaths 60/minute of more)<br>h. Jaundice<br>i. Diarrhoea<br>j. Pustules (10 or more) or one large focus of infection<br>k. Grunting | [ ]<br>[ ]<br>[ ]<br>[ ]<br>[ ]<br>[ ]<br>[ ]<br>[ ]<br>[ ]<br>[ ]<br>[ ]<br>[ ] |
| **6.** | Who gave you this information? | ANM [ ]<br>Doctor [ ]<br>Staff Nurse [ ] |

## 5. Manpower

| S.No. | Personnel | IPHS Norm | Govt. Norm | Available at CHC | Remarks/Gaps Identified |
|---|---|---|---|---|---|
| **5.1** | **Clinical Manpower** | | | | |
| A | General Surgeon | **1** | **0** | [ ] | |
| B | Physician | **1** | **0** | [ ] | |
| C | Obstetrician / Gynaecologist | **1** | **0** | [ ] | |
| D | Paediatrician | **1** | **0** | [ ] | |
| E | Anaesthetist | **1** | **0** | [ ] | |
| F | Public Health Program Manager | **1** | **0** | [ ] | |
| G | Eye Surgeon | **1** | **0** | [ ] | |
| H | General duty officers | | **2** | [ ] | |

| | | | | | |
|---|---|---|---|---|---|
| | (Medical Officer) | | | | |
| I | Other specialists (if any) | | | [ ] | |
| **5.2** | **Support Manpower** | | | | |
| A | Nursing Staff | **7+2** | **1** | [ ] | **(1 ANM and 1 Public Health Nurse for family welfare will be appointed under ASHA scheme)** |
| B | Dresser | **1** | **0** | [ ] | |
| C | Pharmacist / compounder | **1** | **1** | [ ] | |
| D | Lab. Technician | **1** | **1** | [ ] | |
| E | Radiographer | **1** | **0** | [ ] | |
| F | Ophthalmic Assistant | **1** | **0** | [ ] | |
| G | Ward boys / nursing orderly | **2** | **1** | [ ] | |
| H | Sweepers | **3** | **1** | [ ] | |
| I | Chowkidar | **1** | **0** | [ ] | |
| J | OPD Attendant | **1** | **0** | [ ] | |
| K | Statistical Assistant / Data entry operator | **1** | **0** | [ ] | |
| L | OT Attendant | **1** | **0** | [ ] | |
| M | Registration Clerk | **1** | **1** | [ ] | |
| N | Driver | | **1** | [ ] | |
| O | Any other staff (specify) | | | [ ] | |

| **Summary Score - Manpower** | | | | | |
|---|---|---|---|---|---|
| **(To be circled)** | **5** | **4** | **3** | **2** | **1** |

## 6. Physical Infrastructure

| S. No | | Status at CHC<br>(1. Yes/ 2. No) | Remarks/Suggestions |
|---|---|---|---|

| | | | |
|---|---|---|---|
| **6.1** | **Location** | | |
| 6.1.1 | Within Village Locality | [ ] | |
| 6.1.2 | Far from village locality (in km) | [ ] | |
| 6.1.3 | Whether located at less than 2 hours of travel distance from the farthest village? (Yes/No) | [ ] | |
| 6.1.4 | Whether the district head quarter hospital located at a distance of less than 4 hours travel time? (Yes/No) | [ ] | |
| 6.1.5 | Is the facility easily accessible? (Yes/No) | [ ] | If no, provide details: |
| 6.1.6 | Is CHC accessible by all-weather road? (Yes/No) | [ ] | |
| **6.2** | **Building** | | |
| 6.2.1 | Is a designated government building available for the CHC? (Yes / No) | [ ] | |
| A | If there is no designated government building, then where is the CHC located? (Rented building, other govt. building, etc.) | | |
| 6.2.2 | Area of the building (Total area in Sq. meters.) | | |
| 6.2.3 | What is the present stage of construction of the building? (1. Complete/2. Incomplete) | [ ] | |
| 6.2.4 | Compound Wall / Fencing (1-All around; 2-Partial; 3-None) | [ ] | |
| 6.2.5 | Condition of plaster on walls (1- Well plastered with plaster intact everywhere; 2- Plaster coming off in some places; 3- Plaster coming off in many places or no plaster) | [ ] | |
| 6.2.6 | Condition of floor (1- Floor in good condition; 2- Floor coming off in some places; 3- Floor coming off in many places or no proper flooring) | [ ] | |
| 6.2.7 | Whether the cleanliness is 1. Good /2. Fair /3. Poor? | [ ] | |
| 6.2.8 | Pharmacy for drug dispensing and drug storage (Yes/No) | [ ] | |
| 6.2.9 | Separate public utilities for males and females (Yes/No) | [ ] | |
| A | Are the toilets clean? | [ ] | |
| B | Patients have access to clean running water, soap, etc for washing hands? | [ ] | |
| 6.2.10 | Adequate no. of windows in the room for light and air in each room (Yes/No) | [ ] | |

| | | | |
|---|---|---|---|
| 6.2.11 | OPD rooms / cubicles (Yes/No) (Give numbers) | [ ] | |
| 6.2.12 | Waiting room for patients (Yes/No) | [ ] | |
| 6.2.13 | Emergency Room / Casualty (Yes/No) | [ ] | |
| 6.2.14 | **Wards:** | | |
| 1. | Beds are clean, well maintained and have mattresses. | [ ] | |
| 2. | Patients have access to clean bed linen | [ ] | |
| 3. | Ward is kept clean | [ ] | |
| 4. | Separate wards for males and females (Yes/No) | [ ] | |
| A | No. of beds: Male | [ ] | |
| B | No. of beds: Female | [ ] | |
| 5. | Separate wards for neonates, infants and children (Yes/No) | [ ] | |
| A | No of beds available | [ ] | |
| B | Special area for KMC available (Yes/No) | [ ] | |
| C | Sick newborns are kept in separate room form healthy babies? (Yes/No) | [ ] | |
| D | Is there an emergency management area in or near each ward? (Yes/No) | [ ] | |
| 6.2.15 | Operation Theatre available and in use? (Yes/No) | [ ] | |
| A | If OT is available and not in use, what are the reasons for it? | | |
| B | Does OT have enough space (Yes / No) (At least 240 sq meters) | [ ] | |
| C | Is OT fitted with air conditioner? (Yes / No) | [ ] | |
| D | Is generator available for OT? (Yes / No) | [ ] | |
| E | Is emergency light available in OT? (Yes / No) | [ ] | |
| F | Is fumigation done regularly? (Yes / No) | [ ] | |
| 6.2.16 | Labour room available? (Yes/ No) | [ ] | |
| A | If labour room is present, are deliveries carried out in the labour room? (Yes/ No) | [ ] | |
| B | If labour room is present. but deliveries are not being conducted there, then what are the reasons for the same? | | |

| | | | |
|---|---|---|---|
| C | How many deliveries are carried out in a day? (Average) | [ ] | |
| D | Who is the care provider conducting deliveries? | [ ] | |
| E | Is a new born corner available? (Yes/No) | | |
| 6.2.17 | X-ray room with dark room facility (Yes/No) | [ ] | |
| 6.2.18 | Laboratory (Yes/No) | [ ] | |
| 6.2.19 | Cold Chain | | |
| A | Walk-in coolers (Yes / No) | [ ] | |
| B | Walk-in freezers available (Yes / No) | [ ] | |
| C | Ice lined freezers (Yes / No) | [ ] | |
| D | Deep freezers (Yes / No) | [ ] | |
| E | Refrigerators (Yes / No) | [ ] | |
| 6.2.20 | Blood Storage Unit | | |
| A | Is the CHC having linkage with district blood bank? (Yes / No) | [ ] | |
| B | Is regular blood supply available? (Yes / No) | [ ] | |
| 6.2.21 | Ancillary Rooms - Nurses rest room (Yes/No) | [ ] | |
| 6.2.22 | **Source of water** (1- Piped; 2- Bore well/ hand pump / tube well; 3- Well; 4- Other (specify)) | [ ] | |
| 6.2.23 | **Type of sewerage system** (1- Soak pit; 2- Connected to Municipal Sewerage) | [ ] | |
| 6.2.24 | Office room (Yes/No) | [ ] | |
| 6.2.25 | Store room (Yes/No) | [ ] | |
| 6.2.26 | Kitchen (Yes / No) | [ ] | |
| A | Diet provided by hospital? (Yes/No) | [ ] | |
| B | Is pantry available for babies? (Yes/No) | [ ] | |
| 6.2.27 | Residential facility for the staff with living condition available? (Yes/No) | [ ] | |
| 6.2.28 | Accommodation facility for families of admitted patients available? (Yes/No) | [ ] | |
| **6.3** | **Waste disposal** | | |
| 6.3.1 | Is there an incinerator? (Yes / No) | [ ] | |

| | | | |
|---|---|---|---|
| 6.3.2 | If yes, type (1- electric; 2- Other (specify) | [ ] | |
| 6.3.3 | If no, how the medical waste disposed? | | |
| **6.4** | **Electricity** | | |
| 6.4.1 | Is there electric line in all parts of the hospital? (1- In all parts; 2- In some parts; 3- None) | [ ] | |
| 6.4.2 | Regular Power Supply (1- Continuous Power Supply; 2- Occasional power failure; 3- Power cuts in summer only; 4- Regular power cuts; 5- No power supply | [ ] | |
| 6.4.3 | Stand by facility (generator) available (Yes / No) | [ ] | |
| **6.5** | **Are heating facilities available throughout? (If yes, specify what type)** | [ ] | |
| **6.6** | **Communication facilities available?** (1=Telephone; 2=Personal Computer; 3= NIC Terminal; 4= Email) | [ ] | |
| **6.7** | **Vehicles Available (Ambulance, Jeep, Car)** | [ ] | |
| **6.8** | **Security personnel available? (Yes/No)** | [ ] | |

| **Summary Score - Infrastructure** | | | | | |
|---|---|---|---|---|---|
| **(To be circled)** | **5** | **4** | **3** | **2** | **1** |

**7. Investigative Facilities**

| **S.No.** | **IPHS Norm** | **Current Availability at CHC (1. Yes) (2. No)** | **Remarks / Suggestions / Identified Gaps** |
|---|---|---|---|
| 1 | Availability of ECG facilities | [ ] | |
| 2 | X-Ray facility | [ ] | |
| 3 | Ultrasound facility | [ ] | |
| 4 | Appropriate training to a nursing staff on ECG | [ ] | |
| 5 | Lab test facilities | [ ] | |
| 6 | Any lab test / diagnostic test outsourced to private lab / hospital (please specify the test) | [ ] | |
| 7 | All necessary reagents, glassware and facilities for collection and transportation of samples | [ ] | |

| **Summary Score – Investigative facilities** | | | | | |
|---|---|---|---|---|---|
| **(To be** | **5** | **4** | **3** | **2** | **1** |

| **circled)** | | | | | |
|---|---|---|---|---|---|

**8. Laboratory tests**

| Lab Test | Availability during day<br><br>(Yes/No) | Availability during night<br><br>(Yes/No) | Comments/Remarks |
|---|---|---|---|
| Blood Glucose | [ ] | [ ] | |
| Haemoglobin | [ ] | [ ] | |
| Haematocrit (PCV) | [ ] | [ ] | |
| Leukocyte count | [ ] | [ ] | |
| Blood gas analysis | [ ] | [ ] | |
| Blood grouping and crossmatch | [ ] | [ ] | |
| Bilirubin | [ ] | [ ] | |
| Rhesus antibodies | [ ] | [ ] | |
| Urine microscopy | [ ] | [ ] | |
| Urine dipstick | [ ] | [ ] | |
| Bacteriology (Culture) | [ ] | [ ] | |
| Bacteriology (Smear) | [ ] | [ ] | |
| Full blood count | [ ] | [ ] | |
| Coagulation tests | [ ] | [ ] | |
| Liver function tests | [ ] | [ ] | |
| Renal function tests | [ ] | [ ] | |
| Electrolytes | [ ] | [ ] | |
| HIV test | [ ] | [ ] | |
| Coombs' test: direct and indirect | [ ] | [ ] | |
| Serum protein and Albumin | [ ] | [ ] | |
| CD4 count | [ ] | [ ] | |
| Rapid test for syphilis | [ ] | [ ] | |
| Rapid diagnostic test or | [ ] | [ ] | |

| | | | |
|---|---|---|---|
| microscopy for Malaria | | | |
| CSF Microscopy | [ ] | [ ] | |
| Immature to total neutrophil ratio | [ ] | [ ] | |

| **Summary Score - Laboratory** | | | | | |
|---|---|---|---|---|---|
| **(To be circled)** | **5** | **4** | **3** | **2** | **1** |

### 9. Essential equipment and supplies

| **Name** | **Availability** | **In working/usable condition** | **Name** | **Availability** | **In working/usable condition** |
|---|---|---|---|---|---|
| Syringes 5 ml (0.2 ml markings) | Yes [ ] No [ ] | Yes [ ] No [ ] | Endo-tracheal tube | Yes [ ] No [ ] | Yes [ ] No [ ] |
| Syringes 2 ml (0.1 ml markings) | Yes [ ] No [ ] | Yes [ ] No [ ] | Thermometer | Yes [ ] No [ ] | Yes [ ] No [ ] |
| Syringes 1 ml (0.1 ml markings) | Yes [ ] No [ ] | Yes [ ] No [ ] | Ambu-bag | Yes [ ] No [ ] | Yes [ ] No [ ] |
| Needles: 23-25 Gauge; Upto 2.5cm in length | Yes [ ] No [ ] | Yes [ ] No [ ] | Paediatric digital weighing scale | Yes [ ] No [ ] | Yes [ ] No [ ] |
| Naso-gastric tubes | Yes [ ] No [ ] | Yes [ ] No [ ] | Pulse Oximeter | Yes [ ] No [ ] | Yes [ ] No [ ] |
| Distilled water for injection | Yes [ ] No [ ] | Yes [ ] No [ ] | Straight Blade Laryngoscope | Yes [ ] No [ ] | Yes [ ] No [ ] |
| Anti-septic swabs | Yes [ ] No [ ] | Yes [ ] No [ ] | Minute Timer | Yes [ ] No [ ] | Yes [ ] No [ ] |
| IV sets | Yes [ ] No [ ] | Yes [ ] No [ ] | Infusion pump | Yes [ ] No [ ] | Yes [ ] No [ ] |
| Photo-therapy units | Yes [ ] No [ ] | Yes [ ] No [ ] | Baby Warmer | Yes [ ] No [ ] | Yes [ ] No [ ] |
| Clean water | Yes [ ] No [ ] | Yes [ ] No [ ] | Litre containers for mixing ORS solution | Yes [ ] No [ ] | Yes [ ] No [ ] |
| Cups and spoons for giving ORS | Yes [ ] No [ ] | Yes [ ] No [ ] | Containers for sending ORS with referred infant | Yes [ ] No [ ] | Yes [ ] No [ ] |
| Heating lamp for neonates | Yes [ ] No [ ] | Yes [ ] No [ ] | Measuring board for height/length | Yes [ ] No [ ] | Yes [ ] No [ ] |
| Oxygen source: Oxygen cylinder/concent | Yes [ ] No [ ] | Yes [ ] No [ ] | Sphygmomanometer | Yes [ ] No [ ] | Yes [ ] No [ ] |

| | | | | | |
|---|---|---|---|---|---|
| rator/central supply | | | | | |
| Bags and masks (adult and neonatal size) | Yes [ ] No [ ] | Yes [ ] No [ ] | Cord cutting/clamping set | Yes [ ] No [ ] | Yes [ ] No [ ] |
| Sterile gloves (various sizes) | Yes [ ] No [ ] | Yes [ ] No [ ] | Incubator | Yes [ ] No [ ] | Yes [ ] No [ ] |
| Sterile gauze | Yes [ ] No [ ] | Yes [ ] No [ ] | Suction catheter | Yes [ ] No [ ] | Yes [ ] No [ ] |
| Breast pumps | Yes [ ] No [ ] | Yes [ ] No [ ] | Double hook infusion stand | Yes [ ] No [ ] | Yes [ ] No [ ] |
| Glucometers | Yes [ ] No [ ] | Yes [ ] No [ ] | Flow-meters for oxygen | Yes [ ] No [ ] | Yes [ ] No [ ] |
| Syringe hub cutter | Yes [ ] No [ ] | Yes [ ] No [ ] | Gowns for staff and mother | Yes [ ] No [ ] | Yes [ ] No [ ] |
| Electric sterilizer | Yes [ ] No [ ] | Yes [ ] No [ ] | Autoclave | Yes [ ] No [ ] | Yes [ ] No [ ] |
| Radiant warmer | Yes [ ] No [ ] | Yes [ ] No [ ] | Mobile X-ray | Yes [ ] No [ ] | Yes [ ] No [ ] |
| Suturing set and material | Yes [ ] No [ ] | Yes [ ] No [ ] | Bilirubinometer | Yes [ ] No [ ] | Yes [ ] No [ ] |
| Is all the equipment safe? | | | | Yes | No |
| Is all the equipment cleaned regularly and sanitary? | | | | Yes | No |
| Is all the equipment kept in good working order? | | | | Yes | No |

| **Summary Score –Essential equipment and supplies** | | | | | |
|---|---|---|---|---|---|
| **(To be circled)** | **5** | **4** | **3** | **2** | **1** |

## 10. Essential Drugs and Medicines

| **Name** | **Availability** | **Quantity** | **Name** | **Availability** | **Quantity** |
|---|---|---|---|---|---|
| Inj. Ampicillin | Yes [ ] No [ ] | | Oral Antipyretics | Yes [ ] No [ ] | |
| Inj. Gentamycin | Yes [ ] No [ ] | | Oral Co-trimoxazole | Yes [ ] No [ ] | |
| Amoxicillin | Yes [ ] No [ ] | | Ceftriaxone | Yes [ ] No [ ] | |
| Gentian Violet (0.5%) | Yes [ ] No [ ] | | Benzylpenicillin | Yes [ ] No [ ] | |
| Gentian Violet (0.25%) | Yes [ ] No [ ] | | Sugar | Yes [ ] No [ ] | |
| Ringer lactate IV | Yes [ ] No [ ] | | ORS packets | Yes [ ] No [ ] | |
| Chlorhexidine | Yes [ ] No [ ] | | Vaccines for: BCG, | Yes [ ] No [ ] | |

| | | | | | |
|---|---|---|---|---|---|
| Gluconate (4%) | | | HepB, OPV, DPT+HIB+HepB, rotavirus | | |
| Oxygen Cylinder | Yes [ ] No [ ] | | Normal saline IV | Yes [ ] No [ ] | |
| Glucose (5%, 10%, 30-50%) IV | Yes [ ] No [ ] | | Paracetamol Tab | Yes [ ] No [ ] | |
| Ciprofloxacin | Yes [ ] No [ ] | | Chloramphenicol | Yes [ ] No [ ] | |
| Vitamin K IM inj. | Yes [ ] No [ ] | | Phenobarbital | Yes [ ] No [ ] | |
| Furosemide IV | Yes [ ] No [ ] | | Diazepam | Yes [ ] No [ ] | |
| Corticosteroids (Oral or IV) | Yes [ ] No [ ] | | Antistaphylococcal penicillin | Yes [ ] No [ ] | |
| All anti malaria drugs needed according to national malaria control programe. | Yes [ ] No [ ] | | Any other: Specify<br>1:<br>2:<br>3:<br>4: | | |
| **How long does it take between medicine requisition request and stock delivery?** | | | | | |
| **In the past 6 months has there been continuous supply and availability of drugs? (Yes/No)** | | | | [ ] | |
| **When is the request for restocking sent?** | | | | | |
| Monthly basis | | | | [ ] | |
| At low stock levels | | | | [ ] | |
| **From where are the medicines procured from?** | | | | | |

| **Summary Score – Essential Drugs and Medicines** | | | | | |
|---|---|---|---|---|---|
| **(To be circled)** | **5** | **4** | **3** | **2** | **1** |

**11. Medical Records**

| Standards and Criteria | Yes/No | Score (1-5) | Comments |
|---|---|---|---|
| Are all the records clear and legible? | | | |
| Are records dated? | | | |
| Are all admission and discharge diagnoses clearly written in notes? | | | |
| Are all drugs and treatments clearly identifiable? | | | |
| Is information from previous admissions available to staff providing care to mothers, neonates and children? | | | |
| Is information from antenatal records available to staff providing care during labour? | | | |
| Do mothers have access to their medical records? | | | |

| | | | |
|---|---|---|---|
| Do parents have access to their child's medical records? | | | |

| Summary Score – Medical Records | | | | | |
|---|---|---|---|---|---|
| **(To be circled)** | **5** | **4** | **3** | **2** | **1** |
