# Supplementary material for: Management of possible serious bacterial infection in young infants closer to home when referral is not feasible: Lessons from implementation research in Himachal Pradesh, India
Source: PLoS One. 2020 Dec 22;15(12):e0243724. doi: 10.1371/journal.pone.0243724 (PMC7755274; doi:10.1371/journal.pone.0243724)
Supplement: S7 Form — (PDF) [file pone.0243724.s007.pdf]

Health Facility Survey: Sub Centre

1. Identification

State			
District			
Block			
Name of Sub center			
Date of Assessment	Day:	Month:	Year:
Name and signature of person collecting data			

2. Services

S.No.		
**2.1**	**Population covered (in numbers)**	
**2.2**	**MCH Care including Family Planning**	
**2.2.1**	**Service availability**	**(Yes$_1$ / No$_2$)**
A	Ante-natal care	[ ]
B	Intranatal care	[ ]
C	Post-natal care	[ ]
D	New born Care	[ ]
E	Child care including immunization	[ ]
F	Family Planning and contraception	[ ]
G	Adolescent health care	[ ]
H	Assistance to school health services	[ ]
I	Facilities under Janani Suraksha Yojana	[ ]
J	Treatment of minor ailments	[ ]
K	First aid (specify)	[ ]
**2.2.2**	**Availability of specific services**	**(Yes$_1$ / No$_2$ / Not Applicable$_9$)**
A	Does the doctor visit the Sub center at least once in a month?	[ ]
B	Is the day and time of this visit fixed?	[ ]
C	Are the residents of the village aware of the timings of the doctor's visit?	[ ]
D	Does the Health Assistant (male) or LHV visit the Sub Centre at least once a week?	[ ]
E	Is the Antenatal care (Inj. T.T, IFA tablets, weight and BP checkup) provided by those in the Sub center?	[ ]
F	Is the facility for referral of complicated cases of pregnancy / delivery available at Sub center for 24 hours?	[ ]
G	Does the ANM/any trained personnel accompany the woman in labor to the referred care facility at the time of referral?	[ ]
H	Are the Immunization services as per Government schedule provided by the Sub center	[ ]
I	Is the ORS for prevention of diarrhea and dehydration available in the Subcenter?	[ ]
J	Is the treatment of minor illness like fever, cough, cold, worm disinfestation etc. available in the Sub center	[ ]
K	Are the contraceptive services like insertion of Copper-T, distributing Oral contraceptive pills or condoms provided by the Sub center?	[ ]
**2.3**	**Monitoring and Supervision activities**	**(Yes$_1$ / No$_2$)**
A	Training of traditional birth attendants and ASHA	[ ]

B	Monitoring of Water quality in the village	[ ]
C	Coordination and supervision of activities of ASHA	[ ]
D	Proper maintenance of records and registers	[ ]
E	Watch over unusual health events	[ ]
F	Coordinated services with AWWs, ASHA, Village Health and Sanitation Committee, PRIs	[ ]

3. Manpower

S.No.	Personnel	Existing	Recommended	Current Availability at Sub Centre (Indicate Numbers)	Remarks / Suggestions / Identified Gaps
3.1.	Health Worker (Female)	1	1 or 2 (Optional)		
3.2.	Health Worker (Male)	1	1 or 0 (optional; may be replaced by female health worker)		
3.3.	Voluntary worker to keep the Sub Centre clean and assisting ANM. She is paid by the ANM from her contingency fund @ Rs. 100 per month	1 (optional)	1 (optional)		

4. Physical Infrastructure

S.No.		Availability at Subcenter	Remarks
**4.1**	**Location**		
A	Where is the subcenter located?		
(i)	Within Village Locality		
(ii)	Far from village locality		
B	Is the subcenter easily accessible? (Yes_1/No_2)		
C	The distance of Sub Centre (in Km) from the nearest village in the coverage area		
D	Travel time to reach the Sub Centre from the nearest place in the coverage area		
E	The distance of Sub Centre (in Km) from the furthest village in the coverage area		
F	Travel time to reach the Sub Centre from the furthest place in the coverage area		
G	The distance of Sub Centre (in Km) from the PHC		
H	The distance of Sub Centre (in Km) from the CHC		
**4.2**	**Building**		
A	Is a designated government building available for the Sub Centre? (Yes_1 /		

	No_2)		
B	Area of the building (Total area in Sq. meters.)		
C	What is the present stage of construction of the building		
(i)	Construction complete		
(ii)	Construction incomplete		
D	Compound Wall / Fencing<br>1.All around<br>2.Partial<br>3.None		
E	Condition of floor<br>1. Floor in good condition<br>2. Floor coming off in some places<br>3. Floor coming off in many places or no proper flooring)		
F	Cleanliness<br>Good / Fair / Poor? (Observe)		
G	Separate public utilities for males and females (Yes_1/No_2)		
H	Clinic Room		
I	Examination room		
J	Water supply		
(i)	Source of water<br>1. Piped<br>2. Bore well/ hand pump / tube well<br>3. Well<br>4.Other (specify))		
(ii)	Whether overhead tank and pump exist (Yes_1 / No_2)		
K	Regular electric supply available? (Yes_1 / No_2)		
L	Transport facility for movement of staff (Yes_1 / No_2)		
M	Residential facility for the staff		

5. Equipment

S.No.	Equipment	Available at Sub center (Yes/No)	Remarks
1	Weighing scale		
2	Radiant warmer		
3	Head circumference measurement tape		
4	Respiratory rate timer		
5	Thermometer		
6	Gloves (various sizes)		
7	Disposable bags and masks		

6. Drugs

S.No.	Drug	Available	Remarks
1	Paracetamol Tablets/Syrup		
2	Iron Folic Acid tab		
3	Gentian violet		

4	Oral rehydration solution		
5	Ampicillin tab		
6	Zinc Sulphate Dispersible tab		
7	Gentamycin injection		
8	Amoxicillin tab		
9	Sodium chloride (normal saline)		
10	Vitamin A Suspension		
11	B-complex Tablet		

7. Records/Registers

S.No.	Name	Complete/Incomplete	Remarks
1.			
2.			
3.			
4.			
5.			

8. Additional remarks:
