# Supplementary material for: Management of possible serious bacterial infection in young infants closer to home when referral is not feasible: Lessons from implementation research in Himachal Pradesh, India
Source: PLoS One. 2020 Dec 22;15(12):e0243724. doi: 10.1371/journal.pone.0243724 (PMC7755274; doi:10.1371/journal.pone.0243724)
Supplement: S8 Form — (PDF) [file pone.0243724.s008.pdf]

Health Facility Survey: Primary Health Centre

1. Identification

State:			
District:			
Block:			
Name of PHC:			
Date of Assessment:	Day:	Month:	Year:
Name and signature of person collecting data:			

2. Services

S.No.			
**2.1**	**Population covered (In numbers)**		
**2.2**	**Assured services available (Yes_1/No_2)**	**General**	**Infant <2 months**
A	OPD services	[ ]	[ ]
B	Number of beds available	[ ]	[ ]
C	In-patient services	[ ]	[ ]
D	Emergency services (24hours)	[ ]	[ ]
**2.3**	**OPD Attendance during last one month**		
2.3.1	Male (Total)	[ ]	
2.3.2	Female (Total)	[ ]	
2.3.3	Infants	[ ]	
A	Age <2 months	[ ]	
B	Age 2 months - 1year	[ ]	
**2.4**	**MCH Care including Family Planning**		
2.4.1	Service availability (Yes_1 / No_2)		
A	Antenatal care	[ ]	
B	Intranatal care (24 - hour delivery services both normal and assisted)	[ ]	
C	Post-natal care	[ ]	
D	New born Care	[ ]	
E	Child care including immunization	[ ]	
F	Family Planning	[ ]	
G	MTP	[ ]	
H	Management of RTI / STI	[ ]	
2.4.2	**Availability of specific services**		
A	Are antenatal clinics organized by the PHC regularly? (Yes_1 / No_2)	[ ]	
B	Are the low birth weight babies managed at the PHC? (Yes_1 / No_2)	[ ]	
C	Is there a fixed immunization day?(Yes_1 / No_2)	[ ]	
D	How is the vaccine received at PHC and distributed to Sub Centers?		
E	Is the treatment of children with pneumonia available at the PHC?(Yes_1 / No_2)	[ ]	

F	Is the management of children suffering from diarrhoea with severe dehydration done at the PHC?<br>(Yes$_1$ / No$_2$)	[ ]
G	Is telemedicine facility available?(Yes$_1$ / No$_2$)	[ ]
(i)	Timing of the service	[ ]
(ii)	Frequency of use in last three months	[ ]
H	Is there a service for identification of sick infant with PSBI?<br>(Yes$_1$ / No$_2$)	[ ]
I	Is simplified regime available and provided to patients with PSBI?(Yes$_1$ / No$_2$)	[ ]
**2.5**	**Monitoring and Supervision activities (Yes$_1$ / No$_2$)**	
2.5.1	Monitoring and supervision of activities of sub-centers through regular meetings / periodic visits, etc.	[ ]
2.5.2	Monitoring of National Health Programs	[ ]
2.5.3	Monitoring activities of ASHAs	[ ]
2.5.4	Visits of Medical Officer to all sub-centers at least once in a month	[ ]
2.5.5	Visits of Health Assistants (Male) and LHV to sub-centers once a week	[ ]

3. Physical Infrastructure

S.No.	Infrastructure	Status in PHC	Remarks/Gaps identified
**3.1**	**Location of PHC**		
3.1.1	Within village		
3.1.2	Far from village (specify distance in km)		
3.1.3	Located in easily accessible area? (Yes/No)		
3.1.3	Distance of PHC (in km) from the farthest village in coverage area.		
3.1.4	Travel time (in minutes) to reach the PHC from farthest village in coverage area.		
3.1.5	Distance of PHC (in km) from the nearest village in coverage area.		
3.1.6	Travel time (in minutes) to reach the PHC from nearest village in coverage area.		
3.1.7	Distance of PHC (in km) from the CHC.		
3.1.8	Distance of PHC (in km) from District Hospital.		
**3.2**	**Building**		
3.2.1	Designated Government building (Yes$_1$ / No$_2$)	[ ]	
3.2.2	Other:<br>(Rented premises, other govt. building - specify.)		
3.2.3	State of construction<br>1. Complete<br>2. Incomplete	[ ]	
3.2.4	Area of the building (sq. m)		
3.2.5	Condition of plaster on walls:<br>1. Well plastered with plaster intact everywhere<br>2. Plaster coming off in some places<br>3. Plaster coming off in many places or no plaster	[ ]	
3.2.6	Condition of floor:<br>1. Floor in good condition<br>2. Floor coming off in some places	[ ]	

	3. Floor coming off in many places or no proper flooring		
3.2.7	Cleanliness (Good$_1$/Fair$_2$/Poor$_3$)		
A	OPD	[ ]	
B	Ward	[ ]	
C	Rooms	[ ]	
D	Toilets	[ ]	
3.2.8	Residence quarters for MO/other staff available? (Yes/No)	[ ]	
3.2.9	Pharmacy for drug dispensing and drug storage (Yes/No)	[ ]	
3.2.10	Waiting room for patients (Yes/No)	[ ]	
3.2.11	Emergency Room / Casualty (Yes/No)	[ ]	
3.2.12	Laboratory (Yes/No)	[ ]	
3.2.13	Labour room available? (Yes/ No)	[ ]	
A	If labour room is present, are deliveries carried out in the labour room?	[ ]	
B	If labour room is present but deliveries are not being conducted there, then what are the reasons for the same?		
3.2.14	Separate wards for males and females (Yes/No)	[ ]	
A	No. of beds (male)	[ ]	
B	No. of beds (female)	[ ]	
C	Specific ward for children	[ ]	
D	New born corner	[ ]	
3.2.15	Separate public utilities for males and females (Yes/No)	[ ]	
3.2.16	Adequate no. of windows in the room for light and air in each room (Yes/No)	[ ]	
3.2.17	Vehicle (ambulance/other vehicle) available? (Yes / No)	[ ]	
**3.3**	**Electricity**		
3.3.1	Is there electric line in all parts of the PHC?<br>1. In all parts<br>2. In some parts<br>3. None	[ ]	
3.3.2	Regular Power Supply<br>1. Continuous Power Supply<br>2. Occasional power failure<br>3. Power cuts in summer only<br>4. Regular power cuts<br>5. No power supply	[ ]	
3.3.3	Stand by facility (generator) available in working condition (Yes / No)	[ ]	
**3.4**	**Water Supply**<br>1. Piped<br>2. Bore well/ hand pump/tube well<br>3. Well<br>4. Other (specify)	[ ]	

4. Manpower

S.No.	Personnel	Current Availability at PHC (Yes_1 / No_2)	Working Hours and Days	Remarks / Suggestions / Identified Gaps (Provide duration of availability)
1	Medical Officer	[ ]		
2	Pharmacist	[ ]		
3	Nurse - Midwife (Staff Nurse)	[ ]		
4	Health Worker (Female)	[ ]		
5	Health Educator	[ ]		
6	Health Assistant (One male and One female)	[ ]		
7	Clerks	[ ]		
8	Laboratory Technician	[ ]		
9	Driver	[ ]		
10	Class IV	[ ]		
**Total**		[ ]		

5. Training of personnel during previous (full) year

5.1.	Available training for	Training provided (With duration)	Remarks
a.	Health Worker (Female)		
b.	Health Worker (Male)		
c.	Medical Officer		
d.	Initial and periodic training of paramedics in treatment of minor ailments		
e.	Training of ASHAs		
f.	Periodic training of Doctors through Continuing Medical Education, conferences, skill development training etc. on emergency obstetric care		
g.	Training of Health Workers in antenatal care and skilled birth attendance		

6. Essential Equipment

Sr. No.	Equipment	Available	Functional	Remarks / Suggestions / Identified Gaps
1	Weighing scale			
2	Baby warmer/incubator			
3	Head circumference measurement tape			
4	Respiratory rate timer			
5	Thermometer			
6	Gloves (various sizes)			
7	Disposable bags and masks			
8	Normal Delivery Kit			
9	Equipment for assisted vacuum delivery			
10	Equipment for assisted forceps delivery			
11	Standard Surgical Set (for minor procedures like episiotomies stitching)			
12	Equipment for Manual Vacuum Aspiration			
13	Equipment for New Born Care and Neonatal Resuscitation			
14	IUD insertion kit			
15	ILR/Deep Freezer			
16	Ice box			
17	Radiant warmer for new born baby			
18	Phototherapy unit			
19	Feeding tubes for baby			
20	Self-inflating bag and mask-neonatal size			
21	Equipment for Eye care and vision testing: Tonometers (Schiotz), direct opthalmoscope, illuminated vision testing drum, trial lens sets with trial frames, snellen and near vision charts			

7. Drugs

S.No.	Drug	Availability	Remarks
**1.**	**In the past 6 months has there been continuous supply and availability of drugs?**		
**2.**	**How long does it take between medicine requisition request and stock delivery?**		
**3.**	**When is the request for restocking sent?**		
A	Monthly basis		
B	At low stock levels		
**4.**	**From where are the medicines procured from?**		
**5.**	**Essential Drugs Required:**		
A	Paracetamol Tablets/Syrup		
B	Oral rehydration solution		
C	Ampicillin tab		
D	Zinc Sulphate Dispersible tab		
E	Gentamycin injection		
F	Sodium chloride (normal saline)		
G	Albendazole		
H	Aluminum Hydroxide		
I	Amoxicillin		
J	Calamine Lotion		
K	Calcium Carbonate		

L	Chlorhexidine		
M	Chlorpheniramine		
N	Chloramphenicol		
O	Diazepam – Rectal suppository		
P	Dicyclomine		
Q	Domperidone		
R	Ferrous Sulfate + Folic Acid		
S	Furazolidone		
T	Gentian Violet 1% solution		
U	Metronidazole		
V	Miconazole 1% cream		
W	Primaquine		
X	Sodium Bicarbonate		
Y	Vitamin A liquid		
Z	Vitamin B complex		

8. **Essential Laboratory Services**

Sr. No.	Service	Availability (Yes/No)	Comments/Remarks
1	Routine urine, stool and blood tests		
2	Blood grouping		
3	Bleeding time, clotting time		
4	Diagnosis of RTI/STDs with wet mounting, grams stain etc.		
5	Sputum testing for TB		
6	Blood smear examination for malaria parasite		
7	Rapid tests for pregnancy		
8	Rapid tests for HIV		
9	Others (specify)		

9. **Records/Registers Maintained**

S.No.	Name	Complete/Incomplete	Remarks
1.			
2.			
3.			
4.			
5.			

10. **Additional remarks**
