# Supplementary material for: Management of possible serious bacterial infection in young infants closer to home when referral is not feasible: Lessons from implementation research in Himachal Pradesh, India
Source: PLoS One. 2020 Dec 22;15(12):e0243724. doi: 10.1371/journal.pone.0243724 (PMC7755274; doi:10.1371/journal.pone.0243724)
Supplement: S9 Form — (PDF) [file pone.0243724.s009.pdf]

# Health Facility Assessment

## Care of Neonates and Young Infants


### Abstract

[Draw your reader in with an engaging abstract. It is typically a short summary of the document. When you’re ready to add your content, just click here and start typing.]



SAS

[Email address]

| **1.** | **Is health facility able to provide routine care to neonates immediately after birth?** |
|---|---|
| 1.1 | Does the health facility has written up-to-date clinical protocol for essential newborn care that are consistent with WHO guidelines and are available in the labor and childbirth areas of the maternity unit? |
| Ask | |
| Confirm | Look for printed copy of the protocol |
| 1.2 | Does the health facility has supplies of the following items available in the sufficient qualities at all the tomes for the expected number of births? |
| Confirm | Sterile cord ties/Cord clamps: ________________________________________<br><br>Sterile scissors/blade: ______________________________________________ |
| 1.3 | Does the health facility has supplies of clean towels in the labor and childbirth areas for immediate drying of the newborns, available in sufficient quantity at all times for the expected number of births? |
| Confirm | Minimum of two clean sterile towels for each birth |
| 1.4 | Have the healthcare staff in the labor and childbirth areas of the maternity unit received in-service training or regular refresher sessions in essential newborn care and breastfeeding support at least once during last 12 months? |
| Ask | Last event: Where, when, by whom, who participated |
| Confirm | Look for any documentary proof |
| 1.5 | Does the health facility has local arrangement to maintain a documented room temperature in the labor and childbirth areas at or above 25$^{o}$ C and free of droughts? |
| Ask | For air conditioning, heaters<br>For documentation of temp |
| Confirm | For air conditioning, heater<br>Documented temperature of last 7 days<br>Presence of draught of air |
| 1.6 | Does the healthcare staff in the labor and childbirth areas receive at least monthly drills or simulation exercises and supportive supervision in essential newborn care and supporting breastfeeding? |
| Ask | Last event: Where, when, by whom, who participated |
| Confirm | Look for any documentary proof |

| | |
|---|---|
| 1.7 | What proportion of newborns are breastfed within 1 h of birth? |
| Ask | For information if available from the facility register or dashboard _____________ |
| Confirm | In postnatal ward ask all the available mothers at what time they gave first breastfeeding to their baby?<br>Total mother available? ________________<br>Answered your question? ______________<br>Breastfed within 1 h of birth? ____________ |
| 1.8 | What proportion of all newborns are kept in skin-to-skin contact with their mothers for at least 1 h after birth? |
| Ask | For information if available from the facility register or dashboard _____________ |
| Confirm | In postnatal ward ask all the available mothers about skin-to-skin contact?<br>Total mother available? ________________<br>Answered your question? ______________<br>Kept baby in skin to skin contact for 1 h ____________<br>If there is a delivery in your presence was this practice done? ________________ |
| 1.9 | What is the proportion of newborns whose umbilical cord was clamped 1-3 minutes after birth? |
| Ask | For information if available from the facility register or dashboard _____________ |
| Confirm | If there is a delivery in your presence was this practice done? ________________ |
| 1.10 | What proportion of all newborns are dried immediately and thoroughly at birth? |
| Ask | For information if available from the facility register or dashboard _____________ |
| Confirm | If there is a delivery in your presence was this practice done? ________________ |
| 1.11 | What proportion of all newborns who had a normal body temperature at the 1-2 h of birth? |
| Ask | How many newborns are present in postnatal ward? ________________<br>How many have documented temperature at 1-2 h of age? ________________<br>How many of these had normal body temperature? _________________ |
| Confirm | How many newborns are present in postnatal ward with age 1-2 h? ________________<br>Measure their axillary temperature. How many of these had normal body temperature? ________________ |
| 1.12 | What proportion of neonates receive all four elements of essential newborn care: immediate and thorough drying, immediate skin-to-skin contact, delayed cord clamping and initiation of breastfeeding in the first hour? |
| Ask | For information if available from the facility register or dashboard _____________ |
| Confirm | If there is a delivery in your presence were these practices followed? ________________ |

| 2. | **Is health facility able to provide routine postnatal care to neonates?** |
|---|---|
| 2.1 | Does the health facility has written up-to-date clinical protocol for postnatal care that are available in the postnatal care areas? |
| Ask | |
| Confirm | Look for printed copy of the protocol |
| 2.2 | Does the health facility practice and enables rooming in to allow mothers and newborns to remain together 24 h a day |
| Ask | |
| Confirm | Look in the postnatal areas. Especially look for neonates born by C-section. |
| 2.3 | Does the health facility has a written breast feeding policy and are healthcare professionals aware of it? |
| Ask | |
| Confirm | Look for printed copy of the policy<br><br>Ask nurses in postnatal ward about the policy |
| 2.4 | Have healthcare professionals working in the postnatal ward received in-service and regular refresher training in routine postnatal care and breastfeeding in last 12 months |
| Ask | Last event: Where, when, by whom, who participated |
| Confirm | Look for any documentary proof |
| 2.5 | Does the health facility has arrangements to ensure that mothers know when and where postnatal care to newborns will be provided after discharge (this may be by visiting this health facility or by home visit by a community health worker) |
| Ask | |
| Confirm | By asking mothers who are being discharged today |
| 2.6 | Does the health facility has arrangements for alternative feeding methods? |
| Ask | For availability of gavage, spoon/paladai/cup/katori.<br>Is bottle feeding also practiced? |
| Confirm | Look if these are available:<br>Feeding tubes size 5/6/7/8: ________________<br>Disposable syringes 5-20 ml: _______________<br>Spoon: ____________________<br>Katori: ________________<br>Paladai: _______________ |

| | |
|---|---|
| 2.7 | Does the health facility has arrangements to inform pregnant women about benefits and management of breastfeeding? |
| Ask | For such activity in the antenatal care areas in the form of educational videos/counselling/flip charts etc. |
| Confirm | Look for any evidence for such an activity |
| 2.8 | Does the health facility ensure that demonstration of formula feeding is done only when it is necessary and dangers of the same are explained to the parents while demonstrating? |
| Ask | |
| Confirm | Look for a mother who is using formula for feeding. Ask<br>Whether she was demonstrated how to mix and make formula feed ______<br>Whether she knows dangers of formula feed___________ |
| 2.9 | What proportion of newborns in the postnatal ward have received vitamin K, BCG, OPV and hepatitis B |
| Confirm | Total neonates admitted: _______________<br>Vitamin K admin documented: _____________<br>BCG admin documented: ____________<br>OPV admin documented: ____________<br>Hepatitis B admin documented: ____________ |
| 2.10 | What proportion of neonates are exclusively breastfed from birth till discharge? |
| Confirm | Number of neonates who are to be discharged today __________________<br>Number who have received only breastfeeding from birth to discharge ________________<br>If not available you can obtain phone number of 5 families in whom newborns are discharged in last 24-28 h. Phone and ask about this question. |
| 2.11 | What proportion of mothers have documented breastfeeding problems? |
| Conform | Number of postnatal mothers _______________<br>Number in which breastfeeding problem is documented _______________ |
| 2.12 | What proportion of newborns have complete physical examination before discharge? |
| Confirm | Number of neonates who are at least 24 h old or are to be discharged today __________________<br>Number who have documented complete physical examination ________________ |
| 2.13 | What proportion of newborns and their mothers are discharged at least 24 h after birth? |
| Confirm | Number of neonates who are to be discharged today __________________<br>Number who are at least 24 h of age________________<br>If not available you can obtain phone number of 5 families in whom newborns are discharged in last 24-28 h. Phone and ask about this question. |
| 2.14 | What proportion of neonates have in their patient record documented information on body temperature, respiratory rate, feeding and absence and presence of danger signs? |
| Confirm | Number of neonates admitted currently _________________<br>Number in which information about all the 4 parameters is documented ______________<br>What is frequency of documentation ________________ |
| 2.15 | What proportion of mothers in postnatal wards have received breastfeeding counselling and support? |
| Confirm | Number of mothers available _________________<br>Number who received counselling _______________ |

| | |
|---|---|
| 2.16 | What proportion of mothers are allowed rooming-in with their newborns 24 h a day |
| Confirm | Total number of postnatal mothers__________________<br>Ask in how many rooming in has been practiced over last 24 h/since birth in last 24 h ________________ |
| 2.17 | What proportion of newborns are being exclusively breastfed at the time of discharge? |
| Confirm | Number of neonates who are to be discharged today ___________________<br>Number who have received are on exclusive breastfeeding at the time of discharge _________________<br>If not available you can obtain phone number of 5 families in whom newborns are discharged in last 24-28 h. Phone and ask about this question. |

| 3. | **Can health facility manage as per current guidelines those neonates who are not breathing at the time of birth?** | | | |
|---|---|---|---|---|
| 3.1 | Does the health facility has written and up-to-date clinical protocol for managing neonates who are not breathing spontaneously in the childbirth areas of the hospital? | | | |
| Ask | | | | |
| Confirm | Look for printed copy of the protocol | | | |
| 3.2 | Does the health facility has following devices in the childbirth and neonatal areas of the hospital?<br>Look for separate devices in each area where birth takes place and where neonates are kept | | | |

| Confirm | | Birth area 1 | Birth area 2 | Postnatal ward | Neonatal area |
|---|---|---|---|---|---|
| Answer Y or N | Suction machine | | | | |
| | Suction catheters | | | | |
| | Oxygen source | | | | |
| | Preterm mask | | | | |
| | Term mask | | | | |
| | Self-inflating bag | | | | |

| | |
|---|---|
| 3.3 | Can all health-care workers who provide care for newborns in the health facility are skilled in basic newborn resuscitation, as demonstrated by simulating positive pressure ventilation with a bag-and-mask on a manikin |
| Assess | Total number of healthcare professional assessed: _____________________________<br>Number who can demonstrate PPV on a manikin: _____________________________ |
| 3.4 | Has health care staff in the childbirth and neonatal areas of the maternity unit received regular refresher sessions in basic newborn resuscitation at least once every 12 months? |
| Ask | Last event: Where, when, by whom, who participated |
| Confirm | Look for any documentary proof |
| 3.5 | Do health care staff in the childbirth and neonatal areas of the maternity unit receive monthly drills or simulation exercises and supportive supervision in basic newborn resuscitation? |
| Ask | Last event: Where, when, by whom, who participated |
| Confirm | Look for any documentary proof |
| 3.6 | What proportion of all newborns who were not breathing spontaneously after additional stimulation at the health facility were resuscitated with a bag-and-mask? |
| Ask | For instances of cases in which bag and mask could not be given |
| Confirm | Look for record documenting need of resuscitation. This may be available as a dashboard indicator of the facility |
| 3.7 | What is the proportion of all newborns who were not breathing spontaneously after additional stimulation |

| | |
|---|---|
| | and were resuscitated with a bag-and-mask within 1 min of birth. |
| Ask | For instances of cases in which bag and mask could not be given |
| Confirm | Look for record documenting need of resuscitation. This may be available as a dashboard indicator of the facility |
| 3.8 | What is the proportion of all live babies born at term (≥ 37 weeks) with no major congenital malformations in the health facility who died within 7 days of birth (early neonatal mortality) |
| Confirm | From record of last 3 months |
| 3.9 | What is the proportion of all live babies born at term (≥ 37 weeks) in the health facility who were not breathing spontaneously at birth but started breathing spontaneously 5 min after resuscitation. |
| Confirm | Look for record. This may be indicated by an Apgar score of less than 7 at 1 minute but 7 or more at 5 minutes of age |

| 4. | **Do women in preterm labour receive appropriate interventions for their babies, according to WHO guidelines?** |
|---|---|
| 4.1 | Does the health facility has written and up-to-date clinical protocol for administration of antenatal corticosteroids, antibiotics and magnesium sulfate in pregnant women likely to deliver prematurely? |
| Ask | |
| Confirm | Look for printed copy of the protocol |
| 4.2 | Does the health facility has supplies of antenatal corticosteroids (dexamethasone or betamethasone), antibiotics and magnesium sulfate available in sufficient quantities at all times to manage preterm birth in accordance with WHO guidelines? |
| Ask | Whether these three drugs are in the hospital pharmacy and whether there have been instances of not being available anytime in last 3 months<br>Dexamethasone or betamethasone: _________________<br>Antibiotics for mothers with PPROM as per policy: _________________________<br>Magnesium sulfate: _____________________________ |
| Confirm | Check that these items are available in the patient care areas (antenatal ward and labor room)<br>Dexamethasone or betamethasone: _________________<br>Antibiotics for mothers with PPROM as per policy: _________________________<br>Magnesium sulfate: _____________________________ |
| 4.3 | What is the proportion of all preterm newborns born between 24 and 34 weeks of gestation in the health facility whose mothers received at least one dose of antenatal corticosteroids when indicated? |
| Confirm | Ask for this record.<br>1. This may be available as a dashboard indicator. ________________If not available<br>2. Check from SNCU database. __________________If not available,<br>3. Check from record of all candidate mothers and neonates admitted in the hospital_________ |
| 4..4 | What is the proportion of all preterm newborns born before 32 weeks of gestation in the health facility whose mothers received magnesium sulfate to protect their baby from fetal neurological complications? |
| Confirm | Ask for this record.<br>1. This may be available as a dashboard indicator. ________________If not available<br>2. Check from SNCU database. __________________If not available,<br>3. Check from record of all candidate mothers and neonates admitted in the hospital_________ |
| 4.5 | What is the proportion of all women with preterm pre-labor rupture of membranes who gave birth in the health facility who received prophylactic antibiotics? |
| Confirm | Ask for this record.<br>1. This may be available as a dashboard indicator. ________________If not available<br>2. Check from SNCU database. __________________If not available,<br>3. Check from record of all candidate mothers and neonates admitted in the hospital_________ |

| | | | |
|---|---|---|---|
| **5.** | **Do preterm and small babies receive appropriate care, according to WHO guidelines?** | | |
| 5.1 | Does the health facility has written, up-to-date clinical protocols for the care of small and preterm babies in the childbirth/postnatal areas that are consistent with WHO guidelines? | | |
| Ask | | | |
| Confirm | Look for printed copy of the protocol | | |
| 5.2 | Does the health facility has supplies and materials to provide optimal thermal care to stable and unstable preterm babies, including kangaroo mother care (support binders, baby hats, socks), clean incubators and radiant warmers? | | |
| Confirm | Equipment/supplies | Total | Functional |
| | Incubators | | |
| | Radiant warmer | | |
| | KMC chairs | | |
| | Support binders/gowns/culturally appropriate tier for KMC | | |
| | Any other equipment used for thermal care: | | |
| | | | |
| | Total number of preterm or sick babies admitted at present in the health facility: | | |
| 5.3 | Does the health facility has the supplies and materials to provide optimal feeding to preterm babies and support for breastfeeding or alternative feeding (feeding cups and spoons, infant formula, breast pumps, milk-storage facilities, pasteurizers, milk banks if possible, nasogastric tubes, syringe drivers, intravenous fluids and tubing)? | | |
| Confirm | Equipment | Yes/No | |
| | Feeding cup/spoon and katori/paladai | | |
| | Boiler | | |
| | Infant formula | | |
| | Breast pump | | |
| | Refrigerator for milk storage | | |
| | Milk pasteurizer | | |
| | Milk bank | | |
| | Feeding tube | | |
| | Syringe pump | | |
| | IV fluid | | |
| | IV tubing | | |
| | IV cannula | | |
| 5.4 | Have the health care staff in the health facility who work with pregnant and postpartum women and newborns received in-service training and regular refresher sessions in appropriate care of preterm and low-birth-weight babies in last 12 months? | | |
| Ask | Last event: Where, when, by whom, who participated | | |
| Confirm | Look for any documentary proof | | |
| 5.5 | What proportion of all low-birth-weight newborns born in the health facility with a birth weight ≤ 2000 g have received near-continuous kangaroo mother care in the first week of life? | | |
| Ask | Look at dashboard indicator or health facility record if this information is available: ___________ | | |
| Confirm | Total number of ≤2000 g newborns admitted in the hospital at present: _____________<br>Number who received near-continuous KMC in the first week: _____________ | | |
| 5.6 | What proportion of all unstable low-birth-weight newborns weighing ≤ 2000 g who cannot receive kangaroo | | |

<table>
<tr><td></td><td colspan="3">mother care in the health facility are cared for in a thermo-neutral environment, either under radiant warmers or in incubators, as appropriate?</td></tr>
<tr><td>Confirm</td><td colspan="3">Total number of unstable ≤2000 g newborns who cannot receive KMC at present: ________<br>Of above, number under radiant warmer or incubator: ____________</td></tr>
<tr><td>5.7</td><td colspan="3">What is the proportion of all low-birth-weight newborns born in the health facility whose mothers have received additional support to establish breastfeeding?</td></tr>
<tr><td>Confirm</td><td colspan="3">Total LBW neonates at present: ______________<br>Number who have received support to establish breastfeeding: ____________<br>What is the support received? __________________</td></tr>
<tr><td>5.8</td><td colspan="3">What is the proportion of all preterm babies (< 28 weeks, 28–32 weeks and 32–37 weeks of gestational age) born in the health facility who died within the first 7 days of life?</td></tr>
<tr><td>Confirm</td><td colspan="3">Get this information from the facility register/dashboard</td></tr>
<tr><td rowspan="5"></td><td>Category</td><td>Number born in last 3 months</td><td>Number died within first 7 days</td></tr>
<tr><td>All preterm</td><td></td><td></td></tr>
<tr><td><28 weeks</td><td></td><td></td></tr>
<tr><td>28-32 weeks</td><td></td><td></td></tr>
<tr><td>32-37 weeks</td><td></td><td></td></tr>
<tr><td>5.9</td><td colspan="3">What is the proportion of all low-birth-weight newborns born in the health facility who were exclusively fed on their mother's milk during their stay in the health facility?</td></tr>
<tr><td>Ask</td><td colspan="3">Ask if this information is available from facility records/dashboard indicators</td></tr>
<tr><td>Confirm</td><td colspan="3">Total number of LBW neonates at present: _____________<br>Total number who have been exclusive breastfeeding till now: _________________</td></tr>
<tr><td>5.10</td><td colspan="3">What is proportion of all live preterm babies born in the health facility who had severe neonatal morbidity (respiratory distress syndrome, intraventricular haemorrhage, necrotizing enterocolitis).</td></tr>
<tr><td>Ask</td><td colspan="3">Get this information from the health facility records<br>Disease ____________________ n/N ______________________</td></tr>
<tr><td>Confirm</td><td colspan="3">Get this information from neonates currently admitted in the health facility<br>Disease ____________________ n/N ______________________</td></tr>
<tr><td>5.11</td><td colspan="3">What proportion of low-birth-weight baby deaths in the facility are attributed to possible serious bacterial infection or sepsis?</td></tr>
<tr><td>Ask</td><td colspan="3">Get this information from the health facility records</td></tr>
</table>

| 6. | **Do newborns with suspected infection or risk factors for infection are promptly given antibiotic treatment, according to WHO guidelines?** |
|---|---|
| 6.1 | Does the health facility has a written, up-to-date clinical protocol for early diagnosis and management of neonatal infection in the childbirth areas that is consistent with WHO guidelines? |
| Ask | |
| Confirm | Look for printed copy of the protocol |
| 6.2 | Does the health care staff in the health facility who care for newborns infants have received in-service training and refresher sessions in the recognition and management of suspected newborn infections during last 12 months? |
| Ask | Last event: Where, when, by whom, who participated |
| Confirm | Look for any documentary proof |
| 6.3 | Does the health facility has supplies of injectable antibiotics (at least first- and second-line antibiotics for neonatal sepsis and meningitis) available in sufficient quantities at all times for the expected case load? |
| Ask | Ask and list antibiotics which are available and the number health facility gets every month<br>Antibiotic Number/month |
| Confirm | Check the stock for of antibiotics which are available and the number available<br>Antibiotic Number now available |
| 6.4 | Does the health care staff in the health facility know the signs of newborn sepsis and how to treat it, according to WHO guidelines? |
| Confirm | Check this by administering the MCQ tool: ________________________ |
| 6.5 | What proportion of all newborns in the health facility with signs of infection are receiving injectable antibiotics |
| Confirm | Total newborns with suspected signs of infection at present: ___________<br>Number getting "injectable" antibiotics: ____________ |
| 6.6 | What proportion of all newborns of mothers with signs of infection in the health facility are receiving injectable antibiotics? |
| Confirm | Total newborns with suspected signs of infection in mother at present*: ___________<br>Number getting "injectable" antibiotics: ____________<br><br>*If mother has any of the following: PPROM, fever, chorioamnionitis, foul-smelling amniotic fluid, UTI or is dai handled |
| 6.7 | What is the proportion of newborns treated for sepsis in the health facility who died (case fatality rate) |
| Confirm | Get this number from the hospital records/dashboard indicators |

| | |
|---|---|
| 6.8 | What is the proportion of all neonatal deaths in the health facility that were due to sepsis? |
| Confirm | Get this number from the hospital records/dashboard indicators |
| 6.9 | What is the proportion of all severe neonatal morbidity in the health facility that was due to neonatal sepsis? |
| Confirm | Get this number from the hospital records/dashboard indicators |

| | |
|---|---|
| **7.** | **Do all neonates and young infants receive care that includes standard precautions for preventing hospital-acquired infections?** |
| 7.1 | Does the health facility has a reliable water source on site and soap and towels (preferably disposable) or alcohol-based hand rub for hand hygiene? |
| Ask | |
| Confirm | |
| 7.2 | Does the health facility ensures safe handling, storage and final disposal of infectious waste? |
| Ask | |
| Confirm | |
| 7.3 | Does the health facility ensure safe handling, storage (puncture resistant) and final disposal of sharps waste? |
| Ask | |
| Confirm | |
| 7.4 | Does the health facility has appropriate sterilizing facilities and disinfectants for instruments? |
| Ask | |
| Confirm | |
| 7.5 | Does the health facility has a functioning incinerator or other appropriate method for treatment of infectious waste and used instruments? |
| Ask | |
| Confirm | |
| 7.6 | Does the health facility has written, up-to-date guidelines for standard infection control and precautions for transmission? |
| Ask | |
| Confirm | Look for printed copy of the protocol |
| 7.7 | Does the health care staff in the childbirth and neonatal areas receive training in standard infection control and precautions for transmission at least once every 12 months? |
| Ask | Last event: Where, when, by whom, who participated |
| Confirm | Look for any documentary proof |
| 7.8 | What percentage of health care staff in the health facility clean their hands correctly as per the WHO "5 |

| | |
|---|---|
| | moments for hand hygiene" audit tool? |
| Ask | Ask if this information is available as a dashboard indicator |
| Confirm | Note this by observing 20 opportunities for hand hygiene |
| 7.9 | What is the proportion of newborns with suspected severe bacterial infection who received appropriate antibiotic therapy? |
| Confirm | Check record of any 5 newborn or young infants who have suspected severe bacterial infection and note:<br>BW Gestation Antibiotic/route/dose/freq/duration Antibiotic/route/dose/freq/duration<br>1<br>2<br>3<br>4<br>5 |
| 7.10 | Is there safe management of health care waste, from the point of generation to the point of disposal? |
| Ask | Ask and draw below the process by which health care staff is processed: |
| 7.11 | What percentage of staff members in the health facility meet biosafety standards when administering parenteral drugs? |
| Confirm | By using the checklist while observing 5 staff members preparing and administering using the simulation exercise: ________________ |
| 7.12 | What proportion of all neonates born in the health facility develop hospital-acquired infections? |
| Ask | Ask for this information from the hospital record/dashboard indicator: _________ |
| Confirm | Total number of neonates born and admitted in the hospital at present: _______________<br>Total number who have suspected or proven HAI and are being treated for it: ________________ |

| **8.** | **Are newborn are subjected to unnecessary or harmful practices during childbirth and the early postnatal period?** |
|---|---|
| 8.1 | Does the health facility has written, up-to-date guidance on harmful practices and unnecessary interventions during childbirth and the early postnatal period? |
| Ask | |
| Confirm | Look for printed copy of the protocol |
| 8.2 | Does the health care staff in the facility receive in-service training and regular refresher sessions on harmful practices and unnecessary interventions at least once every 12 months? |
| Ask | Last event: Where, when, by whom, who participated |
| Confirm | Look for any documentary proof |
| 8.3 | Does the health facility does display infant formula or bottles and teats, including on posters or placards? |
| Confirm | During visit to the outdoor and indoor areas which serve newborn and paediatric patients |
| 8.4 | Is there a practice in the health facility of giving the newborn infants food or drink other than breast milk, unless medically indicated, and or pacifiers (also called “dummies” or “soothers”) to breastfeeding babies? |
| Ask | |
| Confirm | During visit to the postnatal ward |
| 8.5 | Does the health facility promote infant formula on the wards, and are formula samples distributed to mothers or staff? |
| Ask | |
| Confirm | |
| 8.6 | Does the health-care staff in the health facility receive monthly supportive supervision and mentoring on harmful practices and unnecessary interventions? |
| Ask | If yes, how and who does this activity? |
| 8.7 | Is there practice of giving bath to newborn infants in the hospital? |
| Ask | |
| Confirm | Ask mothers in the postnatal wards if their babies have received bath in the hospital? |
| 8.8 | Is routine oropharyngeal suction done in well newborn infants at the time of birth? |
| Ask | |

| | |
|---|---|
| 8.9 | What is the proportion of all babies born in the health facility who received early bathing and removal of the vernix within 6 h of birth? |
| Confirm | Total newborn infants assessed by asking this question: ________________<br>Number who received bath: _____________ |
| 8.10 | What is the proportion of well neonates who are admitted for treatment of jaundice and who are receiving intravenous fluids? |
| Confirm | Total well neonates admitted for phototherapy: _____________<br>Neonates who are on intravenous fluids: _______________ |

| | | | | | |
|---|---|---|---|---|---|
| **9.** | **Are sick neonates and young infants triaged and promptly assessed for emergency and priority signs, resuscitated and receive appropriate care according to WHO guidelines?** | | | | |
| 9.1 | Does the health facility has written, up to date clinical protocols for emergency triage and assessment and appropriate case management of sick neonates and young infants as per IMNCI/WHO guidelines? | | | | |
| Ask | | | | | |
| Confirm | Look for printed copy of the protocol | | | | |
| 9.2 | Does the health facility health professional staff receive in-service training/ regular refresher sessions at least once every 12 months in ETAT or NRP and IMNCI? | | | | |
| Ask | Last event: Where, when, by whom, who participated | | | | |
| Confirm | Look for any documentary proof | | | | |
| 9.3 | Is a triage system in place for every sick YI arriving at the facility to have a rapid visual inspection very soon after arrival which not hindered by administrative or payment procedures? | | | | |
| Ask | Where do the patients report (first contact point) and who assesses then infants? | | | | |
| 9.4 | Does the health facility receiving referred neonates or young infants with danger signs or emergency signs or injuries has a system in place for infants to receive a full initial assessment by suitably trained staff within 15 minutes of arrival? | | | | |
| Ask | Who, how and where assesses the referred patients in day and night times? | | | | |
| 9.5 | Does the health facility has an area or at least one clinical cubicle or trolley space in outpatient and wards that is designated for the management of infants with life threatening emergency conditions or injuries? | | | | |
| Ask | | | | | |
| Confirm | By visiting the area | | | | |
| 9.6 | Is the designated emergency care area or cubicle or trolley space in outpatient and wards well equipped with paediatric friendly essential medicines, supplies and equipment for emergency resuscitation and initial treatment? | | | | |
| Ask | | | | | |
| Confirm | | Birth area 1 | Birth area 2 | Postnatal ward | Neonatal area |
| Answer Y or N | Suction machine | | | | |
| | Suction catheters | | | | |
| | Oxygen source | | | | |
| | Preterm mask | | | | |
| | Term mask | | | | |
| | Self-inflating bag | | | | |
| | IV cannula | | | | |
| | IV fluids | | | | |
| | Phenobarbitone | | | | |
| | Diazepam | | | | |
| 9.7 | Does the designated emergency care area or cubicle or trolley space in outpatient and wards has resuscitation job aids (resuscitation algorithms, medication, fluids and other treatment charts, ETAT or NRP | | | | |

| | |
|---|---|
| | wall charts, chart booklets) that are visibly displayed |
| Ask | |
| Confirm | By visiting the area |
| 9.8 | Does the health facility conduct regular emergency care drills at least once every month for all staff working in paediatric emergency areas and on wards admitting severely ill young infants? |
| Ask | Last event: Where, when, by whom, who participated |
| Confirm | Look for any documentary proof |
| 9.9 | What is the proportion of all neonates and young infants who did not require urgent referral or admission and were properly assessed according to the IMCI guidelines? |
| Confirm | From records/dashboard |
| 9.10 | What is the proportion of all neonates and young infants who presented in emergency with general danger or emergency signs or injuries and were assessed within 15 minutes of arrival in the facility? |
| Confirm | From records<br><br>From currently admitted patients |
| 9.11 | What proportion of staff caring for neonates and young infants in the health facility have received training or refresher courses in ETAT/IMNCI or basic NRP in the last 12 months? |
| Ask | |
| Confirm | Check records |
| 9.12 | What proportion of clinical staff caring for neonates and young infants in the health facility are highly competent in ETAT or NRP? |
| Confirm | By skill assessment/simulation |
| 9.13 | What is the proportion of all neonates and sick young infants with general danger and emergency signs needing referral who received correct pre-referral treatment and are appropriately referred? |
| Confirm | Check records (referral register or referral slip copies if available) |
| 9.14 | What is the health facility age disaggregated child (neonates and 29-59 days) mortality rate (number of death/ total number presenting to health facility)? |
| Confirm | From record for last 3 months/last year |

| **10.** | **Are all neonates and sick young infants thoroughly assessed for possible serious bacterial infection or disease, and receive appropriate care?** | | |
|---|---|---|---|
| 10.1 | Does the health facility has a written, up-to-date clinical protocol for identification and management of children with PSBI or very severe diseases, local infections and jaundice in young infants consistent with WHO guidelines? | | |
| Ask | | | |
| Confirm | Look for printed copy of the protocol | | |
| 10.2 | Does the health facility staff caring for children receive IMNCI training/regular refresher sessions in the recognition and management of sick young infants at least once every 12 months.? | | |
| Ask | Last event: Where, when, by whom, who participated | | |
| Confirm | Look for any documentary proof | | |
| 10.3 | Are the healthcare professionals using the IMNCI guidelines to assess and record the risk of serious illness in sick young infants presenting at the health facility? | | |
| Ask | How sick young infants are assessed and how the assessment is recorded? | | |
| | | | |
| 10.4 | Does the referral receiving health facility for sick young infants with PSBI has basic laboratory and diagnostic tests available for appropriate investigation and management? | | |
| | Test | Yes/No | |
| | Blood sugar | | |
| | Blood counts | | |
| | Blood grouping and cross matching | | |
| | Blood culture | | |
| | Urine R/M | | |
| | CSF examination | | |
| | Chest-X-ray | | |
| 10.5 | Does the referral receiving facility has supplies and materials to provide optimal thermal care to stable and unstable preterm small babies, including kangaroo mother care (support binders, baby hats, socks), clean incubators and radiant warmers? | | |
| Confirm | Equipment/supplies | Total | Functional |
| | Incubators | | |
| | Radiant warmer | | |
| | KMC chairs | | |
| | Support binders/gowns/culturally appropriate tier for KMC | | |
| | Any other equipment used for thermal care: | | |
| | | | |
| | Total number of preterm or sick babies admitted at present in the health facility: | | |

| | |
|---|---|
| 10.6 | Does the health facility has supplies of injectable antibiotics (first-and second-line antibiotics for neonatal sepsis and meningitis) available in sufficient quantities at all times for the expected case load? |
| Ask | Ask and list antibiotics which are available and the number health facility gets every month<br>Antibiotic Number/month |
| Confirm | Check the stock for of antibiotics which are available and the number available<br>Antibiotic Number now available |
| 10.7 | What proportion of all sick young infants with a classification or diagnosis of PSBI or sepsis are prescribed the appropriate antibiotics (including correct choice of drug, correct dose for weight and age, frequency and route of administration) according to WHO/NNF/NHM guidelines? |
| Confirm | Check record of any 5 newborn or young infants who have suspected severe bacterial infection and note:<br>BW Gestation Antibiotic/route/dose/freq/duration Antibiotic/route/dose/freq/duration<br>1<br>2<br>3<br>4<br>5 |
| 10.8 | Are sick young infants with signs such as not feeding well, convulsions, severe chest indrawing, fever > 37.5, poorly responsive and RR >60 breaths per min. are appropriately classified as PSBI or sepsis and investigated? |
| Confirm | Check by going through the records and history of eligible babies currently admitted |
| 10.9 | What is the proportion of all sick young infants admitted in the facility with suspected PSBI or sepsis who have been appropriately investigated (e.g. FBC, Culture, lumbar puncture). |
| Confirm | Check by going through the records and history of eligible babies currently admitted |
| 10.10 | Does the referral receiving health facility has a separate special area or room where sick young infants are admitted and managed? |
| Ask | |
| Confirm | By visiting the area |
| 10.11 | Does the health facility has and uses a standard sick young infant monitoring chart documenting and monitoring patient condition and clinical progress (patient details, vital signs, clinical signs, treatments given, feeding and outcome)? |
| Ask | |
| Confirm | By checking the records of current or recent admissions |

| | |
|---|---|
| 10.12 | Does the referral receiving health facility has facilities for kangaroo mother care and practices rooming in of parents with their sick infants? |
| Ask | |
| Confirm | By visiting the area |
| 10.13 | What is the proportion of small young infants less than 2000 gm who received KMC (Kangaroo mother care) as part of clinical management during their care in the health facility? |
| Confirm | From records/dashboard indicators: ___________________________________<br><br>Asking mothers of all eligible neonates: ____________________________ |
| 10.14 | What is the proportion of all sick young infants admitted in the facility with suspected PSBI or sepsis whose need of oxygen was appropriately assessed, documented and delivered? |
| Confirm | By checking the records of admitted infants |
| 10.15 | What is the proportion of all sick young infants admitted in the facility with convulsions who were appropriately investigated and treated with anticonvulsants |
| Confirm | By checking the records of admitted infants |
| 10.16 | Does the referral receiving health facility for young infants with severe jaundice has procedures in place to check the bilirubin, and for exchange transfusion when required? |
| Ask | Check for the following:<br>Yes/No Limited hours Round the clock<br>Serum bilirubin measurement<br>Phototherapy machine<br>Blood bank<br>Blood group and cross match<br>Skilled physician to do ET |
| 10.17 | What is the proportion of all sick young infants with a classification or diagnosis severe jaundice whose bilirubin level was checked and appropriately managed according to NNF/AAP/NHM/WHO guidelines? |
| Confirm | By checking the records of admitted infants the following<br><br>Admission bilirubin<br>Bilirubin every 4-24 h<br>Correct level to start phototherapy<br>Correct level to stop phototherapy<br>Correct level for ET |
| 10.18 | What is the proportion of all sick young infants treated for PSBI/sepsis who died in the health facility (case fatality rate)? |
| Confirm | From last year/last 3 months record<br><br>Total infants with PSBI/sepsis: _________________<br>Infants who died: ______________________ |

| | |
|---|---|
| 10.19 | What is health facility neonatal (0-28 days) mortality rate? |
| Confirm | From last year/last 3 months record<br><br>Total infants born: ________________<br>Infants who died: ______________________ |
| 10.20 | What is birth weight segregated mortality rate? |
| Confirm | From last year/last 3 months record<br><br>Total born Number dies BW specific mortality rate per 1000<br>2000-2499 g<br>1500-1999 g<br><1500 g |

| **11.** | **Are all young infants with diarrhoea correctly assessed, classified and receive appropriate rehydration plan and care including continued feeding according to guidelines?** |
|---|---|
| 11.1 | Does the health facility has a written, up-to-date clinical protocol for identification and management of diarrhoea including for severe dehydration consistent with WHO guidelines? |
| Ask | |
| Confirm | Look for printed copy of the protocol |
| 11.2 | Does the health facility clinical staff caring for children receive training and regular refresher sessions in the assessment and management of children with diarrhoea with dehydration or dysentery at least once every 12 months? |
| Ask | Last event: Where, when, by whom, who participated |
| Confirm | Look for any documentary proof |
| 11.3 | Does the healthcare professionals use the IMNCI guidelines to assess, document and appropriately manage young infants with diarrhea with dehydration or dysentery at the health facility? |
| Ask | |
| 11.4 | What is the proportion of all children with diarrhea who were correctly assessed, classified and managed using an appropriate rehydration plan? |
| Confirm | By looking at records of admitted/recently discharged neonates |
| 11.5 | Does the health facility pediatric outpatient areas and wards have rehydration algorithms and plan A, B & C charts available and visibly displayed on the walls for the sue by both health workers and parents? |
| Ask | |
| Confirm | By visiting the area |
| 11.6 | Does the health facility has supplies (IV fluids, ORS, Zinc, Antibiotics) available in sufficient quantities always for the expected case load? |
| Confirm | IV fluids<br>ORS<br>Zinc<br>Antibiotics (with name) |
| 11.7 | What is the proportion of all young infants with diarrhea who were correctly prescribed antibiotics? |
| Confirm | Check records of admitted/recent patients |

| | |
|---|---|
| 11.8 | What is the proportion of all young infants with diarrhea who were correctly prescribed IV fluids? |
| Confirm | Check records of admitted/recent patients |
| 11.9 | What is the proportion of all young infants with diarrhea whose parents know how to prepare and administer ORS, give extra fluids and continue feeding their children? |
| Confirm | Interview parents of currently available patients |
| 11.10 | What is the proportion of all young infants managed as diarrhea with severe dehydration in the health facility who died of diarrhea? |
| Confirm | Check from dashboard indicators/hospital records |
| 11.11 | What is the proportion all young infants admitted in the health facility who died of diarrhea with dehydration? |
| Confirm | Check from dashboard indicators/hospital records |

| 12. | **Are all neonates and young infants assessed for growth and breast-feeding, and their parents receive appropriate nutritional support and counselling according to WHO guidelines?** |
|---|---|
| 12.1 | Does the health facility maintain the baby-friendly hospital status that supports breastfeeding and other critical interventions consistent to WHO guidelines? |
| Ask | |
| Confirm | Look for certification |
| 12.2 | Does the health facility clinical staff caring for children receive training and regular refresher sessions in the assessment and management of children with diarrhoea with dehydration or dysentery at least once every 12 months? |
| Ask | Last event: Where, when, by whom, who participated |
| Confirm | Look for any documentary proof |
| 12.3 | Does the health facility has a written up to date breastfeeding policy that is routinely communicated to all health care and support staff? |
| Ask | |
| Confirm | Ask for a printed copy |
| 12.4 | Does the facility fully complies with the Infant Milk Substitute (IMS) Act of India? |
| Ask | |

| | |
|---|---|
| **13.** | **Are all young infants assessed and checked for immunization status and receive appropriate vaccinations according to expanded programme for immunization guidelines?** |
| 13.1 | Does the health facility has written, up-to-date protocols for immunization of children consistent with national guidelines? |
| Ask | |
| Confirm | Look for protocol copy |
| 13.2 | Does the health facility has supplies and materials to provide age appropriate initial doses of immunization to eligible neonates and young infants at all times |
| Ask | |
| Confirm | Look for BCG, OPV, Pentavalent vaccine, Hepatitis B |
| 13.3 | Does the facility has a system for all vaccines and their diluents to be stored and distributed within a cold-chain system that maintains, at all times, the WHO-recommended temperatures ranges? |
| Ask | |
| Confirm | Look for ice line refrigerator and vaccine carrier box |
| 13.4 | Does the facility has systems and procedures for managing the stocks of vaccines are effective, in terms of vaccine handling, physical inventory, stock-control, adequate stock-level policy, good warehousing practice, and disposal procedures for damaged and expired vaccines? |
| Ask | |
| 13.5 | Does the health care staffs receive in-service training and regular refresher sessions on immunization of children at least once every 12 months? |
| Ask | Last event: Where, when, by whom, who participated |
| Confirm | Look for any documentary proof |
| 13.6 | What is the proportion of all neonates and young infants checked for immunization and correctly received age appropriate vaccination? |
| Confirm | Look at records of all available neonates and young infants<br>Neonates<br><br>Young infants |
| 13.7 | What is the proportion of all immunization eligible children whose mothers received counseling when to bring the child for additional vaccinations? |
| Ask | Mothers of neonates coming out of vaccination room |

| 14. | **Do all sick neonates and young infants receive adequate monitoring, periodic clinical reassessment, nutritional and supportive care according to WHO guidelines with attention directed to the most seriously ill?** |
|---|---|
| 14.1 | Does the health facility has written, up-to-date protocols for monitoring and supportive care for neonates and young infants according to the severity of their condition? |
| Ask | |
| Confirm | Look for protocol copy |
| 14.2 | Does the health facility has patient monitoring charts that include provision for recording details (what, how much and when) of supportive care provided? |
| Ask | |
| Confirm | Look for a copy of the charts |
| 14.3 | What is the proportion all unstable neonates and young infants admitted the wards that had a oro/nasogastric tube passed for feeding? |
| Confirm | Look at all the eligible infants |
| 14.4 | What is the proportion of all neonates and young infants receiving oxygen who had documented prescription of method and rate of oxygen delivery? |
| Confirm | Look at all the eligible infants |
| 14.5 | What is the proportion of all neonates and young infants admitted to the health facility who were appropriately monitored and have the observations recorded by a nurse at least twice a day? |
| Confirm | Look at the nursing charts of eligible infants |
| 14.6 | What is the proportion of all neonates and young infants admitted to a health facility who are reassessed daily during working days by a doctor or clinical officer trained child health care provider? |
| Confirm | Look at the doctor notes of eligible infants |
| 14.7 | What is the proportion of all breastfed infants unable to breast who received expressed breast milk by oro/nasogastric tube while in hospital? |
| Confirm | Look at all the eligible infants |
| 14.8 | What is the proportion of all young infants with very high temperature (>$38.5^0$) who were prescribed appropriate antipyretic treatment (choice, dose and frequency) to reduce temperature? |
| Confirm | Look at all the eligible infants |

| **15.** | **Do newborns and young infants have a complete, accurate, standardized medical record during childbirth, early postnatal period and admission during sickness which is accessible during the process of care, on discharge and follow-up?** |
|---|---|
| 15.1 | Does the health facility has standardized child care registers, clinical medical records and observation charts in place at all time for recording and monitoring of all care provided to neonates and young infants? |
| Ask | |
| Confirm | Look for copy |
| 15.2 | Does the health facility has and uses neonate/young infant specific standardized clinical charts for history taking and clinical examination, and critical pathway patient monitoring charts? |
| Ask | |
| Confirm | Look at the records |
| 15.3 | Does the health facility has birth, admission, discharge and death registration system in place that is linked to the national vital registration system at all times? |
| Ask | |
| Confirm | Look at the records |
| 15.4 | Does the health facility has a system for classifying diseases and outcomes including death, which is aligned with the ICD? |
| Ask | |
| Confirm | Look at the records |
| 15.5 | What is the proportion of neonatal/young infants' medical records in the facility where patient identification and demographic information includes the name of the child and the person(s) with legal parental responsible? |
| Confirm | Look at the admitted patient records |
| 15.6 | What is the proportion of all neonates and young infants currently in the health facility who have a patient identifier and individual clinical medical record? |
| Confirm | Look at the admitted patient records |
| 15.7 | What is the proportion of neonates' medical records that have birth weight recorded? |
| Confirm | Look at the admitted patient records |

| | |
|---|---|
| 15.8 | What is the proportion of neonates' medical records that have gestation at birth recorded? |
| Confirm | Look at the admitted patient records |
| 15.9 | What is the proportion of medical records of discharged patients within the past 24 h with correct and complete discharge summary information with diagnosis and outcomes consistent with ICD classification? |
| Confirm | Look at the records |
| 15.10 | What is the proportion of medical records where every entry in the medical record was dated, timed (24-hour clock), legible and signed by the person making the entry? |
| Confirm | Look at the records |

| 16. | **Do the health facility has a mechanism for data collection, analysis and feedback as part of its activities for monitoring and improving performance of care for neonates and young infants?** |
|---|---|
| 16.1 | Does the health facility has standard operating procedures and protocols in place at all times for checking, validating and reporting data? |
| Ask | |
| Confirm | Look for copy |
| 16.2 | Has the health facility conducted reviews of perinatal, neonatal and young infant deaths and near-misses at least once a month within the past six months and has a mechanism for implementing the recommendations of reviews? |
| Ask | |
| Confirm | Look for copy |
| 16.3 | Has the health facility data system for collecting and analysing relevant indicators and can produce visual outputs and timely reporting on paper or digitally at all times? |
| Ask | |
| Confirm | Look for a copy |
| 16.4 | Do managers and health care workers in the health facility meet at least once a month to review process and outcome data? |
| Ask | |
| Confirm | Look for evidence of meetings |
| 16.5 | Do managers and health care workers in the health facility use the recommendations in reviews of data for decision-making and for mentoring improved performance? |
| Ask | |
| Confirm | Ask for an example in which this activity was done |
| 16.6 | Does the facility has up to date monthly patient morbidity and mortality statistics for neonates and young infants? |
| Ask | |
| Confirm | Ask for copy of last 3 months, statistics |
| 16.7 | What proportion of all neonatal deaths occurring in the health facility in the preceding month were reviewed with standard audit tools? |

| | |
|---|---|
| Confirm | Ask for audit copies |
| 16.8 | What proportion of all recommendations of neonatal death reviews at the health facility in the last six months that were fully implemented? |
| Confirm | Ask for documents/examples of implementation |
| 16.9 | What proportion of health facility monthly reports were received timely by next high level of administration in the preceding 12 months? |
| Ask | |

| 17. | **Does the health facility has a mechanism in place to collect, analyse and provide feedback on services provided and perception of care by the children and/their families?** |
|---|---|
| 17.1 | Does the health facility has a protocol for collecting data on perception of services from families of neonates and young infants? |
| Ask | |
| Confirm | Look for copy |
| 17.2 | Does the health facility has visible information materials or posters informing families about availability of complaints/suggestion boxes and feedback mechanisms? |
| Confirm | |
| 17.3 | Does the health facility has an established and functional patient complaints/suggestion box and feedback system in place? |
| Ask | |
| Confirm | |
| 17.4 | What proportion of carers are aware of the availability of a patient complaints/suggestion box and feedback system in the health facility? |
| Confirm | Ask from 10 carers available in the facility |
| 17.5 | What proportion of families have participated in the patient satisfaction surveys or provided feedback on services received in the last 3 months? |
| Confirm | Ask for evidence |
| 17.6 | What proportion of carers are fully aware of their care plan, diagnosis and reasons for admission? |
| Confirm | Ask from 10 carers available in the facility |
| 17.7 | What proportion of carers of neonates or young infants admitted to the wards are satisfied with the spent waiting for care before admission on the wards? |
| Confirm | Ask from 10 carers available in the facility |
| 17.8 | What proportion of carers will recommend the health facility or service provided to family and friends? |
| Confirm | Ask from 10 carers available in the facility |

| 18. | **Are neonates and young infants appropriately assessed at the time of birth or admission and during stay in the hospital to determine whether referral is required, and is the decision to refer made without delay?** |
|---|---|
| 18.1 | Does the health facility has written, up-to-date clinical protocols and guidelines for the identification, management (including pre-referral care) and referral of neonates and young infants.? |
| Ask | |
| Confirm | Look for copy |
| 18.2 | Are wall charts or job aids for identifying neonates and young infants by severity of condition visibly located in the emergency area and all areas where neonates and young infants managed? |
| Ask | |
| Confirm | Look |
| 18.3 | Are neonates and young infants weighed except for those who require emergency stabilization, and the weight and vital signs (temperature, HR and respiratory rate) clearly recorded? |
| Ask | |
| Confirm | Look at records of infants brought in emergency |
| 18.4 | Is the health facility equipped with appropriate medicines and supplies for stabilization and pre-referral treatment for of critically ill neonates and young infants requiring referral? |
| Confirm | Look at supplies available in the emergency area |
| 18.5 | Do health professionals and staff caring for children in the health facility receive in-service training to safely assess, manage, and transport common neonatal emergencies to a pre-defined level within the network; this includes scene awareness and calming? |
| Ask | Last event: Where, when, by whom, who participated |
| Confirm | Look for any documentary proof |
| 18.6 | Does the health facility has at least one health professional trained and competent in first aid and basic NRP skills or ETAT? |
| Ask | |
| Confirm | Look for any documentary proof |

| | |
|---|---|
| 18.7 | What is the proportion of all neonates and young infants who could not be managed at the health facility who were transferred to a higher-level facility for further management without delay (within 1 h), accompanied by a health care professional (if appropriate) and a completed standardized referral note? |
| Confirm | Find the number referred in last 3 months and the number who fulfilled the above criteria |
| 18.8 | What is the proportion of neonates/young infants referred to another health facility whose families reported receiving immediate attention on arrival at the health facility? |
| Confirm | Ask by contacting the families of referred patients |

| 19. | **Do neonates and young infants who requires referral receive seamless coordinated care and the referral follows a plan that can be implemented in a timely manner?** |
|---|---|
| 19.1 | Is there an up-to-date list of the network facilities in the same geographical area that provide referral care for neonates and young infants? |
| Ask | |
| Confirm | Look for copy |
| 19.2 | Does the health facility has ready access to a functioning ambulance or other vehicle for emergency transport of infants and their families to referral facilities? |
| Ask | |
| 19.3 | Does the health facility has local financial arrangements to ensure that infants who cannot be managed at the health facility are referred without delay, 24 h/d/ 7 days a week? |
| Ask | |
| 19.4 | Does the health facility has at least one health professional with advanced training and competent in full NRP available to accompany the severely ill infants and their family to the receiving facility? |
| Ask | |
| Confirm | Ask for documentary proof |
| 19.5 | Does the health facility ambulance (transport vehicle) carries basic equipment suitable for resuscitation of neonates and young infants? |
| Ask | |
| Confirm | Observe and list or get a list of the equipment available in the ambulance |
| 19.6 | What proportion of neonates and young infants died before or during transfer to higher-level facility for further management? |
| Confirm | Check from facility record or referral register of last 3 months |
| 19.7 | What proportion of neonates and young infants were referred without appropriate emergency transport? |
| Confirm | Check from facility record or referral register of last 3 months |

| | |
|---|---|
| 19.8 | What proportion of all newborns and young infants referred from the health facility completed their referral? |
| Confirm | Check from facility record or referral register of last 3 months |

| 20. | **Is there an appropriate information exchange, and feedback to relevant health care staff for every neonate or young infant referred and/ counter referred within or between health facilities?** |
|---|---|
| 20.1 | Does the health facility has a standardized referral form to document relevant demographic and summary clinical information on history, diagnosis, vital signs, and treatment given and reason for referral.? |
| Ask | |
| Confirm | Look for copy |
| 20.2 | Has the health facility reliable communication methods (mobile phone, landline or radio) functioning at all times, for referrals and tele consultations? |
| Ask | |
| 20.3 | Does the health professional from the referral receiving health facilities provide support to referring facilities on quality improvement and education on neonatal and young infant emergencies? |
| Ask | |
| Confirm | Who, when and how this activity was conducted last |
| 20.4 | For what proportion of all referred children seen at the referring facility there was complete counter-referral feedback information? |
| Confirm | Check referral register record |
| 20.5 | Does the health facility has formal agreements, communication arrangements and a feedback system with referral center(s)? |
| Ask | |
| Confirm | For documents |

| | |
|---|---|
| **21.** | **Do families have effective interactions with staff, are given information about the illness and care offered to enable them to understand and cope with the illness or injury, and the treatment needed?** |
| 21.1 | Does the health facility has a policy which provides that parents or carers are entitled to receive full and effective information regarding children's care and other relevant aspects during their stay in a health facility? |
| Ask | |
| Confirm | Look for copy |
| 21.2 | Does the health facility routinely distributes information materials to families to help them understand the opportunities that exist for engagement, how to participate in their care, and the roles of the different health care team members? |
| Ask | |
| Confirm | Look for copy |
| 21.3 | Are there visible posters or flyers in areas where neonates and young infants are cared for that remind the the families of the importance of being partners and what they can do? |
| Ask | |
| Confirm | |
| 21.4 | Does the health facility has a system in place of providing information to families about their medical conditions and treatment care plan in a way that is understandable to them? |
| Ask | |
| 21.5 | Does the health care staff receive training and 12 monthly refresher training on how to explain fully to children about their condition, and how to give 'bad news'? |
| Ask | |
| Confirm | Who, how and where the last training took place |
| 21.6 | Does the health care staff in the maternity unit receive supportive supervision in interpersonal communication, counselling and cultural competence every three months? |
| Ask | |

| | |
|---|---|
| 21.7 | Does the health facility has written information for common conditions, and is written in simple language and languages relevant for the patient population, using diagrams where appropriate, to aid understanding? |
| Ask | |
| Confirm | Look for copy |
| 21.8 | What proportion of health care staff in the health facility are wearing identification badges? |
| Confirm | By observation |
| 21.9 | What proportion of families of admitted neonates/young infants were aware of their disease or condition and the reason for admission? |
| Confirm | |
| 21.10 | What proportion of of neonates/young infants cared for in the health facility have been discharged who or whose families can describe correctly how to give the discharge treatment at home? |
| Confirm | Ask parents whose neonates are being discharged |
| 21.11 | What proportion of parents or carers felt they were adequately informed by the healthcare staff about the examinations, actions and decisions taken for their care? |
| Confirm | |
| 21.12 | What proportion of all women discharged from after childbirth received written and verbal information and counselling on the following elements before discharge: nutrition and hygiene, birth spacing and family planning, exclusive breastfeeding and maintaining lactation, keeping their baby warm and clean, communication and play with the baby, danger signs for the mother and newborn and where to go in case of complications? |
| Confirm | |
| 21.13 | What proportion of parents reported that they were satisfied with the health education and information they received from the healthcare staff? |
| Confirm | |

| 22. | **Do families experience coordinated care, with clear, accurate information exchange between relevant health and social care professionals, and other staff?** |
|---|---|
| 22.1 | Des health facility has written, up-to-date standardised processes, policies and procedures for the process of handover of patients within and between the facilities? |
| Ask | |
| Confirm | Look for copy |
| 22.2 | Does the health facility has a standard form with minimum data set (information content) and conduct of handover delivered in a structured format to facilitate written hand-over? |
| Ask | |
| Confirm | Look for copy |
| 22.3 | Does the health facility has written, up-to-date protocols for verbal and written hand-over of patients at shift changes, during intra-facility transfer, on referral to other facilities and at discharge? |
| Ask | |
| Confirm | Look for copy |
| 22.4 | Does the health-care staff caring for neonates and young infants oriented and receive in-service training and regular refresher sessions at least once every 12 months in the clinical hand-over policy and communication of important information for hand-over, referral or discharge? |
| Ask | |
| Confirm | When, how and who conducted the last session |
| 22.5 | Does the health facility has a functioning, reliable in facility communication system for information exchange among relevant service providers, and can reach all critical staff 24 h 7 days a week? |
| Ask | |
| 22.6 | What proportion of patient clinical records contain evidence that all imaging reports, investigation reports and clinical correspondence received by or performed in the facility were reviewed by health care staff, signed or initialled, and acted upon in a timely manner? |
| Confirm | Check 10 records |

| | |
|---|---|
| 22.7 | What proportion of neonates or young infants admitted have an up to date and appropriately completed critical pathway monitoring forms/sheets with the regularly monitored vital signs? |
| Confirm | Check 10 records |
| 22.8 | What proportion of staff caring for neonates and young infants in the health facility can describe how follow up and recall of patients with clinically significant tests and results is done as per health facility protocol? |
| Confirm | Ask 10 HCP |
| 22.9 | What proportion of families of neonates and young infants admitted in the health facility reported that know their primary health care provider by name? |
| Confirm | Ask 10 set of parents |
| 22.10 | What proportion of health staff in the health facility are fully aware of the clinical handover policies, procedures and/or protocols and regularly use them for handover of patients during the course of care? |
| Confirm | Ask 10 HCP |
| 22.11 | What proportion of all carers of neonates and young infants admitted in the health facility expressed satisfaction with the health services? |
| Confirm | Ask 10 set of parents |
| 22.12 | What proportion of health care staff, by cadre, and social professionals were satisfied with the communication during clinical hand-over among members of the health care team in the health facility? |
| Confirm | Nurses<br><br>Doctors |

| | |
|---|---|
| **23.** | **Are families of neonates and young infants enabled to actively participate during the process of care, in decision making, in exercising the right to informed consent and choice?** |
| 23.1 | Does the health facility has up to date protocols, guidelines and job aides that provide information to families about the purpose, importance, benefits, risks and possible costs of proposed investigations, referrals or treatments? |
| Ask | |
| Confirm | Look for copy |
| 23.2 | Does the health facility has protocols in place listing the interventions that require informed consent from parents? |
| Ask | |
| Confirm | Look for copy |
| 23.3 | Does the health facility has a range of visual resources (models, charts, posters, videos, electronic materials) available in the consulting room for clinical staff and other health professionals to use when explaining to families? |
| Ask | |
| Confirm | Look for resources |
| 23.4 | Does the health facility has appropriate forms for parents or carers to sign their consent to procedures, investigations and treatment, and where consent is given in oral form, it is registered in the patient's chart? |
| Ask | |
| Confirm | Look at 10 patient records or forms |
| 23.5 | Are the health facility staff fully aware of parents' involvement in the care and the right to informed consent, and receive continuous medical education on communication skills at least once every 12 months? |
| Ask | |
| Confirm | |
| 23.6 | What proportion of families received written or verbal information about their right to freely express their views during their care and how this participation will take place? |
| Confirm | Ask 10 parents |

| | |
|---|---|
| 23.7 | What is the proportion of neonates and young infants in the health facility whose medical records have documented evidence that possible risks or side effects associated with treatment options have been discussed and explained to the families? |
| Confirm | |
| 23.8 | What is the proportion of families in the health facility whose medical records have documented evidence that possible risks or side effects associated with treatment options have been discussed and explained? |
| Confirm | |
| 23.9 | What is the proportion of staff caring for children in the health facility who correctly demonstrate how to use leaflets, brochures or written or electronic information to support explanation of the diagnosis and management of conditions when appropriate? |
| Confirm | |
| 23.10 | What is the proportion of families who found that the information provided to facilitate shared decision making was understandable and was clearly communicated? |
| Confirm | |
| 23.11 | What is the proportion of parents or carers in the health facility who were offered the option and were present with their child during medical procedures, and they were offered support before, during, and after the procedure? |
| Confirm | |

| **24.** | **Do families receive appropriate counselling and health education on the current illness, and on promotion of health and wellbeing?** |
|---|---|
| 24.1 | Do the health facility have health education and promotion information materials (leaflets, posters) that can be distributed to parents (e.g. on breastfeeding and nutrition support, immunization, diarrhoea and pneumonia). |
| Ask | |
| Confirm | Look for copy |
| 24.2 | Does the health facility has information materials for distribution to parents about promoting appropriate hygiene, water and sanitation practices? |
| Ask | |
| Confirm | Look for copy |
| 24.3 | Does the health facility provide an individual child health record booklet for every child upon birth or the first visit at the health facility, which is kept by the parent or carer, and used by the health providers to document relevant health information? |
| Ask | |
| Confirm | Look for a copy |
| 24.4 | Does the health facility hold regular wellbeing clinic services (e.g. well child and immunization clinics, counselling services, growth and development monitoring clinics) as an opportunity for health promotion and preventive care? |
| Ask | Which clinics and on which days? |
| 24.5 | Does the health facility has an alert system in place, whereby it detects if a young infant has missed a regular health appointment or failed a vaccine, under the national immunisation programme? |
| Ask | |
| 24.6 | What is the proportion of neonates or young infants in the health facility with child health record booklet? |
| Confirm | |
| 24.7 | What is the proportion of parents who have attended at least one health education or promotion session at the health facility? |
| Confirm | |

| 25. | **Do neonates and young infants have the right to access health care and services without discrimination of any kind?** |
|---|---|
| 25.1 | Does the health facility has an up to date policy in place that exempts neonates and young infants fee for service and guarantees an affordable healthcare in line with national policy? |
| Ask | |
| 25.2 | Does the health facility has an update non-discrimination policy, and mechanism in place for all children accessing care including the poor, vulnerable and disabled children? |
| Ask | |
| Confirm | Look for a copy |
| 25.3 | What proportion of parents or carers did not have to give any out-of-pockets payments for treatments, medicines, interventions or other health care-related costs? |
| Confirm | Ask from 10 parents |
| 25.4 | What proportions of families with a minority background, migrant status or belonging to another vulnerable group demonstrated their satisfaction in relation to the services provided? |
| Confirm | Ask from 10 parents of reserved class/minority status |

| **26.** | **Are families cared for with respect and dignity, and are their right to privacy and confidentiality is respected?** |
|---|---|
| 26.1 | Does the health facility has an update ethical, legal, and privacy guidelines and policies to the collection, maintenance, use, and dissemination of identifiable health information and monitors access to that information? |
| Ask | |
| Confirm | Look for the copy |
| 26.2 | What proportion of families in the health facility perceived and felt that were treated with dignity, kindness, compassion, courtesy, respect, understanding and honesty? |
| Confirm | Ask parents |
| 26.3 | Does the physical environment of the health facility allows privacy and the provision of respectful, confidential care, including the availability of curtains, screens, partitions and sufficient bed capacity? |
| Confirm | |

| **27.** | **Are neonates and young infants protected from human rights violations, physical or mental violence, injury, abuse, neglect or any other form of maltreatment?** |
|---|---|
| 27.1 | Does the health facility has a system whereby the mothers of small, sick newborns can be close to and nurse their babies? |
| Ask | |
| Confirm | |
| 27.2 | Is the fee structures for maternity and newborn care are equitable, affordable and clearly displayed? |
| Ask | |
| Confirm | |
| 27.3 | Does the health facility has a written, up-to-date policy and protocols that outline families ' right to make a complaint about the care received and has an easily accessible mechanism (e.g. a box) for handing in complaints? |
| Ask | |
| Confirm | Ask for a policy of the copy<br><br>Complaint box |
| 27.4 | What proportion of families who attended the health facility were refused care because of their inability to pay for the care of neonate/young infant? |
| Confirm | Look at reasons of referral/LAMA<br>Ask for health care providers |

| 28. | **Do neonates and young infants have access to safe and adequate nutrition during their care in the facility, that is appropriate for both their age and health condition?** |
|---|---|
| 28.1 | Does the health facility has an up to date written breastfeeding policy that that protects breastfeeding by adhering to the International Code of Marketing of Breast-milk Substitutes, and is routinely communicated to all health care staff? |
| Ask | |
| Confirm | Ask for a copy |
| 28.2 | Is the staff trained to show mothers how to breastfeed and how to maintain lactation, even if they should be separated from their infants? |
| Ask | |
| Confirm | Ask 1-2 staff members to counsel mothers in front of you |
| 28.3 | What proportion of mothers who are not directly breastfeeding report that they were shown how to express their milk by hand or given written information and told where they could get help? |
| Confirm | Ask mothers of neonates and young infants who are admitted in the facility and are not on direct breastfeeding |

| | |
|---|---|
| **29.** | **Is every neonate or young infant allowed to be with their carers, and the role of carers is recognised and supported at all times during the process of care, including rooming in during the course of admission?** |
| 29.1 | Does the health facility has an up to date family-centred policy on parents' and carers' right to stay with their infant at all times, including during procedures, i.e. injections, blood extractions, invasive diagnostic procedures or anaesthesia induction? |
| Ask | |
| Confirm | Ask for a copy |
| 29.2 | Does the health facility has rooming policy for parents to stay with their children, and provides accommodation as close to the child's bedside as desired? |
| Ask | |
| Confirm | |
| 29.3 | Does the health facility provide parent caring for their child in hospital meals? |
| Ask | |
| 29.4 | Does the health facility has policies supporting parents, i.e. food vouchers, a parents' room, a breastfeeding room, parents' support groups, among other actions |
| Ask | |
| 29.5 | Does the health facility make available a guide for parents or similar, summarizing necessary information about their child's stay in the health facility, i.e. facilities/services available for parents, how the care will take place, etc? |
| Ask | |
| Confirm | Ask for a copy of the guide |
| 29.6 | What proportion of parents in the health facility received a guide or other information about their rights and availability of rooming facilities during the course of admission of their child in the health facility? |
| Confirm | Ask parents of neonates and young infants admitted in the health facility |
| 29.7 | What proportion of parents in the health facility stayed with their children at all times during medical procedures? |
| Confirm | Ask parents of neonates and young infants admitted in the health facility |

| | |
|---|---|
| **30.** | **Are neonates and young infants routinely assessed for any presence of pain and distressing symptoms, and receives appropriate management?** |
| 30.1 | Does the health facility has in place up to date policies, protocols and procedures in for the assessment, prevention and management of pain/ |
| Ask | |
| Confirm | Ask for a copy |
| 30.2 | Do the health facility children's clinical medical record charts have a section that guides clinical staff in the assessment of pain? |
| Ask | |
| Confirm | |
| 30.3 | Does the health facility give adequate analgesia as a routine part of any treatment or procedure? |
| Ask | |
| 30.4 | Does the health staff receive training in the prevention, assessment and control of pain and regular refresher training in pain management at least once every 12 months? |
| Ask | |
| Confirm | Who, when, where and how |
| 30.5 | What proportion of health professional staff are able to carry out both pharmacological and non-pharmacological interventions to manage pain? |
| Confirm | |
| 30.6 | What proportion of infants' clinical records reviewed had written assessment or pain score card as part of the child's clinical record? |
| Confirm | |
| 30.7 | What proportion of infants received adequate analgesia before undergoing an invasive procedure? |
| Confirm | |
| 30.8 | What proportion of parents in the facility were satisfied with the pain control and management of their children during procedures and treatment? |
| Confirm | |

| **31.** | **Do neonates and young infants have access at all times to at least one skilled birth attendant and support staff for routine care and management of complications?** |
|---|---|
| 31.1 | Does the health facility have skilled child health care providers available at all times, in sufficient numbers to meet the anticipated work load? |
| Ask | |
| Confirm | Duty roster |
| 31.2 | Does the health facility have skilled birth attendants available at all times, in sufficient numbers to meet the anticipated work load? |
| Ask | |
| Confirm | |
| 31.3 | Does the health facility have a written, up-to-date staffing policy, listing the numbers, types and competency of staff, that is reviewed continuously according to the work load? |
| Ask | |
| Confirm | Copy of the protocol |
| 31.4 | Is a roster displayed in all areas, giving the names of staff on duty, the times of their shifts and their specific roles and responsibilities? |
| Confirm | |
| 31.5 | Does the health facility have a written, up-to-date policy on triage and waiting times for emergency and non-emergency consultations and treatment? |
| Ask | |
| Confirm | |
| 31.6 | Does the health facility have clear communication channels to reach staff on duty at all times? |
| Ask | |

| | |
|---|---|
| 31.7 | What proportion of available posts in the health facility were filled by staff with the necessary competency for the job description to allow the facility to provide 24-h service? |
| Ask | *Cadre* *Total sanctioned posts* *Posts filled* |
| 31.8 | What proportion of staff have been oriented to their functions, roles and responsibilities in the facility or unit to which they are assigned? |
| Confirm | |
| 31.9 | What proportion of families at the facility reported having been informed about danger signs and treatment provided and when to bring back the facility? |
| Confirm | |
| 31.10 | What proportion of women and their newborns in the health facility were attended by a skilled birth attendant (as per the definition) during and after childbirth? |
| Confirm | |
| 31.11 | What proportion of young infants in the health facility were attended by specifically trained health in child care? |
| Confirm | |

| 32. | **Do skilled birth attendants, health care professionals and support staff have appropriate competence and skills to meet requirements during labour, childbirth and the postnatal period?** |
|---|---|
| 32.1 | Does the health facility have a programme for continuing professional and skills development for all health care professionals and support staff? |
| Ask | |
| Confirm | |
| 32.2 | Does the health facility have standard procedures and plans for recruitment, deployment, motivation (recognition and reward scheme) and retention of all staff? |
| Ask | |
| 32.3 | Does the health facility periodically appraise all staff and has a mechanism for recognizing good performance? |
| Ask | |
| 32.4 | Does the health facility have sufficient numbers of educated, competent, licensed, motivated, regulated child health professionals with an appropriate skills mix, working in multidisciplinary teams? |
| Confirm | |
| 32.5 | What proportion of health care providers at the health facility received a written job description on deployment to the facility? |
| Confirm | |
| 32.6 | What proportion of health professionals caring for neonates or young infants received in-service training, a refresher session or mentoring within the past 12 months? |
| Confirm | |
| 32.7 | What proportion of staff at the health facility were assessed at least once in the preceding 12 months? |
| Confirm | |
| 32.8 | How many team meetings are held per month to review competence and quality improvement activities? |
| Ask | |
| 32.9 | What proportion of all staff at the facility are engaged in at least 2 active quality improvement meetings and participated in quality improvement activities in the preceding 6 months? |
| Confirm | |

| | |
|---|---|
| 32.10 | What proportion of health professionals and support staff caring for neonates and young infants at the health facility had preceding performance appraisal as satisfactory? |
| Confirm | |
| 32.11 | What proportion of all staff at the health facility reported being "highly satisfied" with their job? |
| Confirm | |
| 32.12 | What proportion of all staff at the health facility could identify and report on at least one clinical improvement activity in which they were personally involved in the past six months? |
| Confirm | |
| 32.13 | What proportion of all staff at the health facility are actively considering looking for a new job? |
| Confirm | |

| | |
|---|---|
| **33.** | **Does the health facility have managerial leadership that collectively develops, implements, and monitors appropriate policies and legal entitlements that foster an environment that supports continuous quality improvement?** |
| 33.1 | Does the health facility has a written, up-to-date plan for improving the quality of care and a patient safety programme? |
| Ask | |
| Confirm | Copy |
| 33.2 | Does the health facility have a written, up-to-date leadership structure, with defined roles and responsibilities and lines of accountability for reporting? |
| Ask | |
| Confirm | |
| 33.3 | Does the health facility have a designated quality improvement team and responsible personnel? |
| Ask | |
| Confirm | |
| 33.4 | Does the health facility have a mechanism for regular collection of information on patient and provider satisfaction? |
| Ask | |
| 33.5 | Does the health facility hold at least one monthly meeting to review data, monitor quality improvement performance, make recommendations to address any identified problems, honor those who have performed well and encourage staff who are struggling to improve? |
| Ask | |
| Confirm | |
| 33.6 | Does the health facility hold at least two annual meetings with stakeholders (e.g. the community, service users, partners) to review its performance, identify problems and make recommendations for joint actions to improve quality? |
| Ask | |
| Confirm | |

| | |
|---|---|
| 33.7 | Is there a policy in place for staff to provide feedback to the facility management on quality improvement and their performance? |
| Ask | |
| Confirm | |
| 33.8 | What proportion of all health facility leaders are trained in quality improvement and leading change (use of information, enabling behavior, continuous learning)? |
| Confirm | |
| 33.9 | Is health facility performance monitoring system in place (up to date dashboards or performance charts)? |
| Confirm | |
| 33.10 | What proportion of all health facility leaders who are trained in leadership and management skills? |
| Confirm | |
| 33.11 | Is there an evidence for improved performance of the system (according to the facility dashboard)? |
| Confirm | |
| 33.12 | What proportion of monthly meetings on the quality of care were actually held in the preceding 12 months? |
| Confirm | |
| 33.13 | What proportion of health workers (doctors, nurses) participated in audits of pediatric care at least 4 times per year? |
| Confirm | |

| **34.** | **Are neonates and young infants cared for in a well-maintained, safe, secure physical environment with adequate energy supply and appropriately designed, furnished and decorated to meet their needs, preferences and child's developmental age?** |
| --- | --- |
| 34.1 | Is the health facility structure designed to provide seamless flow and access to dedicated care areas from reception, emergency care area, outpatient and inpatient care areas? |
| Confirm | |
| 34.2 | Does the health facility have separate and dedicated areas for care of neonates and young infants (reception area, triage and emergency care area, outpatient care area, separate neonatal and pediatric care wards)? |
| Ask | |
| Confirm | |
| 34.3 | Does the health facility practice and has facilities that enable rooming-in for mothers and babies together 24 h a day? |
| Ask | |
| 34.4 | Is the health facility adequately maintained, safe, clean, appropriately illuminated, and well-ventilated and allows for privacy for infants and families? |
| Ask | |
| Confirm | |
| 34.5 | Does the health facility have functional, clean and accessible bathroom and toilet facilities and hand washing amenities for use by families? |
| Ask | |
| Confirm | |
| 34.6 | Does The health facility have energy infrastructure (e.g. solar, generator, grid) that can meet all the electricity demands of the facility and associated infrastructure at all times, with a back-up power source? |
| Ask | |
| Confirm | |

| | |
|---|---|
| 34.7 | Does the heath facility have a fuel management plan and a local buffer stock, supported by an adequate budget for all the fuel needs for vehicles, cooking and heating, as relevant and as required, at all times? |
| Ask | |
| Confirm | |
| 34.8 | What is the proportion of times per calendar year when there was a break in power for more than 2 hours a day for one week (the past 7 days)? |
| Ask | |
| 34.9 | What is the proportion of all families who attended the health facility who were satisfied with the environment cleanliness? |
| Confirm | |

| | |
|---|---|
| **35.** | **Are water, sanitation, hand hygiene and waste disposal facilities easily accessible, functional, reliable, safe and sufficient to meet the needs of staff, children and their families?** |
| 35.1 | Does the health facility have written, up-to-date protocols and awareness-raising materials (posters) on cleaning and disinfection, hand hygiene, and operation and maintenance of water, sanitation and hygiene facilities and safe waste management? |
| Ask | |
| Confirm | Copy of the protocol |
| 35.2 | Does the health facility have a functioning source of safe water located on the premises that is adequate to meet all demands for drinking, personal hygiene, medical interventions (formula, oral rehydration salts, nutritional supplements and medicines), cleaning, laundry and cooking for use by staff, children and their families? |
| Ask | |
| 35.3 | Does the health facility have leak-proof, covered, labelled waste bins and impermeable sharps containers available in every treatment area, to allow segregation of waste into four categories: sharps, non-sharps infectious waste, general non-infectious waste? |
| Ask | |
| Confirm | |
| 35.4 | Does the health facility have at least one functioning hand hygiene station per 10 beds, with soap and water or alcohol-based hand rubs, in all wards? |
| Ask | |
| Confirm | |
| 35.5 | Does the health facility have nappies (diapers) available for infants and disposes of them safely, and with a dedicated changing station with hand washing facilities nearby? |
| Ask | |
| Confirm | |
| 35.6 | Does the health facility have sanitation facilities on premises that are usable, appropriately illuminated at night, accessible to people with limited mobility and separated by gender for staff and patients; they include at least one toilet that meets the needs for menstrual hygiene management, with handwashing stations and soap and water (at least 1 latrine per 20 users for inpatient settings)? |
| Ask | |

| | |
|---|---|
| 35.7 | Does the health facility have sufficient trained, competent staff for cleaning, operating and maintaining water, sanitation, hygiene and health care waste facilities, on site when needed and clear descriptions of their responsibilities? |
| Ask | |
| 35.8 | Does the health facility have sufficient funds for rehabilitation, improvement and continuous operation and maintenance of water, sanitation and hygiene infrastructure? |
| Ask | |
| 35.9 | Does the health facility have adequate laundry facilities, including availability of water and detergent? |
| Ask | |
| 35.10 | Do health facility professional and support staff and families receive education and training on good hygiene including regular handwashing after changing nappies, before feeding and after using the toilets? |
| Ask | |
| 35.11 | Does the health facility have a preventive risk plan for managing and improving water, sanitation and hygiene services, including for infection prevention and control? |
| Ask | |
| 35.12 | What is the proportion of days per calendar year when water was not available on the premises? |
| Ask | |
| 35.13 | What is the proportion of days per calendar year when soap or hand disinfectant were not available for hand hygiene? |
| Ask | |
| 35.14 | What proportion of families at the health facility are satisfied with the water, sanitation and energy services? |
| Confirm | |
| 35.15 | What proportion of families who attended the health facility were satisfied with the power and lighting source? |
| Confirm | |
| 35.16 | What proportion of all health care staff at the health facility were satisfied with the water, sanitation and energy services? |
| Confirm | |

| 36. | Are neonates and young infants cared for with appropriate medical equipment? |
|---|---|
| 36.1 | Does the health facility have functioning essential equipment and supplies for routine care and management of complications at all times in all the neonatal and infant care areas |
| Ask | |
| Confirm | |
| 36.2 | Are equipment, their user manual and instructions on how to operate and use equipment available? |
| Ask | |
| Confirm | |
| 36.3 | Does the health facility have a safe, uninterrupted oxygen supply source and delivery supplies (nasal prongs, catheters and face masks), including nasal continuous positive airway pressure, available at all times in children's wards and emergency areas? |
| Ask | |
| Confirm | |
| 36.4 | Does the health facility have basic equipment (x-ray and basic laboratory equipment) for diagnosis and management of common childhood illness and conditions? |
| Ask | |
| Confirm | |
| 36.5 | Does the health facility have a dedicated area in the labor and childbirth area for resuscitation of newborns, which is adequately equipped with a table or resuscitaire, radiant warmer, light and appropriate resuscitation equipment and supplies? |
| Ask | |
| Confirm | |
| 36.6 | Does the health facility have a dedicated ward for admitting sick and unstable small babies? |
| Ask | |
| Confirm | |

| | |
|---|---|
| 36.7 | Does the health facility have a dedicated budget for purchase of essential equipment and its maintenance to optimize child care? |
| Ask | |
| Confirm | Ask for budget during last year or current year |
| 36.8 | What is the proportion of days per calendar year during which one or more of the essential equipment are not available? |
| Confirm | |

| **37.** | **Is adequate stock of medicines and medical supplies available for the routine care and management neonatal and infant illnesses or conditions?** |
|---|---|
| 37.1 | Does the health facility have an on-site pharmacy with a trained pharmacist or dispenser who maintains an essential list of child-friendly medicines and supplies and has an efficient stock management system? |
| Ask | |
| Confirm | |
| 37.2 | Does the health facility have supplies of emergency and pre-referral medicines and supplies available at all times for the management emergency in neonates and infants? |
| Confirm | |
| 37.3 | Does the health facility have supplies of first- and second-line injectable antibiotics and other essential oral medicines available at all times for the management of neonates and young infants? |
| Confirm | |
| 37.4 | Does the health facility have supplies of antenatal corticosteroids (dexamethasone or betamethasone), antibiotics and magnesium sulfate available in sufficient quantities at all times to manage preterm births? |
| Confirm | |
| 37.5 | Does the health facility have essential laboratory supplies (e.g. needles, reagents, specimen bottles) to support routine and emergency management of neonates and young infanst? |
| Confirm | |
| 37.6 | What has been availability of essential life-saving medicines and supplies (oxygen, epinephrine, blood for transfusion, IV fluids, glucose, injectable ampicillin, benzyl penicillin, gentamicin, antiretroviral drugs and vaccines including BCG, polio, DPT, Hep B, and measles) in the past three months? |
| Ask | |
| 37.7 | What is proportion of days per calendar year with stock outs in one or more of the essential drugs? |
| Confirm | |
| 37.8 | Was there no interruption of blood availability in last three months? |
| Confirm | |
| 37.9 | What is the Proportion of days per calendar year when oxygen was not available in the hospital |
| Confirm | |
