# Supplementary material for: Management of possible serious bacterial infection in young infants closer to home when referral is not feasible: Lessons from implementation research in Himachal Pradesh, India
Source: PLoS One. 2020 Dec 22;15(12):e0243724. doi: 10.1371/journal.pone.0243724 (PMC7755274; doi:10.1371/journal.pone.0243724)
Supplement: S2 Questionnaire — (PDF) [file pone.0243724.s011.pdf]

Implementation Research on management of Possible Serious Bacterial Infection in young infants where referral is not feasible through strengthening of existing Home Based newborn care program of the Government of India

ASHA INTERVIEW SCHEDULE

Questions marked with * to be collected for Himachal Pradesh only

Section 1: Identification

1.1	Site ID: Haryana$_1$/Himachal Pradesh$_2$/Maharashtra$_3$/Uttar Pradesh$_4$ [ ]<br>साइट क्रमॉकः हरियाणा$_1$/ हिमाचल प्रदेश$_2$/महाराष्ट्र$_3$/उत्तर प्रदेश$_4$		
1.2	District Name:<br>जनपद का नामः	1.3	Block Name:<br>ब्लाक का नामः
1.4	CHC Name:<br>सामुदायिक स्वास्थ्य केन्द्र का नाम	1.5	PHC Name:<br>प्राथमिक स्वास्थ्य केन्द्र का नामः
1.6	Sub Centre Name:<br>उप केन्द्र का नामः	1.7	Village Name:<br>गाँव का नामः
1.8	ASHA Name:<br>आशा का नामः	1.9	ASHA Mobile Number:<br>आशा का मोबाइल नं0:[ ][ ][ ][ ][ ][ ][ ][ ][ ][ ]

Section 2: Education & Training of ASHA and HBNC visits undertaken by her
अनुभाग–2: प्रशिक्षण, शिक्षा और आशा द्वारा किया गया HBNC भ्रमणः

**Education & Training of ASHA आशा की शिक्षा/प्रशिक्षण**			
2.1	What is your age? (completed years)<br>आपकी उम्र क्या हैं? (पूर्ण वर्षों में)		[ ][ ]
2.2	What is your education? (completed)<br>आपकी शिक्षा क्या हैं? (पूर्ण)	Illiterate$_1$/ Primary$_2$/Middle school$_3$/ High school$_4$/ Intermediate$_5$/ Graduation$_6$/ Post graduation$_7$<br>अनपढ़$_1$/प्राथमिक$_2$/मिडिल$_3$/हाईस्कूल$_4$/ इन्टरमीडिएट$_5$/स्नातक$_6$/परास्नातक$_7$	[ ]
2.3	Have you ever received Home Based New Born Care (*HBNC*) Training?<br>क्या आपने कभी नवजात शिशु गृह भ्रमण पर (*HBNC*) प्रशिक्षण प्राप्त किया है?	Received$_1$ प्राप्त किया$_1$<br>Not received$_2$ नहीं प्राप्त किया$_2$ (go to 2.8) (2.8 पर जायें)	[ ]
2.4	Did you receive HBNC training on Module 6: Skills that Save Lives?<br>क्या आपने HBNC (गृह भ्रमण) मोड्यूल 6: जीवन बचाने के कौशल पर प्रशिक्षण प्राप्त किया हैं?	Yes /हाँ<br>No /नहीं (go to 2.6) (2.6 पर जायें)	[ ]<br>[ ]
2.5	How much duration (in months) has elapsed since you were trained in Module 6: Skills that Save Lives?<br>आपको मोड्यूल 6 का प्रशिक्षण प्राप्त किये हुए कितने महीने हुए हैं?		[ ][ ]<br>(months)<br>(महीने में)
2.6	Did you receive HBNC training on Module 7: Skills that Save Lives with focus on Child Health and Nutrition?<br>क्या अपने HBNC मोड्यूल 7 : बच्चे के स्वास्थ्य और पोषण पर प्रशिक्षण प्राप्त किया है?	Yes /हाँ<br>No /नहीं (go to 2.8) (2.8 पर जायें)	[ ]<br>[ ]
2.7	How much duration (in months) has elapsed since you were trained in Module 7: Skills that Save Lives with focus on Child Health and Nutrition?<br>बच्चे के स्वास्थ्य और पोषण के ध्यान देने के लिए HBNC मोड्यूल 7 पर प्रशिक्षण प्राप्त किये कितने महीने हुए हैं?		[ ][ ]<br>(months)<br>(महीने में)
2.8	Did you receive <u>any other</u> training related to young infant care?<br>क्या आपने इसके अलावा शिशु की देखभाल से सम्बन्धित कोई और प्रशिक्षण प्राप्त किया है।<br>(प्रतिक्रिया पर टिक ☑ का निशान लागायें)	Yes /हाँ<br>No /नहीं (go to 2.10)<br>(2.10 पर जायें)	[ ]<br>[ ]

2.9	Please give details of the training? (year, duration elapsed, what was taught, who conducted etc.)<br>कृपया प्रशिक्षण की जानकारी दें। यह प्रशिक्षण किस वर्ष और कितनी अवधि के लिए कराया गया। साथ ही बताये कि किसने करवाया था और आपको क्या–क्या सिखाया गया?	Year: वर्ष [ ][ ][ ][ ]<br>Duration: अवधि [ ][ ] (Days) (दिन)<br>Who conducted:____________________<br>किसने करवाया<br>What was taught:____________________<br>____________________<br>____________________<br>क्या सिखाया गया____________________<br>____________________<br>____________________
**HBNC visits HBNC नवजात शिशु गृह भ्रमण**		
2.10	Are you aware about the schedule of home visits to be undertaken under HBNC?<br>क्या आप नवजात शिशु HBNC गृह भ्रमण देखभाल के अन्तर्गत तय कार्यक्रम के बारे में जागरूक हैं? (प्रतिक्रिया पर टिक ☑ का निशान लागायें)	Yes /हाँ [ ]<br>No/ नहीं (go to Section 3) (तो अनुभाग 3 पर जायें) [ ]
2.11	Do you undertake HBNC home visits as per this schedule?<br>क्या आप नवजात शिशु HBNC गृह भ्रमण देखभाल तय कार्यक्रम के अनुसार करती है?<br>(प्रतिक्रिया पर टिक ☑ का निशान लागायें)	Yes /हाँ [ ]<br>No/ नहीं (go to Section 3) (अनुभाग 3 पर जायें) [ ]
2.12	How many home visits do you make in case the child is delivered at home?<br>यदि बच्चा घर पर पैदा हुआ है, तो आप कितनी बार HBNC गृह भ्रमण करेगीं?	[ ][ ]<br>(note actual response)<br>(संख्या लिखें)
2.13	How many home visits do you make in case of institutional delivery?<br>यदि बच्चा अस्पताल में पैदा हुआ है, तो आप कितनी बार HBNC गृह भ्रमण करेगीं?	[ ][ ]<br>(note actual response)<br>(संख्या लिखे)

Section 3: Knowledge and awareness level of ASHA
अनुभाग 3ः आशा का ज्ञान और जागरूकता स्तर

**Awareness about Breastfeeding स्तनपान के लिए जागरूकता**		
3.1	When should mother start breastfeeding after delivery?<br>Also describe in detail<br>बच्चा पैदा होने के कितनी देर बाद माँ को स्तनपान कराना चाहिए? विस्तार से वर्णन करें?	[ ][ ] <u>OR या</u> [ ] [ ] <u>OR या</u> [ ][ ]<br>(in minuts) (in hours) (in days)<br>(मिनट में) (घंटो में) (दिनो में)<br>Also write verbatim: विस्तार से लिखे ____________<br>____
3.2	What should be done with colustrum? *(tick all responses given)*<br>मां के पहले गाढ़े दूध (कोलेस्ट्रम) का क्या करना चाहिए? (सभी प्रतिक्रियाओं पर टिक ☑ का निशान लागायें)	• It should be given to young infant [ ]<br>यह शिशु को देना चाहिए<br>• It should NOT be given to young Infant (discarded) यह शिशु को नहीं देना चाहिए (फेंक देना चाहिए) [ ]<br>• Any other specify............... [ ]<br>स्पष्ट करें__________
3.3	What should be given to the young infant during first six months of age? *(tick all responses given)*<br>शिशु को पहले छः माह के दौरान क्या–क्या दिया जाना चाहिए? (सभी प्रतिक्रियाओं पर टिक ☑ का निशान लागायें)	• ONLY breast milk [ ]<br>केवल स्तनपान<br>• Breast milk diluted with water [ ]<br>माँ का दूध पानी के साथ मिलाकर देना चाहिये<br>• Breast milk and honey/water/other liquids [ ]<br>माँ के दूध के अलावा शहद/पानी या कोई अन्य तरल पदार्थ<br>• Breast Milk along with bovine milk/formula milk [ ]<br>माँ के दूध के अलावा गाय, भैंस, बकरी का दूध/डब्बे का दूध
3.4	How frequently should a young infant be breastfed per day?<br>शिशु को एक दिन में कितनी बार स्तनपान कराना चाहिए? (प्रतिक्रिया पर टिक ☑ का निशान लागायें)	• On demand [ ]<br>मांग करने पर<br>• Every 2 hours$_2$ (very frequently) [ ]<br>प्रत्येक 2 घंटे पर (अक्सर)<br>• Greater than or equal to 8 times/day [ ]<br>दिन में आठ बार या उससे अधिक<br>• Don`t know [ ]<br>पता नहीं<br>• Any other specify ________________ [ ]<br>अन्य स्पष्ट करें..

3.5	Do you know the correct position for breastfeeding?<br>क्या आप स्तनपान कराने का सही तरीका जानती है? (प्रतिक्रिया पर टिक ☑ का निशान लागायें)	Yes / हाँ [ ]<br>No / नहीं (Go To 3.7) (3.7 पर जायें) [ ]
3.6	What will you observe when determining the correct position for breastfeeding? *(tick all responses given)*<br>स्तनपान सही तरीके से कराया जा रहा है यह जानने के लिए आपका क्या देखेंगे<br>(सभी प्रतिक्रियाओं पर टिक ☑ का निशान लागायें)	• Infant is held close to mother's body [ ]<br>माँ ने शिशु को करीब से पकड़ा है।<br>• Infant is facing mother approaching breast with nose opposite to nipple [ ]<br>शिशु का मुख माँ के सामने स्तन के करीब हो और उसकी नाक निप्पल के विपरीत हो<br>• Infants whole body is supported by mother, not just neck and shoulders [ ]<br>माँ द्वारा शिशु को पूरे शरीर को सहयोग मिलना चाहिए केवल गले और शरीर को [ ]<br>• Infant`s head and body should be in straight line<br>शिशु का सिर व शरीर एक सीध में होना चाहिए<br>• Any other specify__________________________ [ ]<br>अन्य स्पष्ट करें..
3.7	Do you know the correct attachment for breastfeeding?<br>क्या आप जानती है कि स्तनपान के वक्त मां–बच्चे के बीच सही जुड़ाव का तरीका क्या है? (प्रतिक्रिया पर टिक ☑ का निशान लागायें)	Yes / हाँ [ ]<br>No / नहीं (Go to 3.9) (3.9 पर जायें) [ ]
3.8	What will you observe when determining the correct attachment for breastfeeding? *(tick all responses given)*<br>स्तनपान के लिए मां–बच्चे के बीच में सही जुड़ाव है यह जानने के लिए आप क्या देखेगें (observe) करेंगी?<br>(सभी प्रतिक्रियाओं पर टिक ☑ का निशान लागायें)	• Infant`s lip is attaching with mother`s nipple [ ]<br>शिशु का होठ माँ के स्तन से लगा है<br>• Mother should wait until infant`s mouth is wide open [ ]<br>शिशु का मुंह पूरी तरीके से खुले होने तक मां को इंतजार करना चाहिए<br>• Mother should move her infant quickly onto her breast, aiming infant`s lower lip below the nipple [ ]<br>माँ को अपने नवजात बच्चे को अपने स्तन के पास जल्दी लाना चाहिए ताकि उसका निचला होठ नीचे आयें।<br>• Any other Specify________________________ [ ]<br>अन्य स्पष्ट करें..

**Awareness about temperature of a young infant शिशु के तापमान के बारे में जानकारी**		
3.9	At what temperature is a young infant usually ***hot*** to touch? *(record actual response)* (write 000.0 ˚ F if no response/don't know)<br>सामान्यतः किस तापमान पर शिशु छूने पर गर्म महसूस होता है? (नोट करें) यदि पता नहीं तो 000.0 ˚ F लिखें।	[ ][ ][ ].[ ]˚F
3.10	At what temperature is a young infant usually ***cold*** to touch? *(record actual response)* (write 000.0 ˚ F if no response/don't know)<br>सामान्यतः किस तापमान पर शिशु छूने पर ठण्डा महसूस होता है? (नोट करें) यदि पता नहीं तो 000.0 ˚ F लिखें।	[ ][ ][ ].[ ]˚F
**Awareness about low birth weight (LBW) of a Young Infant**<br>**शिशु के जन्म के समय कम वजन के प्रति जागरूकता**		
3.11	When will you term a young infant low birth weight? *(write 9999 grams if no response)*<br>यह आप कब कहेंगे कि शिशु जन्म के समय कम वजन का है? (यदि कोई प्रतिक्रिया नही तो 9999 लिखे)	[ ][ ][ ][ ] Grams ग्राम

3.12	Do you know the care needed for a LBW young infant?<br>क्या आप जन्म के समय कम वजन के शिशु की जरूरी देखभाल के सम्बध में जानती है?<br>(प्रतिक्रिया पर टिक ☑ का निशान लागायें)	Yes हाँ [ ]<br>No नहीं (Go To 3.14) (3.14 पर जायें) [ ]<br>Dont Know नहीं जानती (Go To 3.14) (3.14 पर जायें) [ ]
3.13	What is the care needed for a LBW young infant? *(tick all responses given)*<br>जन्म के समय कम वजन के शिशु की जरूरी	• Keep the young infant close to the mother [ ]<br>शिशु को माँ के निकट रखे<br>• Exclusively breast feed the baby [ ]<br>शिशु को केवल स्तनपान करायें

	देखभाल क्या है? (सभी प्रतिक्रियाओं पर टिक ☑ का निशान लागायें)	• Maintain temperature<br>तापमान बनाये रखें<br>• Increase the frequency of breastfeeds<br>स्तनपान की आवृत्ति को बढ़ायें<br>• Increase the frequency of home visits<br>गृह भ्रमण की आवृत्ति को बढ़ायें<br>• Weight the young infant on all home visits<br>प्रत्येक गृह भ्रमण पर नवजात शिशु का वजन लें<br>• Any other specify ______________________<br>अन्य स्पष्ट करें..................................	[ ]<br>[ ]<br>[ ]<br>[ ]<br>[ ]
3.14	When will you term a young infant ***Very*** low birth weight (VLBW)? *(write 9999 grams if no response)*<br>यह आप कब कहेंगे कि शिशु जन्म के समय अत्याधिक कम वजन का है? (यदि कोई प्रतिक्रिया नही तो 9999 लिखे)		[ ] [ ] [ ] [ ] grams<br>ग्राम
3.15	Do you know the care needed for VLBW?<br>क्या आप जन्म के समय अत्याधिक कम वजन (VLBW) के शिशु की जरूरी देखभाल के बारे में जानती है?<br>(प्रतिक्रिया पर टिक ☑ का निशान लागायें)	Yes / हाँ<br>No / नही (Go to 3.17) (3.17 पर जायें)<br>Dont Know / नहीं जानती (Go to 3.17) (3.17 पर जायें)	[ ]<br>[ ]<br>[ ]
3.16	What will you advise a mother of VLBW? *(tick all responses given)*<br>आप जन्म के समय अत्याधिक कम वजन वाले शिशु की माँ को क्या सलाह देगी?<br>(सभी प्रतिक्रियाओं पर टिक ☑ का निशान लागायें)	• Home based care<br>गृह आधारित देखभाल<br>• Refer the baby to a health facility<br>शिशु को स्वास्थ सुविधा केन्द्र पर सन्दर्भित करेंगी।<br>• Maintain temperature<br>तापमान बनाये रखने की सलाह देंगी।<br>• Feed, if baby is able to suck<br>यदि शिशु चूसने में समर्थ है तो आप स्तनपान कराने की सलाह देंगी<br>• Any other specify______________________<br>अन्य स्पष्ट करें..	[ ]<br>[ ]<br>[ ]<br>[ ]<br>[ ]
**Awareness on identification of local infections in young infant**<br>**शिशु में स्थानीय संक्रमण की पहचान के बारे में जानकारी**			
3.17	Do you know the signs of skin infection in young infants?<br>क्या आप शिशु की त्वचा में संक्रमण के लक्षणों के बारे में जानती है?<br>(प्रतिक्रिया पर टिक ☑ का निशान लागायें)	Yes हाँ<br>No / नहीं (Go to 3.19) (3.19 पर जायें)<br>Don`t Know/नहीं जानती (Go to 3.19) (3.19 पर जायें)	[ ]<br>[ ]<br>[ ]
3.18	How will you look for signs of skin infection? *(tick all responses given)*<br>आप शिशु में त्वचा संक्रमण के लक्षणों को कैसे देखेंगी?<br>(सभी प्रतिक्रियाओं पर टिक ☑ का निशान लागायें)	• Look at umbilicus for redness/pus discharge<br>लालिमा / मवाद के लिए नाल पर देखे<br>• Turn the baby back (upside down) and see<br>शिशु को पलटे (उल्टा) और देखे<br>• Look behind the ears<br>कान के पीछे देखे<br>• Inspect for skin pustules<br>त्वचा पर मवाद वाले दानो की जाँच करें<br>• Any other specify ___________________________<br>अन्य स्पष्ट करें..	[ ]<br>[ ]<br>[ ]<br>[ ]<br>[ ]
3.19	Do you know the signs of oral infection?<br>क्या आप शिशु के मुंह में संक्रमण के लक्षणों के बारे में जानती है?<br>(प्रतिक्रिया पर टिक ☑ का निशान लागायें)	Yes हाँ<br>No / नहीं (Go to 3.21) (3.21 पर जायें)<br>Don`t Know/नहीं जानती (Go to 3.21) (3.21 पर जायें)	[ ]<br>[ ]<br>[ ]
3.20	How will you look for signs of oral infection? *(tick all responses given)*<br>आप शिशु में मुख संक्रमण के लक्षणों को कैसे देखेंगी?<br>(सभी प्रतिक्रियाओं पर टिक ☑ का निशान लागायें)	Check the oral cavity for presence of any redness or white patch<br>मुख गुहा में लालिमा / सफेद चकत्तो को जाँचे<br>Any other specify ____________________________<br>अन्य स्पष्ट करें..	[ ]<br>[ ]
3.21	Do you know how to take care of umbilicus of young infant?<br>क्या आप शिशु की नाल की देखभाल के बारे में जानती है?<br>(प्रतिक्रिया पर टिक ☑ का निशान लागायें)	Yes / हाँ<br>No / नहीं (Go to 3.23) (3.23 पर जायें)<br>Don`t Know / नहीं जानती (Go to 3.23) (3.23 पर जायें)	[ ]<br>[ ]<br>[ ]

3.22	How to take care of the umbilicus? *(tick all responses given)*<br>नाल की देखभाल के बारे में आप क्या सलाह देंगी ?<br>(सभी प्रतिक्रियाओं पर टिक ☑ का निशान लागायें)	• Keep the umbilicus dry<br>नाल को सूखा रखे<br>• No application of any type of oil or massage<br>नाल पर किसी प्रकार का तेल न लागाए/ मालिश न करें<br>• Look for any type of umbilical discharge<br>नाल से किसी प्रकार के रिसाव का निरीक्षण<br>• White/yellow coloured boil(s) over umbilicus<br>नाल के आस–पास सफेद/पीले मवाद वाले दानों का निरीक्षण<br>• Any other specify ___________________________<br>अन्य स्पष्ट करें..	[ ]<br>[ ]<br>[ ]<br>[ ]<br>[ ]
3.23	Do you know how to identify infections related to eyes?<br>क्या आप आँख से सम्बन्धित संक्रमण की पहचान करना जानती है? (प्रतिक्रिया पर टिक ☑ का निशान लागायें)	Yes/हाँ<br>No/नहीं (Go to 3.25) (3.25 पर जायें)<br>Don`t Know/नहीं जानती (Go to 3.25) (3.25 पर जायें)	[ ]<br>[ ]<br>[ ]
3.24	How will you look for signs of infections related to eyes? *(tick all responses given)*<br>आप आँखों से सम्बन्धित संक्रमण के लक्षणों की पहचान कैसे करेगी? (सभी प्रतिक्रियाओं पर टिक ☑ का निशान लागायें)	Swelling or redness in eyes<br>आँखों में लालिमा या सूजन<br>Increase in tears<br>ज्यादा आंसू बहना<br>Any other specify____________________________<br>अन्य स्पष्ट करें..	[ ]<br>[ ]<br>[ ]
**Awareness on danger signs in young infant**<br>**शिशु में खतरों के लक्षणों के प्रति जागरूकता**			
3.25	Do you know how to look for danger signs in young infants?<br>क्या आप शिशु में खतरों के लक्षणों को देखना जानती है?<br>(प्रतिक्रिया पर टिक ☑ का निशान लागायें)	Yes/हाँ<br>No/नहीं (Go to section 4) (अनुभाग 4 पर जायें)<br>Don`t Know/नहीं जानती (Go to section 4) (अनुभाग 4 पर जायें)	[ ]<br>[ ]<br>[ ]
3.26	How will you look for danger signs in young infants? *(tick all responses given)*<br>आप कैसे देखेंगी कि शिशु में खतरों के लक्षण मौजूद है? (सभी प्रतिक्रियाओं पर टिक ☑ का निशान लागायें)	• Infant not able to feed since birth/stopped feeding well or not feeding at all<br>जन्म से स्तनपान करने में असमर्थ/ स्तनपान करना कम कर दिया/बंद कर दिया<br>• Convulsions/ Seizures<br>झटके/दौरे<br>• Lower Chest Movements (Severe Chest Indrawing)<br>छाती धंसना<br>• Hot to touch<br>छूने पर गर्म महसूस होना<br>• Feels cold to touch<br>छूने पर ठंडा महसूस होना<br>• Movement only when stimulated<br>केवल उद्दीपर पर हिलना–डुलना<br>• Fast Breathing (breaths 60/minute or more)<br>तेज साँस चलना (60 साँस/मिनट या अधिक)<br>• Jaundice<br>पीलिया<br>• Diarrhoea<br>डायरिया (दस्त)<br>• Pustules (10 or more) or one large focus of infection<br>मवाद वाले दाने (10 या अधिक) या संक्रमण का बड़ा केन्द्र (फोड़ा)<br>• Any other specify__________________<br>अन्य स्पष्ट करें...	[ ]<br>[ ]<br>[ ]<br>[ ]<br>[ ]<br>[ ]<br>[ ]<br>[ ]<br>[ ]<br>[ ]<br>[ ]

Section 4: Items Available with ASHA during Home Visit for HBNC
अनुभाग4: HBNC के लिए आशा द्वारा गृह भ्रमण के दौरान उपलब्ध सामाग्री
(नोटःप्रतिक्रिया पर टिक ☑ का निशान लागायें)

4.1	HBNC home visit forms (`*Prapatra*` 1)<br>HBNC गृह भ्रमण प्रपत्र (प्रपत्र 1)	Yes /हाँ [ ]<br>No / नहीं [ ]	
4.2	HBNC kit (Bag)<br>HBNC किट (थैला)	Yes /हाँ [ ]<br>No / नहीं (go to next Section) (दूसरे खंण्ड में जाये) [ ]	
4.3	Baby Blanket<br>बच्चे का कम्बल	Yes / हाँ [ ]<br>No / नहीं [ ]	
4.4	Stainless steel spoon/paladai<br>जंग ना लगने वाला चम्मच / पल्लडी	Yes / हाँ [ ]<br>No / नहीं [ ]	
**Equipment उपकरण**			
		**Available**<br>**उपलब्ध**	**Functional**<br>**कार्यात्मक**
4.5	Weighing scale with sling<br>वजन मशीन, गोफन सहित	Yes / हाँ [ ]<br>No / नहीं [ ]	Yes / हाँ [ ]<br>No / नहीं [ ]
4.6	Digital thermometer<br>डिजिटल थर्मामीटर	Yes / हाँ [ ]<br>No / नहीं [ ]	Yes / हाँ [ ]<br>No / नहीं [ ]
4.7	Digital Watch/Timer Device<br>डिजिटल घड़ी / टाइमर उपकरण	Yes / हाँ [ ]<br>No / नहीं [ ]	Yes / हाँ [ ]<br>No / नहीं [ ]
**Medications दवायें**			
4.8	Gentian violet paint (0.5% and 0.25%)<br>जेन्शियन वायलट पेन्ट (0.5% एवं 0.25%)	Yes / हाँ [ ]<br>No / नहीं [ ]	
4.9	Syrup paracetamol<br>पैरासिटामॉल सिरप	Yes / हाँ [ ]<br>No / नहीं [ ]	
4.10	Syrup/Tab Amoxycillin<br>सिरप / ऐमाक्सीसीलिन टैबलेट	Yes / हाँ [ ]<br>No / नहीं [ ]	
**Consumables उपभोग हेतु वस्तुऐं**			
4.11	Cotton<br>रूई	Yes / हाँ [ ]<br>No / नहीं [ ]	
4.12	Soap and soap case<br>साबुन एवं साबुनदानी	Yes / हाँ [ ]<br>No / नहीं [ ]	
4.13	Gauze<br>पट्टी	Yes / हाँ [ ]<br>No / नहीं [ ]	

Section 5: Observation of skills of ASHA during home visits
अनुभाग 5: गृह भ्रमण के दौरान आशा के कौशल की निगरानी
(नोटःप्रतिक्रिया पर टिक ☑ का निशान लागायें)
***HBNC Home visit not observed*... [ ]**

5.A)Mother (MCTS)/RCH ID No.<br>माता का (MCTS)/RCH पहचान नं0 (if not available fill `99999999999999999`)	[ ][ ][ ][ ][ ][ ][ ][ ][ ][ ][ ][ ][ ][ ][ ][ ][ ]
5.B)CHILD (MCTS)/RCH ID No.<br>बच्चे का (MCTS)/RCH पहचान नं0 (if not allotted yet fill `99999999999999999`)	[ ][ ][ ][ ][ ][ ][ ][ ][ ][ ][ ][ ][ ][ ][ ][ ][ ]

**S No.**	**Skills to observe**<br>जो कौशल देखने हैं	**Response**<br>प्रतिक्रिया
5.1	Did ASHA greet the family?<br>क्या आशा ने परिवार का अभिवादन किया?	Yes / हाँ [ ]<br>No / नहीं [ ]
5.2	Did ASHA explain to mother purpose of home visit in a gentle voice and respectful manner?<br>क्या आशा ने गृह भ्रमण का उद्देश्य माँ को सौम्य एवं सम्मानित तरीके से समझाया?	Yes / हाँ [ ]<br>No / नहीं [ ]
5.3	Did ASHA wash her hands with soap and water before examining the young infant? *(As per Annexure 7 of Module 6)*<br>क्या आशा ने शिशु की जाँच करने से पहले अपने हाथ साबुन और पानी से धो कर साफ किया? *(एनेक्सर 7 के माड्यूल 6 के अनुसार)*	Yes / हाँ [ ]<br>No / नहीं [ ]

5.4	Did ASHA count the breath correctly using a digital watch?<br>क्या आशा ने डिजिटल घड़ी का उपयोग करते हुऐ बच्चे के सांस की गिनती की?	Yes / हाँ<br>No / नहीं<br>Not applicable as watch unavailable/not functional अनुपलब्ध या कार्यशील न होने के कारण लागू नहीं हो सकता	[ ]<br>[ ]<br>[ ]
5.6	Did ASHA weigh the Young Infant correctly? *(As per Annexure 9 of Module 6)*<br>क्या आशा ने बच्चे का वजन सही तरीके से मापा *(एनेक्सर 9 के माड्यूल 6 के अनुसार)*	Yes / हाँ<br>If yes note weight taken<br>No / नहीं<br>Not applicable as weigning scale with sling unavailable/not functional<br>वजन मशीन की अनुपलब्ध या कार्यशील न होने के कारण लागू नहीं हो सकता	[ ]<br>[ ] [ ][ ][ ] grms<br>[ ]<br>[ ]
5.7	Did ASHA measure temperature of the young infant correctly? *(As per Annexure 8 of Module 6)*<br>क्या आशा ने शिशु का तापमान सही तरीके से नापा *(एनेक्सर 8 के माड्यूल 6 के अनुसार)*	Yes / हाँ<br>If yes note temperature taken<br>No / नहीं<br>Not applicable as thermometer unavailable/not functional<br>थर्मामीटर अनुपलब्ध या कार्यशील न होने के कारण लागू नहीं हो सकता	[ ]<br>[ ][ ] [ ].[ ] ˚ F<br>[ ]<br>[ ]
5.8	Did ASHA remove clothes of young infant for examination of rash?<br>क्या आशा ने दाने की जांच के दौरान शिशु के कपड़े हटाये?	Yes / हाँ<br>No / नहीं	[ ]<br>[ ]
5.9	Did ASHA remove clothes of the young infant for examination of umbilical discharge?<br>क्या आशा ने नाभि / नाल में से रिसाव की जांच के दौरान शिशु के कपड़े हटाये?	Yes / हाँ<br>No / नहीं	[ ]<br>[ ]
5.10	Did ASHA remove clothes of the young infant for examination of skin pustules/boils?<br>क्या आशा ने मवाद के दानों / फोड़े की जांच के दौरान शिशु के कपड़े हटायें?	Yes / हाँ<br>No / नहीं	[ ]<br>[ ]
5.11	Did ASHA remove clothes of the young infant for examination of lump at lower back (meningomyelocoele)?<br>क्या आशा ने पीठ के निचले हिस्से में किसी गांठ की जांच के दौरान शिशु के कपड़े हटाये?	Yes / हाँ<br>No / नहीं	[ ]<br>[ ]
5.12	Did ASHA counsel mother on position of breastfeeding?<br>क्या आशा ने माँ को स्तनपान कराने की सही स्थिति के बारे में सलाह दी?	Yes / हाँ<br>No / नहीं (go to 5.14) (5.14 पर जायें)	[ ]<br>[ ]
5.13	What did she counsel mother on position of breastfeeding? *(tick all responses given)*<br>आशा ने माँ को स्तनपान कराने की सही स्थिति समझाने के लिए क्या बताया? (सभी प्रतिक्रियाओं पर टिक ☑ का निशान लागायें)	• Infant should be held close to mother's body<br>शिशु को माँ के शरीर के नजदीक रखें<br>• Infant`s face should approach the breast with nose opposite to nipple<br>शिशु का मुँह स्तन के नजदीक हो एवं नांक निप्पल के पास रखें<br>• Infant`s whole body should be supported, not just neck and shoulders<br>सिर्फ गर्दन और कन्धा ही नहीं बल्कि शिशु का पूरा शरीर संभालना चाहिए<br>• Infant`s head and body should be in straight line<br>शिशु का सिर एवं शरीर एक सीधी रेखा में होना चाहिए<br>• Any other specify ___________________<br>अन्य स्पष्ट करे...	[ ]<br>[ ]<br>[ ]<br>[ ]<br>[ ]
5.14	Did ASHA counsel mother on attachment for breastfeeding?<br>क्या आशा ने माँ को स्तनपान के दौरान मां–बच्चे में जुड़ाव का सही तरीका समझाया?	Yes / हाँ<br>No / नहीं (goto 5.16) (5.16 पर जायें)	[ ]<br>[ ]

5.15	What did she counsel on attachment for breastfeeding? *(tick all responses given)*<br>आशा ने माँ को स्तनपान के दौरान मां–बच्चे के जुड़ाव के लिए क्या सलाह दी? (सभी प्रतिक्रियाओं पर टिक ☑ का निशान लागायें)	• Infant`s lip is attaching with mother`s nipple<br>नवजात के होंठ माँ के निप्पल को स्पर्श करना चाहिए<br>• Mother should wait until her infant`s mouth is wide open<br>माँ को नवजात के मुँह को पूरा खुलने का इंतजार करना चाहिए<br>• Mother should move her infant quickly onto her breast, aiming infant`s lower lip below the nipple<br>माँ को नवजात को अपने स्तन पर इस प्रकार लगाना चाहिए कि नवजात के होंठ का निचला हिस्सा निप्पल के नीचे रहे<br>• Any other specify………………<br>अन्य स्पष्ट करे....................................	[ ]<br>[ ]<br>[ ]<br>[ ]
5.16	Did ASHA counsel the mother to maintain temperature of young infant?<br>क्या आशा ने माँ को नवजात के तापमान नियंत्रण के बारे में सलाह दी?	Yes / हाँ<br>No / नहीं (goto 5.18) (5.18 पर जायें)	[ ]<br>[ ]
5.17	What did ASHA counsel on temperature maintenance?<br>आशा ने माँ को तापमान नियंत्रण के बारे में क्या–क्या सलाह दी? (सभी प्रतिक्रियाओं पर टिक ☑ का निशान लागायें)	• Infant should be held close to the mother<br>नवजात को माँ के शरीर के चिपका कर रखना चाहिए<br>• Kangaroo Mother Care<br>कंगारू मदर केयर (के एम सी) के बारे में<br>• If the mother is not close, keep warm water filled bottle wrapped in cloth near the baby blanket<br>यदि माँ पास में न हो, तो गर्म पानी की बोतल को कपड़े में लपेट कर बच्चे के कम्बल के पास रखना चाहिए<br>• Keep the room warm (at around 25 °C) in cold weather and at air cooled in hot weather<br>सर्दी के मौसम में कमरे को लगभग 25°C तापमान पर नियंत्रित रखना चाहिए तथा गर्मियों में कमरा हवादार रखना चाहिए<br>• Avoid bathing<br>स्नान न कराये<br>• Keep the infant well clothed including hat and socks<br>नवजात को अच्छे से कपड़े पहनाये, मोजे और टोपी के साथ<br>• Change clothes when wet.<br>बच्चे के कपड़े गीले होने पर बदल दें<br>• Any other specify _____________<br>अन्य स्पष्ट करे...	[ ]<br>[ ]<br>[ ]<br>[ ]<br>[ ]<br>[ ]<br>[ ]<br>[ ]
5.18	Did ASHA ask/look/feel for danger signs in young infant?<br>क्या आशा ने नवजात शिशु में खतरे के लक्षणों को पूछा / देखा / निरीक्षण किया?	Yes / हाँ<br>No / नहीं (goto 5.20) (5.20 पर जायें)	[ ]<br>[ ]

5.19	What danger signs did ASHA look for/ask?<br>आशा ने नवजात शिशु में खतरे के क्या–क्या लक्षण देखे/पूछें?<br>(सभी प्रतिक्रियाओं पर टिक ☑ का निशान लागायें)	• Infant is not able to feed since birth or has stopped feeding well or not feeding at all<br>नवजात जन्म से ही स्तनपान करने में असमर्थ है या अपेक्षा से कम स्तनपान कर रहा है या बिलकुल स्तनपान नहीं कर रहा है	[ ]
		• Convulsions/Seizures<br>शरीर ऐठना/झटके आना	[ ]
		• Lower Chest Movements (Severe Chest Indrawing)<br>छाती के निचले हिस्से का धँसना	[ ]
		• Hot to touch<br>छूने पर गर्म महसूस होना	[ ]
		• Feels cold to touch<br>छूने पर ठंडा महसूस होने का	[ ]
		• Infant moves only when stimulated<br>नवजात का सिर्फ छूने पर हिलना–डुलना	[ ]
		• Fast Breathing (breaths 60/minute or more)<br>तेज सांस चलना (प्रति मिनट 60 या उससे अधिक)	[ ]
		• Jaundice<br>पीलिया	[ ]
		• Diarrhoea<br>डायरिया	[ ]
		• Pustules (10 or more) or one large focus of infection<br>10 या अधिक मवाद युक्त छोटे पीले दाने या एक बड़ा फोड़ा	[ ]
5.20	Was the baby having any danger signs?<br>क्या नवजात में खतरे के कोई लक्षण थे?	Yes/हाँ	[ ]
		No/नहीं (goto section 6) (अनुभाग 6 पर जायें)	[ ]
5.21	What information did ASHA give to parents or what actions she took to manage young infant with danger sign? *(Tick all actions/information observed)*<br>नवजात में खतरे के लक्षण मौजूद होने पर आशा ने अभिभावको को क्या सलाह दी एवं उसके प्रबन्धन हेतु क्या उपाय बताया? (सभी प्रतिक्रियाओं पर टिक ☑ का निशान लागायें)	• Did nothing<br>कुछ नहीं किया	[ ]
		• Explain to the caregivers that the child is sick<br>माता–पिता को बताया कि उनका बच्चा बीमार है	[ ]
		• Convince the caregivers to seek qualified medical care<br>माता–पिता को कुशल चिकित्सीय परामर्श लेने हेतु संतुष्ट किया	[ ]
		• Call 108/102 ambulance<br>108/102 एम्बुलेन्स को बुलाया	[ ]
		• Refer the child<br>बच्चे को संदर्भित किया	[ ]
		• Consult/inform the ANM<br>ए0एन0एम0 से परामर्श लेने की सलाह दी	[ ]
		• Any other specify ______________<br>अन्य स्पष्ट करे.------------------------------------	[ ]

अनुभाग 6: गृह भ्रमण के दौरान **HBNC** सलाह देते समय आशा द्वारा किसी प्रकार की कठिनाई का सामना करना पड़ा

6.1. Problems from community: (Probe for socio-cultural barriers)
6.1. समुदाय से समस्याएं

6.2. Problems from family of Index child: (Probe for socio cultural barrier etc.)
6.2 चिन्हित बच्चे के परिवार से समस्याएं

6.3. **Problems from Health Department:** (Probe for Mistrust of public health services by family, Non availablility of medicine etc.) स्वास्थ्य विभाग से समस्याएं

6.4. Any other Problem अन्य कोई समस्याएं

6.5*. Suggestions on what can be done to make her work better/easier *उसके काम को और बेहतर/आसान बनाने के लिए क्या कर सकते हैं उसके लिए सुझाव*

Interview Details		
1.	Interviewer Name<br>Interviewer Staff Code	 [ ] [ ]
2.	Signature Interviewer	
3.	Date of interview (dd/mm/yy)	[ ] [ ]/[ ] [ ]/[ ] [ ][ ] [ ]
4.	Supervisor Name<br>Supervisor Staff Code	 [ ] [ ]
5.	Signature of Supervisor	
6.	Date of validation (dd/mm/yy)	[ ] [ ]/[ ] [ ]/[ ] [ ][ ] [ ]
7.	Data Entry Operator Name	
9.	Date of Data Entry	[ ] [ ]/[ ] [ ]/[ ] [ ][ ] [ ]

Annexure

Section 7:

7.1: List of children who died before 1 year of age in the last two years in your area.

आपके क्षेत्र में पिछले 2 वर्षों में 1 वर्ष से कम आयु के बच्चों की मृत्यु की सूची

Child number बच्चे का क्रम	Name of Village and Father Mother गांव का नाम और पिता / माता का नाम	When did the child die (year) बच्चे की मृत्यु कब हुई(साल में)	Place of delivery(Home[1] /Private Institution[2]/ Govt. Institution[3]/ /Transit[4]/ Not known[8]) प्रसव का स्थान (घर [1] / निजी संस्थान[2]/ सरकारी संस्थान [3] / रास्ते में [4]/ ज्ञात नहीं[8])	Immunization Status (Fully Immunized for age [1]/Not Immunized[2] /Not Known[8]) टीकाकरण स्थिति (पूरी तरह से उम्र के लिए प्रतिरक्षित[1] / प्रतिरक्षित नहीं[2] /ज्ञात नहीं[8])	Age (at death) please specify the age in months आयु (मृत्यु पर) कृपया महीनों में उम्र लिखें	Place of death (Home[1]/ Private Institution[2]/ Govt. Institution[3]/ /Transit[4] / Do not know[8]) अगर मृत , मृत्यु का स्थान (घर [1] / निजी संस्थान[2]/ सरकारी संस्थान [3] / रास्ते में [4] / नही पता[8])	Cause of death if known मृत्यु का कारण यदि ज्ञात हो	If died following an illness, after how many days यदि बीमारी के बाद मृत्यु हो गई है, तो कितने दिन बाद
1								
2								
3								
4								
5								

7.2 Any other information shared by the family regarding the child's death:

बच्चे की मृत्यु के बारे में परिवार द्‌वारा साझा की गई कोई अन्य जानकारी

____

____

____

____

____

____

____

____

____

____

____

____

____

____

____

____

____

____

____

____

____
