# Supplementary material for: Management of possible serious bacterial infection in young infants closer to home when referral is not feasible: Lessons from implementation research in Himachal Pradesh, India
Source: PLoS One. 2020 Dec 22;15(12):e0243724. doi: 10.1371/journal.pone.0243724 (PMC7755274; doi:10.1371/journal.pone.0243724)
Supplement: S3 Questionnaire — (PDF) [file pone.0243724.s012.pdf]

Country ID: [1] Site ID: [ ] Form Number: [I] [ ] [ ] [ ] **{Baseline Survey Form 3}**

Implementation Research on management of Possible Serious Bacterial Infection in young infants where referral is not feasible through strengthening of existing Home Based newborn care program of the Government of India

IN-DEPTH INTERVIEW OF MOTHERS

(Highlighted section not be asked directly)
Questions marked with * to be collected for Himachal Pradesh only

Section 1: Identification

1.1	Site ID: [ ]<br>Haryana$_1$/Himachal Pradesh$_2$/ Maharashtra$_3$/Uttar Pradesh$_4$	1.4	District Name: _________________
		1.5	Block Name: __________________
		1.6	Sub Centre Name: ______________
1.2	Village Name: _____________________	1.7	ASHA Name: _________________
1.3	ASHA Mobile Number: ___________________		

Section 2: Screening & Eligibility स्क्रीनिंग और पात्रता

**Screening Question**<br>Can young infants (from birth upto 2 months of age) get a serious illness?<br>क्या शिशुओं को गम्भीर बीमारी हो सकती है। (जन्म से 2 माह की आयु तक)	Yes$_1$/No$_2$/Don't know$_3$<br>हाँ$_1$/ नही$_2$/ पता नहीं$_3$

If the response to screening question is **"Yes"**; confirm eligibility below<br>यदि स्क्रीनिंग प्रश्न का उत्तर हाँ है, तो नीचे पात्रता की पुष्टि करें				
**<u>Inclusion Criteria</u> इन्क्लूजन क्राइटेरिया**			**<u>Exclusion Criteria</u> एक्सक्लूजन क्राइटेरिया**	
1. Mother of a young infant ≤ 2 months of age<br>02 माह से कम आयु के बच्चे की माँ	Yes$_1$ / No$_2$	[ ]	1. Any mother who's infant child has been expired recently.<br>ऐसी मां जिसके बच्चे की हाल ही में मृत्यु हुई है।	Yes$_1$/हां$_1$ [ ]<br>No$_2$/नही$_2$
2. Residing in this area since ≥ 6 months<br>इस क्षेत्र में 6 माह से अधिक समय से निवास करने वाली महिला	Yes$_1$ / No$_2$	[ ]		
3. Consents verbally for interview<br>साक्षात्कार के लिए मौखिक अनुमति	Yes$_1$ / No$_2$	[ ]		
**Note:** Include those with Yes to ALL inclusion criteria and No to exclusion criteria **(If eligible, proceed below)**<br>नोट :- जिन्होनें सभी इंनक्लूजिंग मानक का उत्तर हां में दिया है उन्हें शामिल करें एवं जिन्होंनें एक भी एक्सक्लूजिंग मानक का उत्तर हां में दिया है उन्हें ना शामिल करें। (यदि पात्रता है तो नीचे आगे बढ़े)				Yes$_1$/हां$_1$ [ ]<br>No$_2$/नही$_2$

Section 3: Characteristics of Parents and Index Child

3.1	MCTS/RCH ID of Mother :	Available$_1$ / Not Available Today$_2$ [ ]
3.2	MCTS/RCH ID of Mother:	[ ][ ][ ][ ][ ][ ][ ][ ][ ][ ][ ][ ][ ][ ][ ][ ][ ]
3.3	MCTS/RCH ID of Child :	Available$_1$ / Not Available Today$_2$ [ ]
3.4	MCTS/RCH ID of Child :	[ ][ ][ ][ ][ ][ ][ ][ ][ ][ ][ ][ ][ ][ ][ ][ ][ ]
3.5	Age of Mother	[ ][ ] (in completed years)
3.6	Education of Mother (completed)	Illiterate$_1$ / Primary level$_2$ / Middle school$_3$ / Highschool$_4$ / Intermediate$_5$/Graduate$_6$ / Postgraduate$_7$ / Professional$_8$ [ ]<br>अशिक्षित$_1$/प्राथमिक$_2$/माध्यमिक$_3$/हाईस्कूल$_4$/इण्टरमीडिएट$_5$/स्नातक$_6$/परास्नातक$_7$/ व्यवसायिक$_8$
3.7	Occupation of Mother	Housewife$_1$ / Un-skilled worker$_2$ / Skilled worker$_3$ / Farmer$_4$ / Shop owner$_5$ / Clerical$_6$ / Professional$_7$ [ ]<br>गृहणी$_1$/अकुशल कारीगर$_2$/कुशल कारीगर$_3$/ किसान$_4$/दुकानदार$_5$/लिपिक$_6$/व्यवसायिक$_7$

3.7.1	***If working outside home:***	***Number of hours spent away from home.***	***[ ]***
3.8	Age of Father (in completed years)		[ ] [ ] years
3.9	Education of Father (completed)	Illiterate$_1$ / Primary level$_2$ / Middle school$_3$ / Highschool$_4$ / Intermediate$_5$ / Graduate$_6$ / Postgraduate$_7$ / Professional$_8$<br>अशिक्षित$_1$/प्राथमिक$_2$/माध्यमिक$_3$/हाईस्कूल$_4$/इण्टरमीडिएट$_5$/स्नातक$_6$/परास्नातक$_7$/ व्यवसायिक$_8$	[ ]
3.10	Occupation of Father	Unemployed$_1$ / Un-skilled worker$_2$ / Skilled worker$_3$ / Farmer$_4$ / Shop owner$_5$ / Clerical$_6$ / Professional$_7$<br>बेरोजगार$_1$/अकुशल कारीगर$_2$/कुशल कारीगर$_3$/ किसान$_4$/दुकानदार$_5$/लिपिक$_6$/व्यवसायिक$_7$	[ ]
3.11	Age of index child (< 1 year)		Days [ ] [ ]
3.12	Gender	Male$_1$ / Female$_2$	[ ]
3.13	Number of Living Sibling		[ ] [ ]
3.14	Birth order		[ ] [ ]

Section 4: In-depth Interview गहन साक्षात्कार

Q.1 Can you tell what are the symptoms of serious illness in young infants?

क्या आप बता सकते हैं शिशुओं में गम्भीर बीमारी के लक्षण क्या हैं?

Probe<br>पूछें	Write verbatim<br>कथन को लिखें	(Coding)<br>*(For office use only)*<br>कोड (आफिस कार्य हेत)
a. Feeding related symptoms<br>आहार सम्बंधी लक्षण		
b. Body temperature<br>शरीर का तापमान		
c. Movement of infants (voluntarily or when stimulated)<br>शिशुओं की शारीरिक गतिविधि (स्वेच्छा से या हिलाने डुलाने पर)		
d. Breathing (fast/chest indrawing)<br>सांस (तेज/पसली धंसना)		
e. Abnormal movements /Seizures/ Convulsions<br>असमान्य गतिविधि/झटके/ऐठन		
f. Change in skin color<br>त्वचा का रंग में बदलाव		
g. Any other<br>अन्य		

Q.2 Are any of the symptoms told by you more significant:

क्या आपके द्वारा बताये गये लक्षणों में से कोई ज्यादा महत्वपूर्ण है:

Probe<br>पूछें	Write verbatim<br>कथन को लिखें	(Coding)<br>*(For office use only)*<br>केवल कार्यालय प्रयोग हेतु
a. In first 6 days?<br>प्रथम 6 दिनों में		
b. In first 28 days?<br>प्रथम 28 दिनों में		
c. In 28-59 days?<br>28 से 59 दिनों के अंदर		
d. Among those children who are small at birth (< 2.5 kg)?<br>जन्म से कम वजन वाले बच्चों में<br>(< 2.5 kg)		
e. Among those children who are born in hospital/home?<br>अस्पताल/घर पर जन्म लेने वाले बच्चों में		
f. Among those children who are born before 37 weeks of gestation?<br>गर्भधारण के 37 हफ्ते पूर्व जन्म लेने वाले बच्चों में		
g. Among those children who are not exclusively breast fed?<br>सिर्फ स्तनपान न करने वाले बच्चों में		

Q. 3 **Is yellow discoloration of skin a sign to seek medical care? Explain.** Yes/No/Don't know (Probe: Within 24 hours/when palms and soles are yellow)
क्या त्वचा का पीला पड़ना चिकित्सीय देखभाल की जरूरत का लक्षण है। स्पष्ट करें ? हाँ/नहीं/नहीं पता (पूछें: 24 घण्टें के अंदर जब हथेली/तलवे का रंग पीला हो)

(Write verbatim): ____

कथन लिखें:..

Q.4 **Information on stools in newborns/ young Infants:**
नवजात शिशु/शिशुओं में मल की जानकारी:

Probe<br>पूछें	Write verbatim<br>कथन लिखें	*For office use only*<br>कार्यालय प्रयोग हेतु
a. How many times do young infants pass stools/day (in relation with feed)?<br>दो माह से छोटे बच्चे पूरे दिन में कितनी बार मल करते हैं।		
b. Is it related to feeding<br>क्या यह आहार से सम्बंधित है?		
c. What is the consistency of stools of breast feeding infants?<br>मां के दूध का सेवन करने वाले बच्चो में किस प्रकार का मल होता है?		
d. Is it different in those not breastfed?<br>क्या यह स्तनपान न करने वाले बच्चों से अलग है?		
e. When will you seek care for diarrhea in an infant?<br>डायरिया से ग्रसित दो माह से छोटे बच्चे को आप अस्पताल कब दिखाने ले जाएगी?<br>(Probe: consistency of stools, frequency of stools, movement of infant, sunken eyes, loose skin etc.)<br>(पूछेः मल का प्रकार/मल की आवृत्ति, नवजात की गतिविधियां, धंसी हुई आंखे, ढ़ीली त्वचा आदि)		

Q.5. Can infants get skin/mouth/eyes infections?
क्या 2 माह से छोटे बच्चों में त्वचा/मुँह/आँखों से सम्बन्धित संक्रमण हो सकता है?

Probe<br>पूछें	Write verbatim<br>कथन लिखें	*For office use only*<br>कार्यालय प्रयोग हेतु
Skin Related<br>त्वचा सम्बंधी<br>a. Skin pustules<br>त्वचा में मवाद युक्त दानें<br>b. Umbilicus draining pus<br>नाभी से मवाद का रिसाव<br>c. Umbilicus with boil (s)<br>नाभी पर फोड़ा होना		

Eye Related<br>आंख सम्बंधी<br>a. Eyes draining pus<br>आंखों से कीचड़ आना		
Oral cavity Related<br>**मुख सम्बंधी**<br>a. Tongue having white patches<br>जीभ पर सफेद चकत्ते<br>b. Any other patch in mouth<br>मुख में अन्य प्रकार के चकत्ते		

Q.6 **When will you consider that the infant is not feeding well?**
आप यह कब तय करेंगें कि बच्चा ठीक तरह से आहार ग्रहण नहीं कर पा रहा है?
Probes: Sucking, Feeds number of times/day, Sleeping after the feeds, Hungry after feeds
पूछें : मां का दूध पीना, दिन में कितनी बार पीता है, दूध पीने के बाद सो जाता है, पेट खाली रहता है।

Section 6.1*: Breast Feeding Practices
भाग- 6.1 स्तनपान कराने का व्यवहार

**6.1.1**	***How soon after the birth you breastfed the baby?*** *जन्म के कितने समय पश्चात आपने शिशु को स्तनपान करया?*	***Less than 1 hour$_1$ / 1-4 hours$_2$ / 4- 24 hours$_3$/ >24 hours$_4$/ Never$_5$***<br>*एक घंटें से कम$_1$/1-4 घंटे$_2$/4-24 घंटें$_3$/>24 घंटे$_4$/कभी नही$_5$*	[ ]
**6.1.2**	***If never breastfed than why?***<br>*यदि कभी स्तनपान नही कराया तो क्यों? विवरण लिखें।*<br>***Note verbatim***	--<br>--<br>--<br>***(English Translation)***<br>--<br>--<br>--	
**6.1.3**	***Did you give anything other than breast milk immediately after birth?***<br>*जन्म के तुरन्त बाद क्या आपने शिशु को माँ के दूध के अतिरिक्त कुछ और दिया?*	***Not given$_1$/ Honey or `ghutti` or jaggery$_2$/ Water$_3$/ Any other$_4$ (specify)___________***<br>*नही दिया$_1$/शहद या घुट्टी या गुड$_2$/पानी $_3$/अन्य $_4$ उल्लेख करे.....*	[ ]
**6.1.4**	***Did you give the baby the first milk?***<br>*क्या आपने शिशु को अपना पहला पीला गाढा दूध पिलाया?*	***Yes$_1$*** *हाँ$_1$* ***(go to Q. 5.6)***<br>***No$_2$*** *नही$_2$*	[ ]
**6.1.5**	***If NO, give reasons for it***<br>*यदि नही, इसका कारण बतायें।*	***It is unsafe$_1$/ Due to social customs$_2$/ Advised to discard it$_3$/Others$_4$ (specify) ……………………...***<br>*यह असुरक्षित है$_1$/सामाजिक रीति-रिवाज के कारण$_2$/इसको फेंकने की सलाह दी गयी$_3$/ अन्य$_4$ उल्लेख करें.........................*	[ ]
**6.1.6**	***How frequently did you breastfeed your baby?***<br>*कितने समय के अन्तराल पर आप अपने बच्चें को दूध पिलाती है?*	***On demand $_1$/Every 2 hours$_2$ / Less than 8 times per day$_3$/More than or equal to 8 times per day$_4$***<br>*बच्चें की माग पर$_1$/ प्रति दो घंटें पर$_2$/प्रतिदिन 8 बार से कम$_3$/प्रति दिन 8 बार या उससे अधिक$_4$*	[ ]
**6.1.7**	***Is the baby exclusively breastfed (not even water)?***<br>*क्या आपने बच्चें को सिर्फ स्तनपान ही कराया? (पानी भी नही)*	***Yes$_1$*** *हाँ$_1$*<br>***No$_2$*** *नही$_2$*	[ ]

6.1.8	***If baby is not being breastfed, then which method you are using to feed the baby?*** *यदि बच्चें ने स्तनपान नही किया, तब बच्चे को आहार देने हेतु आप कौन सी विधि का प्रयोग कर रही है।*	***Spoon feeding$_1$/ Bottle feeding$_2$/Others$_3$ specify ________________________/*** ***Not applicable (as baby only breastfed) $_4$*** *चम्मच द्वारा$_1$/बोतल द्वारा$_2$/अन्य कोई$_3$उल्लेख करें...............* */लागू नहीं (बच्चा केवल स्तनपान ही करता है)$_4$*	***[ ]***
6.1.9	***What is the frequency of urination of baby?*** *बच्चा कितने-कितने अन्तराल पर पेशाब करता है।*	***After every feed$_1$/ 1 – 4 times per day$_2$/ 5 – 10 times per day$_3$/Other$_4$ Specify______________*** *प्रत्येक आहार के बाद$_1$/प्रतिदिन 1-4 बार$_2$/प्रतिदिन 5-10 बार$_3$/ अन्य कोई$_4$ उल्लेख करें..............*	***[ ]***

6.2*	***Which feeds has the infant had in the previous 24 hours?*** *पिछले २४ घंटे में शिशु ने क्या खाया*	***Yes*** *हाँ*	***No*** *नहि*	***Don't Know*** *पता नहीं*	***How many times?*** *कितनी बार*
A	***Breastmilk*** *स्तनपान*	***[ ]***	***[ ]***	***[ ]***	
B	***Bottle Feed*** *बोतल का दूध*	***[ ]***	***[ ]***	***[ ]***	
C	***Top Milk*** *ऊपर का दूध*	***[ ]***	***[ ]***	***[ ]***	
D	***Plain Water*** *सादा पानी*	***[ ]***	***[ ]***	***[ ]***	
E	***Juice or juice drinks*** *जूस*	***[ ]***	***[ ]***	***[ ]***	
F	***Yogurt*** *दही*	***[ ]***	***[ ]***	***[ ]***	
G	***Formula feed*** *दब्बे का दूध*	***[ ]***	***[ ]***	***[ ]***	
H	***Any other liquids?*** *कोइ और पीने की चीज*	***[ ]***	***[ ]***	***[ ]***	
6.3*	***Has the infant been fed expressed breastmilk?*** *क्या बच्चे को मां का दूध निकाल कर पिलाया है*				
6.4*	***How many times has the infant fed in the past 24 hours?*** *पिच्ले 24 घंते मैं शिशु को कित्नि बार कुच दिया*				
6.5*	***Has the infant been given any medicines or vitamins in 24 hours?*** *क्या शिशु को पिच्ले 24 घंते मैं कोइ दवै दी*				

6.6*	***If an infant has feeding problem who can you go to for advice?*** *अगर शिशु को दूध पीने मैं दिक्कत हो तोह किस्के पास लेके जाते हैं*	***Spontaneous*** *अपने आप बताया*	***Prompted*** *पूछने पे बताया*
A	***Elders in family*** *परिवार में बड़े बुजुर्ग*	***[ ]***	***[ ]***
B	***Elders in village*** *गॉव में बड़े*	***[ ]***	***[ ]***
C	***ASHA*** *आशा*	***[ ]***	***[ ]***
D	***ANM*** *अए एन एम*	***[ ]***	***[ ]***
E	***Doctor*** *चिकित्सक*	***[ ]***	***[ ]***
F	***Other (Specify)*** *कोइ और (उल्लिखित करेन)*	***[ ]***	***[ ]***

Q.7 **From whom did you get information about symptoms of sickness in young infants**?
(Tick all that apply)
नवजात में बीमारी के लक्षणों की जानकारी आपको किन स्रोतों से प्राप्त हुई?

Seen a similar case in neighborhood<br>पड़ोस में इसी तरह का केस देखा		[ ]	
1.	Had a similar case in family<br>a. Any of the previous child of her own<br>स्वयं का पहले का बच्चा<br>b. Any other child in family<br>परिवार में अन्य दूसरा बच्चा	 [ ]<br><br>[ ]	
2.	*Got information through :*<br>(multiple options)<br>सूचना किसके द्वारा प्राप्त हुई (बहुविकल्पीय)	1. ASHA आशा	[ ]
		2. ANM ए0एन0एम0	[ ]
		3. Doctor चिकित्सक	[ ]
		4. Media मीडिया	[ ]
		5. Index/other infant sick<br>चिन्हित/ अन्य बीमार नवजात शिशु	[ ]
		6. Any other<br>अन्य	[ ]
3*	***Have you contacted ASHA for any illness in the infant? (Yes / No)***<br>***क्या आप बीमारी के लिये आशा को मिलते हैं***	[ ]	
A	***If yes, how many times?***<br>***अगर हाँ तो कितनी बार***	[ ]	
4*	***Have you met the ANM for the infant? (Yes / No)***<br>***क्या शिशु के लिये ए एन एम से मिले***	[ ]	
A	***If yes, how many times?***<br>***अगर हाँ तो कितनी बार***	[ ]	
B	***How many of these visits were for an illness in the infant?***<br>***इन मैं से कितनी बारी शिशु की बीमारी के लिये गये***	[ ]	

Q.8 **Does ASHA come to examine mother and young infant at home?**
क्या आशा माँ एवं नवजात शिशु की जांच हेतु घर आती है।

Yes हां	[ ]
No नही	[ ]
Don't know पता नहीं	[ ]

If yes, यदि हां	Write verbatim कथन लिखें
a. What does she do?<br>वह क्या करती है?	
b. Do you know how many times/when?<br>क्या आपको पता है कि कितनी बार आयी और कब आयी?	

Q.9 **If a young infant gets sick, what should be done?**
यदि शिशु बीमार पड़ता है, क्या करना चाहिए?

Possible Options<br>सम्भावित विकल्प	If so then for what symptoms, when, why?<br>यदि ऐसा है तो कौन से लक्षण में, कब एवं क्यों?<br>*(Write verbatim)* (कथन लिखें)	*For office use only*<br>कार्यालय प्रयोग हेतु
a. Wait and watch<br>इंतजार करेगें और देखेंगें		

b. Call or visit ASHA/ANM<br>आशा अथवा ए0एन0एम0 को बुलायेंगें या दिखाएंगें		
c. Take to Government hospital<br>सरकारी अस्पताल ले जाएंगें		
d. Take to private hospital<br>निजी अस्पताल ले जायेंगें		
e. Take to RMP<br>गांव के चिकित्सक के पास जाऐंगें।		
f. Any other, Specify<br>अन्य कोई, स्पष्ट करें		

1. Date of Interview: [ ] [ ] / [ ] [ ] / [ ] [ ] [ ] [ ]
   dd mm yyyy
2. Start Time: [ ] [ ] [ ] [ ] (24 Hour format)
3. End time of Interview: [ ] [ ] [ ] [ ] (24 Hour format)
4. Place of Interview: ____________________________________

   Coding *(for office use)*____________)
5. Interviewer Signature: __________________
6. Supervisor Signature: ___________
7. Name Interviewer (Initials only): [ ] [ ]

   Staff code: [ ] [ ]
8. Name Supervisor (Initials only): [ ] [ ]

   Staff code: [ ] [ ]

	Reflections
Reflection on interview<br>(Comment on Body Language, Reliability, Unsaid Information, Self deductions beyond verbal communications etc.)	
Level of Co-operation<br>(Scale 1 – 5)	*(Mark at the appropriate level)*<br>1 2 3 4 5<br>(Not co-operative) (Extremely co-operative)
Number of Interruptions during interview with reasons	

Annexure

Q10: List all live births (Chronologically)

सभी जीवित जन्मों की सूची (कालानुक्रमिक)

Child number बच्चे का क्रम	When was the child born (year) बच्चे का जन्म कब हुआ (साल में)	Place of delivery(Home[1]/ Private Institution[2]/Govt Institution[3]/ /Transit[4]) प्रसव का स्थान (घर [1] / निजी संस्थान[2]/सरकारी संस्थान [3]/ रास्ते में [4])	Immunization Status (Fully Immunized for age [1]/Not Immunized[2]) टीकाकरण स्थिति (पूरी तरह से उम्र के लिए प्रतिरक्षित[1] / प्रतिरक्षित नहीं[2])	Living on day of interview (Yes/No) बच्चा साक्षात्कार के दिन जीवित है (हा/नही)	Age (today/at death) If less than 1 year then please specify the age in months आयु (आज / मृत्यु पर) यदि 1 वर्ष से कम हो तो कृपया महीनों में उम्र लिखें	If dead, Place of death (Home[1]/ Private Institution[2]/ Govt. Institution[3]/ /Transit[4]) अगर मृत , मृत्यु का स्थान (घर [1] / निजी संस्थान/ सरकारी संस्थान [2] / रास्ते में [3])	If dead, Cause of death if known अगर मृत, मृत्यु का कारण	If died following an illness, after how many days यदि बीमारी के बाद मृत्यु हो गई है, तो कितने दिन बाद
1								
2								
3								
4								
5								

Q 11. Any other information shared by the family regarding the child's death:

बच्चे की मृत्यु के बारे में परिवार द्वारा साझा की गई कोई अन्य जानकारी

____
____
____
____
____
____
____
____
____
____
____
____
____
____
____
____
____
____

Q12. Where do you usually cook food(indoor[1]/outdoor[2]) [ ]

आप आमतौर पर खाना खाना कहा बनाते हैं अन्दर[1] बाहर [2]

Q13. What fuel do you use for cooking (Electricity/LPG/Natural gas/Biogas[1] or Firewood[2]/ coal/Charcoal[3]/cowdung cakes[4]/kerosene[5] [ ]

खाना पकाने के लिए आप किस ईंधन का उपयोग करते हैं (बिजली / एलपीजी / प्राकृतिक गैस / बायोगैस [1] या जलाऊ लकड़ी[2] / कोयला / चारकोल [3] / गाय का गोबर[4] / मिटटी तेल [5]
