# Supplementary material for: Management of possible serious bacterial infection in young infants closer to home when referral is not feasible: Lessons from implementation research in Himachal Pradesh, India
Source: PLoS One. 2020 Dec 22;15(12):e0243724. doi: 10.1371/journal.pone.0243724 (PMC7755274; doi:10.1371/journal.pone.0243724)
Supplement: S1 IDI guide — (PDF) [file pone.0243724.s013.pdf]

In-depth interviews (IDI): ANM

All ANMs in the block will be interviewed to get an understanding about their work and responsibilities, their experience in implementation of the simplified regime for management of possible serious bacterial infection in sick young infants when referral is not possible, their knowledge on identification of young infants with PSBI, their thoughts on the community awareness activities, challenges faced and their suggestions and thoughts on improving the implementation.

INTRODUCTION

Namaste. My name is ____________.

Our team had called earlier to take time from you for this interview. Thank you for giving us the time. We would like to take your consent for recording our conversation to ensure that we do not miss anything.

EXPERIENCE ON THE IMPLEMENTATION

1. Can you tell me about your work?

    What are your primary responsibilities?

    How many SCs are under your charge?

    How many ASHA workers do you supervise?

2. Can you describe a routine day at work?

    Is your routine at work the same throughout the week? *(Are there different activities conducted on different days)*

    How long are you available in your assigned sub-centre in a day/week/month?

3. Do families of young infants (0-59 days) visit your Sub-centre?

    If young infants visit the sub-centre:

    How many infants have you seen in last one year/month/week?

    How many infants had sign and symptoms of PSBI?

    How many were sent by ASHAs?

    How many came on their own?

    If young infants do not visit the sub-centre:

    Why do you think families do not bring young infants to your sub-centre?

4. Do you know the sign and symptoms of PSBI? Can you tell us?
5. Do you have the IMNCI chart booklet?

    Do you use the chart booklet for identification, classification and treatment of young infant?

    If no, why not?

6. Are you confident in your ability to manage case of PSBI?
7. Do you counsel the mother after giving treatment?
8. In your opinion is training that you received adequate? Do you think any additional training is required? If so, can you specify?
9. In your opinion, what improvements can be made to your health sub-centre to improve the care of sick infants?
10. Do you conduct any awareness activities at your sub-centre/catchment area?

    If awareness activities conducted:

    What is your opinion of the community awareness activities?

    When were the awareness activities conducted?

    How many times were these awareness activities conducted in the last 6/3 months?

    Who conducted these awareness activities?

    If awareness activities not conducted:

    Why were you not able to conduct awareness activities?

    Do you need any support to conduct awareness activities? Can you tell us what support you need?

11. How do you come to know whether the ASHAs in your area conduct the HBNC visits?
12. Are you able to supervise these HBNC visits and activities?

    How often are you able to supervise?

    How many accompanied visits were you able to make in the last one month?

13. What is the process of reporting death in infants (0-1 yr)?

    How and where do you get this information from?

    How and when do you report it?

    How do you ascertain the cause of death?

14. Do you face any challenges in the implementation of this project in your sub-centre area? Please explain.
15. Based on your knowledge and experience, what could be the issues faced if this project is also implemented in the neighbouring blocks?
