# Supplementary material for: Management of possible serious bacterial infection in young infants closer to home when referral is not feasible: Lessons from implementation research in Himachal Pradesh, India
Source: PLoS One. 2020 Dec 22;15(12):e0243724. doi: 10.1371/journal.pone.0243724 (PMC7755274; doi:10.1371/journal.pone.0243724)
Supplement: S2 IDI guide — (PDF) [file pone.0243724.s014.pdf]

In-depth interviews (IDI): ASHA Workers

A list of all ASHAs in the block will be made. At least half of all ASHAs under each PHC will be selected for the interview depending on their availability and consent. The purpose of this interview is to get an understanding about their work and responsibilities, their experience and knowledge in identification of sick young infants with possible serious bacterial infection, challenges faced, training requirements and their suggestions and thoughts on improving the identification of sick young infants.

INTRODUCTION

Namaste. My name is ____________.

Our team had called earlier to take time from you for this interview. Thank you for giving us the time. We would like to take your consent for recording our conversation to ensure that we do not miss anything.

EXPERIENCE ON THE IMPLEMENTATION

1. Can you tell me about yourself and your work
2. Can you tell me about the HBNC visits you conduct

   Do you have the HBNC home visit forms?

   If you have the forms:

   Do you fill the form for each child? Do you fill them for each home visit?

   (When? during visit, after visit, after coming home)

   If you do not fill it, what is the reason behind it? *(What are the difficulty/challenges in filling the form: Form takes too long to fill, difficult to reach families, does not know how to fill, etc)*?

   After filling the form, when and whom do you give the form to?

   If you do not have the forms:

   How do you record the information regarding the HBNC visits that you do?
   Can you tell me why you do not have the forms?
3. Have you visited any infant aged 0-2 months in the last 6/3 months?

   If yes, how many infants did you visit or examine

   Did any of the infants have danger signs? (*How many, which danger signs).*

   What advice did you given to the family of the infant with the danger sign?

   Did the family follow the advice? If yes/no, can you please tell me the details?

Did the ANM/MO confirm the presence of the danger sign in the infant identified by you?

4. Can you tell me the danger sings in young infants?
5. Have you attended any trainings? Have the trainings made any difference or change*(in your work, your confidence and how people perceive you)*
6. Do you think you need any other training?
7. Do you face any challenges in conducting HBNC visits (*supplies in HBNC visit, registers, incentives)*
8. Do you have any suggestion on what can help you in your work?
