# Supplementary material for: Management of possible serious bacterial infection in young infants closer to home when referral is not feasible: Lessons from implementation research in Himachal Pradesh, India
Source: PLoS One. 2020 Dec 22;15(12):e0243724. doi: 10.1371/journal.pone.0243724 (PMC7755274; doi:10.1371/journal.pone.0243724)
Supplement: S3 IDI guide — (PDF) [file pone.0243724.s015.pdf]

In-depth interviews (IDI): Medical Officers

All MOs in the block will be interviewed to find out about their experience in implementation of the simplified regime for management of possible serious bacterial infection in sick young infants when referral is not possible, the challenges faced and their suggestions and thoughts on improving the implementation.

INTRODUCTION

Namaste. My name is ____________.

Our team had called earlier to take time from you for this interview. Thank you for giving us the time. We would like to take your consent for recording our conversation to ensure that we do not miss anything.

EXPERIENCE ON THE IMPLEMENTATION

1. Please tell me about your work
2. Do families with young infants (0-59 days) visit your health centre?

    If young infants visit the health centre:

    How many infants do you see in a month (on an average)?

    How many of these infants have sign and symptoms of PSBI?

    How many were sent to your health centre by ASHAs?

    How many were sent to your health centre by ANMs?

    How many came on their own?

    Do you use IMNCI chart booklet to classify sick infants and choose their treatment?

    If yes, then in your opinion, is there any aspect of the chart booklet which could be improved?

    If no, why is that? (*lost booklet, did not understand*)

    Do you give pre-referral dose before referring a case?

    What advice do you give to families regarding care of the child during referral?

    If young infants do not visit the health centre:

    Why do you think children do not come to your health centre?

    What do you think should be done to raise community awareness and/or encourage people to bring their sick infants to your health centre?

3. Do you have any difficulties in maintaining different PSBI records? (*Treatment card, IMNCI recording form, Annexure to treatment card, etc*). Elaborate on any issues faced and any suggestions for improvement.
4. In your opinion, what improvements can be made to your health centre to better cater to sick infants?
5. Do you face any challenges in the care and referral of sick infants and/or implementation of this project? Please specify.
6. Do you have any suggestions for improving the IMNCI training sessions? How frequently in your opinion, are revision sessions needed?
7. Do you have any suggestions for how community awareness and demand generation activities can be done more effectively in your (or adjacent) blocks?
8. Based on your knowledge and experience, do you have any suggestions on what needs to be done differently if this project is also implemented in the neighbouring blocks and districts?
